# Stars and stripes: hexatopic tris(3,2′:6′,3′′-terpyridine) ligands that unexpectedly form one-dimensional coordination polymers†

**DOI:** 10.1039/d1ce01531a

**Published:** 2021-12-06

**Authors:** Giacomo Manfroni, Alessandro Prescimone, Edwin C. Constable, Catherine E. Housecroft

**Affiliations:** Department of Chemistry, University of Basel Mattenstrasse 24a, BPR 1096 4058-Basel Switzerland catherine.housecroft@unibas.ch

## Abstract

The hexatopic ligands 1,3,5-tris(4,2′:6′,4′′-terpyridin-4′-yl)benzene (1), 1,3,5-tris(3,2′:6′,3′′-terpyridin-4′-yl)benzene (2), 1,3,5-tris{4-(4,2′:6′,4′′-terpyridin-4′-yl)phenyl}benzene (3), 1,3,5-tris{4-(3,2′:6′,3′′-terpyridin-4′-yl)phenyl}benzene (4) and 1,3,5-trimethyl-2,4,6-tris{4-(3,2′:6′,3′′-terpyridin-4′-yl)phenyl}benzene (5) have been prepared and characterized. The single crystal structure of 1·1.75DMF was determined; 1 exhibits a propeller-shaped geometry with each of the three 4,2′:6′,4′′-tpy domains being crystallographically independent. Packing of molecules of 1 is dominated by face-to-face π-stacking interactions which is consistent with the low solubility of 1 in common organic solvents. Reaction of 5 with [Cu(hfacac)_2_]·H_2_O (Hhfacac = 1,1,1,5,5,5-hexafluoropentane-2,4-dione) under conditions of crystal growth by layering resulted in the formation of [Cu_3_(hfacac)_6_(5)]_*n*_·2.8*n*C_7_H_8_·0.4*n*CHCl_3_. Single-crystal X-ray diffraction reveals an unusual 1D-coordination polymer consisting of a series of alternating single and double loops. Each of the three crystallographically independent Cu atoms is octahedrally sited with *cis*-arrangements two N-donors from two different ligands 1 and, therefore, *cis*-arrangements of coordinated [hfacac]^−^ ligands; this observation is unusual among compounds in the Cambridge Structural Database containing {Cu(hfacac)_2_N_2_} coordination units in which the two N-donors are in a non-chelating ligand.

## Abstract 2

Five hexatopic ligands containing 3,2′:6′,3′′- or 4,2′:6′,4′′-terpyridine domains are reported, along with the assembly of a 1D-coordination polymer consisting of alternating single and double loops.

## Graphical abstract

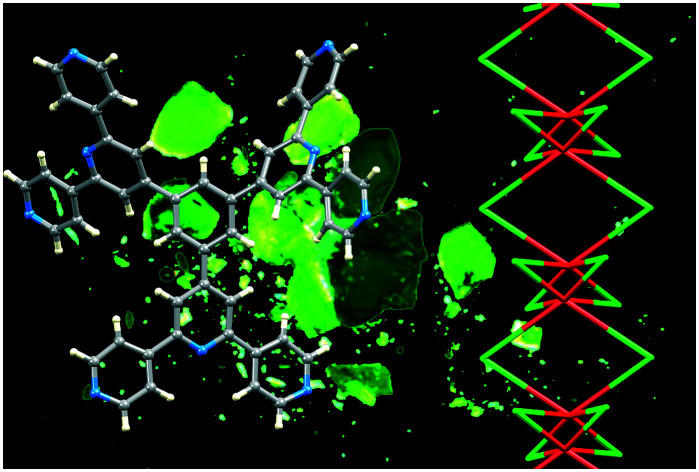

## Introduction

Coordination polymers (CPs)^1^ have a long history, stretching back at least to the discovery of Prussian blue at the beginning of the 18th century.^2^ However, it is only during the last three decades that CPs have been the focus of intense research attention.^3^ The possibility of performing routine single crystal structural determinations is one of the key factors and, since then, widespread potential applications have included catalysis,^4,5^ gas capture,^6,7^ molecular separations,^8–11^ proton conduction^12^ and sensing.^13,14^

Oligopyridines (N-donors) and polycarboxylates (O-donors) are among the most common classes of organic ligands for CPs. Terpyridine (tpy) ligands are well-established N-donors in coordination chemistry, and have therefore long been successfully exploited for the assembly of discrete and polymeric coordination complexes.^15^ Of the 48 possible isomeric terpyridines, 2,2′:6′,2′′-tpy is the archetypal. It is a monotopic chelating ligand, although the 2:2′:6′,2′′-tpy domain also exhibits multiple metal-binding modes.^16^ CPs are formed by combinations of metal nodes and ligand linkers, metal linkers and ligand nodes, or metal linkers and ligand linkers. Thus, if the organic ligand contains a 2:2′:6′,2′′-tpy metal-binding domain, a second-binding domain, such as carboxylate or a heterocycle, is needed to generate an {M(tpy)_2_} centred ‘expanded ligand’.^17^

**Scheme 1:**
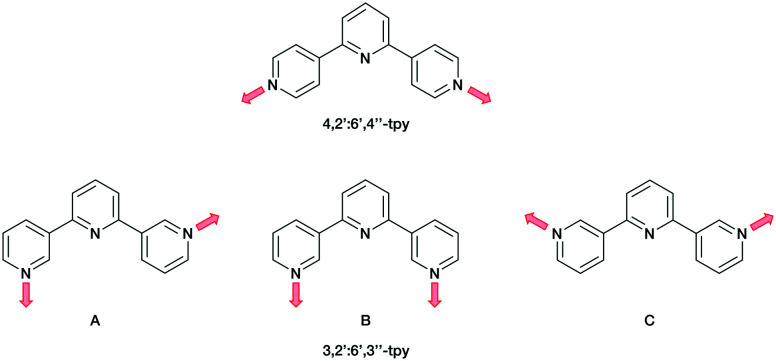
The fixed coordination directionality in 4,2′:6′,4′′-tpy, compared with the three planar conformations A, B and C of 3,2′:6′,3′′-tpy.

A contrasting approach is to employ oligopyridines with divergent sets of donor atoms. Of all the isomers, however, only the symmetrical 4,2′:6′,4′′-tpy and 3,2′:6′,3′′-tpy have been widely used in the last decade,^15,18–22^ along with their 4′-functionalized derivatives which are readily accessible using either Wang and Hanan's one-pot synthetic approach^23^ or the Kröhnke methodology.^24^ The ligands 4,2′:6′,4′′-tpy and 3,2′:6′,3′′-tpy coordinate through the outer pyridine donors, leaving the central pyridyl-N atom unbound (Scheme 1). CPs with uncoordinated Lewis-base sites within solvent-accessible channels have been considered as potential candidates for small-molecule and ion detection applications,^15,22,25–27^*e.g.* through C–H⋯N pyridine hydrogen bond formation.^15^ These tpy isomers possess different vectorial properties of the N-donor set (Scheme 1). While 4,2′:6′,4′′-tpy offers a fixed V-shaped metal-binding domain, the less explored isomer 3,2′:6′,3′′-tpy has a greater conformational flexibility which arises from rotation about the inter-ring C–C bonds connecting the pyridine rings leading to a more variable and less predictable network assembly.^21^

**Scheme 2:**
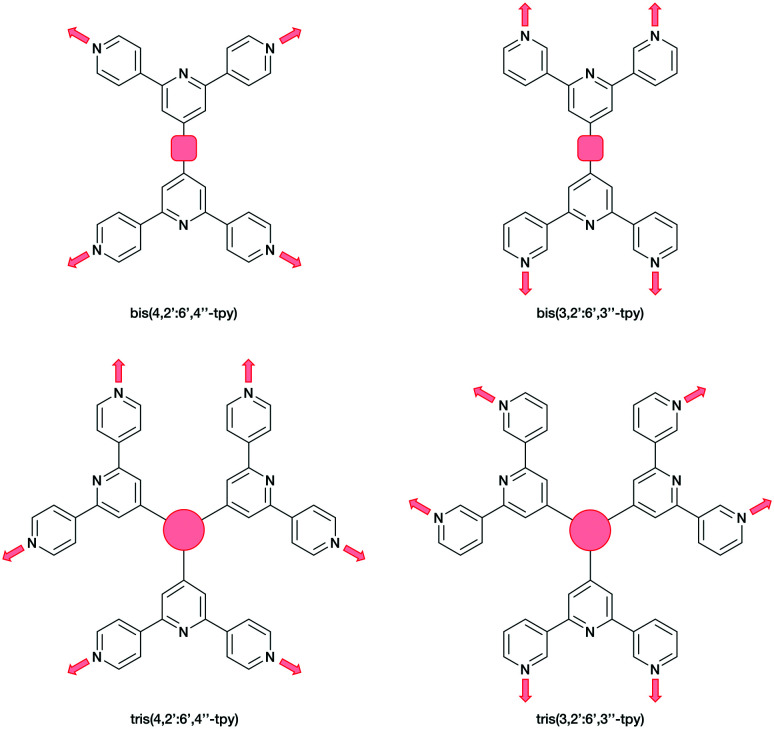
Tetratopic and hexatopic ligands resulting from connecting two and three 4,2′:6′,4′′-tpy or 3,2′:6′,3′′-tpy domains. The red spacer represents any organic linker. Only one of three possible planar conformations of 3,2′:6′,3′′-tpy is shown.

Ditopic ligands such as those in Scheme 1 have a limited role as 2-connecting linkers and by connecting multiple tpy domains, the ligands can act as directing nodes in the assembly of multidimensional architectures. Two 4,2′:6′,4′′-tpy or 3,2′:6′,3′′-tpy units, connected by an organic spacer, have the capability to function as 4-connecting nodes (Scheme 2).^20–22,28^ Substituents, such as *n*-alkyloxy groups, enhance the solubility of the ligand. However, the chain length and shape have a profound impact in orienting the assembly.^20,21,28^ Yoshida and co-workers reported the synthesis and structural determination of the first coordination polymer with a bis(4,2′:6′,4′′-tpy) in 2013,^29^ and further examples followed.^20,28^ However, the coordination behaviour of bis(3,2′:6′,3′′-tpy) ligands remains little explored.^29–31^

**Scheme 3:**
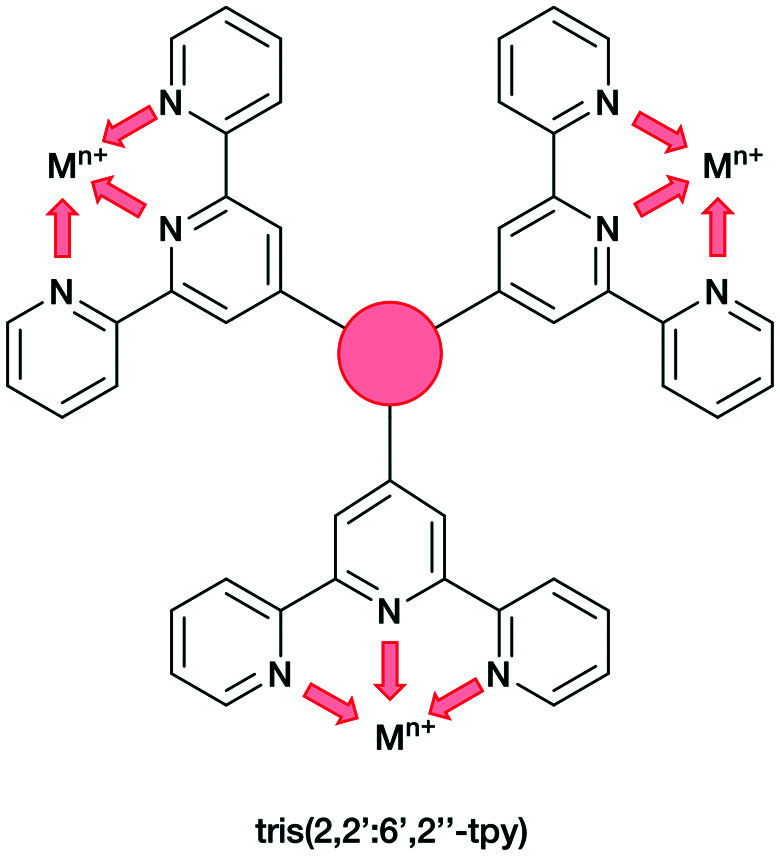
Tris(2,2′:6′,2′′-tpy) as tritopic ligand. Each tpy domain is monotopic, with the three tpy nitrogen atoms binding only one metal ion.

In the present work, we extend our investigations of tetratopic bis(3,2′:6′,3′′-tpy) and bis(4,2′:6′,4′′-tpy) to hexatopic tris(3,2′:6′,3′′-tpy) and tris(4,2′:6′,4′′-tpy) ligands. To date, no tris(4,2′:6′,4′′-tpy) nor tris(3,2′:6′,3′′-tpy) ligands has been reported, despite the fact that they are attractive building blocks for coordination assemblies (Scheme 2). However, this design principle has been exploited with 2,2′:6′,2′′-tpy metal-binding domains and, from the first tris(2,2′:6′,2′′-tpy) in 1992,^32^ a wide spectrum of poly(2,2′:6′,2′′-tpy) ligands has been used for metallosupramolecular constructs.^33,34^ This is reflected in a range of complex geometries, such as discrete 2D-^35^ and 3D-fractals,^36^ metallodendrimers^37^ and metallocages^38–40^ (*e.g.* molecular polyhedra).^41^ Some 20 years ago, we introduced the term metallostar to describe such polynuclear assemblies^42–46^ and the concept of using tritopic tris(2,2′:6′,2′′-tpy) ligands (Scheme 3) for the growth of 2D or 3D CPs was developed by us and others,^47,48^ and the potential in materials chemistry subsequently demonstrated.^49–58^ The groups of Nishihara,^56,57^ Wong^54^ and Chakraborty^51,55^ showed a series of coordination nanosheets using different tris(terpyridines) in which durable electrochromism was observed with Fe^2+^ or Co^2+^. Moreover, according to Nishihara's group, anion exchange capacities and solvatoluminochromic behaviour were found with Zn(BF_4_)_2_ and ZnSO_4_ respectively.^58^ With 1,3,5-tris[4-(2,2′:6′,2′′-terpyridin-4′-yl)phenyl]benzene, Higuchi and co-workers described the syntheses of Fe^2+^, Co^2+^ and Ni^2+^-based CPs showing humidity-responsive ionic conduction.^53^ It should be noted that none of these polymers, containing tris(terpyridines), has been unequivocally characterized by single crystal X-ray diffraction. A search of the Cambridge Structural Database^59^ (CSD v. 2021.1.0),^60^ using ConQuest (v. 2021.1.0),^60^ revealed no CPs assembled with tris 2,2′:6′,2′′, 3,2′:6′,3′′ or 4,2′:6′,4′′ terpyridines. We ignore instances where the tpy unit is one of the less common 45 isomers.

**Scheme 4:**
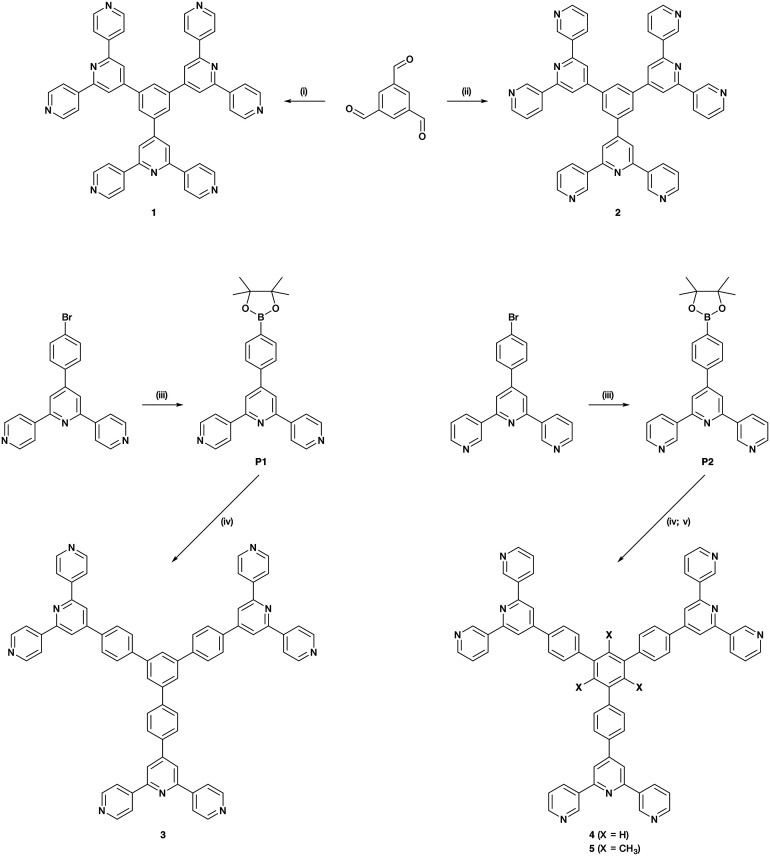
Synthetic route to ligands 1–5; reagents conditions: (i) 4-acetylpyridine, KOH, NH_3_, EtOH, RT, 36 h; (ii) 3-acetylpyridine, KOH, NH_3_, EtOH, RT, 36 h; (iii) bis(pinacolato)diboron, Pd(dppf)Cl_2_, AcOK, DMF, 120 °C, 5 h 30 min; (iv) 1,3,5-tribromobenzene, Pd(PPh_3_)_4_, aqueous Na_2_CO_3_, THF, reflux, 12 h; (v) 2,4,6-tribromomesitylene, Pd(dppf)Cl_2_, Na_2_CO_3_, toluene/H_2_O (1 : 1), reflux, 1 h.

Hence, there is significant scope for investigations of building blocks incorporating three or more 4,2′:6′,4′′-tpy or 3,2′:6′,3′′-tpy domains. Herein, we present the syntheses of a series of star-shaped ligands (Scheme 4). We also describe the self-assembly and single-crystal X-ray characterization of a tris(terpyridine)-based CP to generate an unusual 1D architecture.

## Results and discussion

### Ligand synthesis and characterization

Compounds 1 and 2 were prepared using Wang and Hanan's one-pot protocol^23^ (Scheme 4). After purification, 1 and 2 were obtained with yields of 22 and 13%, respectively. The synthetic route to compounds 3 and 4 was similar to the one used to synthesize the related 1,3,5-tris[4-(4′-2,2′:6′,2′′-terpyridyl)phenyl]benzene.^61^ A Suzuki cross-coupling reaction between 1,3,5-tribromobenzene and 4′-(4-pinacolatoboronphenyl)-4,2′:6′,4′′-terpyridine (**P1**) or 4′-(4-pinacolatoboronphenyl)-3,2′:6′,3′′-terpyridine (**P2**) (Scheme 4) was conducted in refluxing THF, with [Pd(PPh_3_)_4_] as catalyst and aqueous Na_2_CO_3_ as base, affording 3 (37%) and 4 (80%). The compounds were analytically pure and no recrystallization or chromatography was required. However, compounds 3 and 4 were poorly soluble in most common organic solvents which presumably arises from extensive intermolecular stacking interactions. Since ligand solubility is critical for crystallization methods in the assembly of CPs, we moved from ligands 3 and 4 to analogous compounds with solubilizing substituents. Rather than using hindering groups such as long alkyl chains, we opted for methyl groups (ligand 5, Scheme 4). The steric hindrance between adjacent methyl and phenyl groups forces the ligand into a non-planar conformation in the solid state, leading to less efficient π-stacking as is seen in 1,3,5-trimethyl-2,4,6-triphenyl-substituted arenes.^62–66^ An approach using the Suzuki coupling of 2,4,6-tribromomesitylene and **P2** was not successful when utilizing THF and [Pd(PPh_3_)_4_], but an improved route to 5 was found using [Pd(dppf)Cl_2_] and Na_2_CO_3_ in refluxing toluene-water. However, some homo-coupling and dehalogenation by-products were identified by MALDI-TOF mass spectrometry. After chromatographic purification eluting with CHCl_3_, compound 5 was isolated analytically pure in a yield of 33%. Ligands 1–5 and boronic esters **P1** and **P2** were fully characterized by ^1^H and ^13^C{^1^H} NMR spectroscopies (Fig. S1–S20†), UV-vis (Fig. S21–S25†) and ATR-IR spectroscopies (Fig. S26–S33†), MALDI-TOF mass spectrometry (Fig. S34–S40†), and HR-ESI mass spectrometry (Fig. S41–S43†) or elemental analysis (see ESI† for full details). The data were consistent with the structures shown in Scheme 4. Note that ligands 3–5 with the phenylene spacers are poorly soluble in many organic solvents and, for 3, the absorption spectrum had to be recorded in benzonitrile, this being the only suitable solvent with a high-energy cut-off.

### Single crystal structure of 1·1.75DMF

**Fig. 1:**
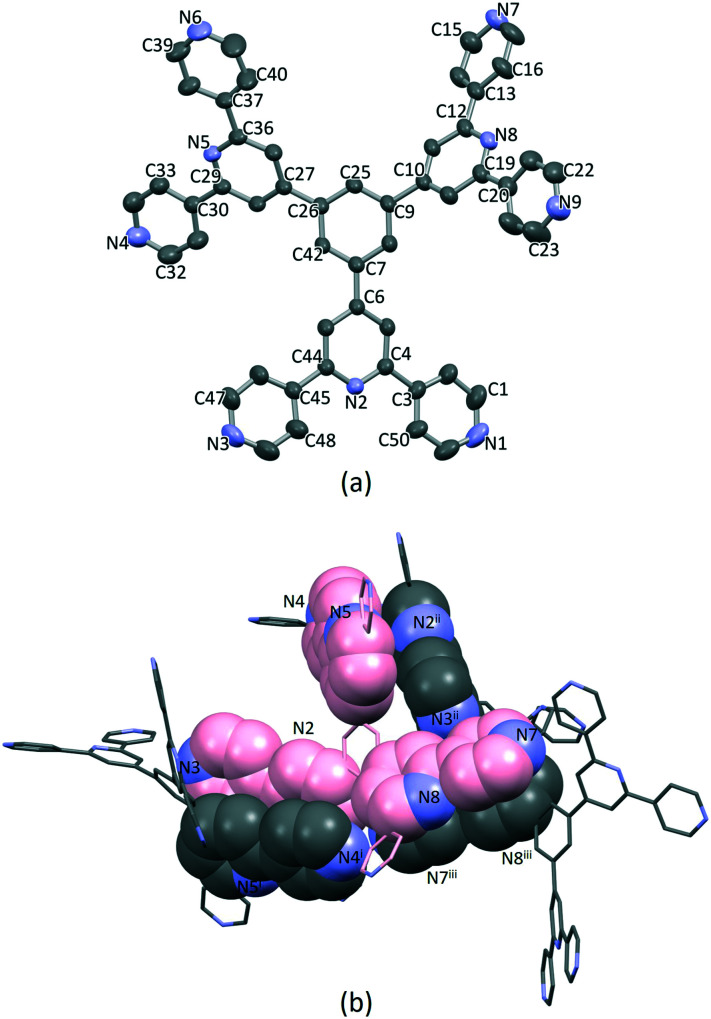
(a) Single crystal X-ray structure of 1, H atoms and DMF molecules are omitted for clarity. Thermal ellipsoids are drawn at 40% of probability level. (b) Face-to-face π-stacking between molecules of 1. The carbon atoms of the crystallographic independent molecule are coloured in pink.

Single crystals of 1·1.75DMF grew as a hot DMF solution of 1 was allowed to cool down to 5 °C. Tristerpyridine 1 crystallizes in the orthorhombic space group *Pbca* and its structure (Fig. 1a) reveals a propeller shaped geometry. The asymmetric unit contains one independent molecule and the three tpy substituents are crystallographically independent. The angles between the plane of the central arene ring and the planes of the pyridine rings containing N2, N5 and N8 (Fig. 1a) are 35.4, 32.8 and 38.0°, respectively. The conformations of the 4,2′:6′,4′-tpy units also differ. For the tpy unit having N1, N2 and N3, the twist angle between the planes of the rings containing N1/N2 and N2/N3 are 9.4 and 10.5°, respectively. In the tpy containing N7, N8 and N9, the angles between the adjacent pyridine rings N7/N8 and N8/N9 are 15.1 and 22.5°, respectively. In contrast, in the tpy possessing N4, N5 and N6, the rings are almost coplanar (angles between N4/N5 and N5/N6 are 6.5 and 1.0°).

Packing of the molecules is dominated by face-to-face π-stacking, which is consistent with the low solubility of 1. For the crystallographic independent molecule, two different intermolecular π-stacking interactions can be classified (Fig. 1b). First, tpy unit with N1, N2 and N3 engages in an interaction with the tpy domain N4^i^, N5^i^ and N6^i^ (symmetry code i = 1 − *x*, −^1^/_2_ + *y*, ^1^/_2_ − *z*). A symmetry related interaction occurs between the tpy containing N4, N5 and N6 and that with N1^ii^, N2^ii^ and N3^ii^ (symmetry code ii = 1 − *x*, ^1^/_2_ + *y*, ^1^/_2_ − *z*). The centroid⋯centroid separations for pairs of rings containing N2/N4^i^ and N3/N5^i^ are 3.98 and 4.43 Å, and the corresponding angles between the ring planes are 18.2 and 21.6°. The third tpy unit containing N7, N8 and N9 stacks with the domain containing N7^iii^, N8^iii^ and N9^iii^ across an inversion centre (symmetry code iii = 1 − *x*, 1 − *y*, 1 − *z*). The centroid separation between rings with N7 and N8^iii^ is 4.21 Å. All of these centroid⋯centroid separations are somewhat longer than is typical for efficient π-stacking interactions.^67^ In addition, the packing of the molecules involves C–H⋯N hydrogen bonds, with H⋯N distances in the range 2.47–2.73 Å (C⋯N range 3.41–3.58 Å, and C–H⋯N angle range 142.3–175.8°). Overall, the packing interactions lead to an intricate 3D-dimensional lattice.

### Single-crystal structures of the coordination polymers [Cu_3_(hfacac)_6_(5)]_*n*_·2.8*n*C_7_H_8_·0.4*n*CHCl_3_

Single crystals of [Cu_3_(hfacac)_6_(5)]_*n*_·2.8*n*C_7_H_8_·0.4*n*CHCl_3_ were grown under ambient conditions (see Experimental section). Structural analysis revealed the formation of [Cu_3_(hfacac)_6_(5)]_*n*_·2.8*n*C_7_H_8_·0.4*n*CHCl_3_ which crystallises in the monoclinic space group *P*2_1_/*n*. Numerous crystallization set-ups were attempted with different concentrations of toluene solutions of [Cu(hfacac)_2_]·H_2_O and chloroform solutions of 5. The crystals obtained from these attempts had analogous cell parameters to [Cu_3_(hfacac)_6_(5)]_*n*_·2.8*n*C_7_H_8_·0.4*n*CHCl_3_ (see Experimental).

Single crystals were also grown by layering a 1,2-dichlorobenzene solution of [Cu(hfacac)_2_]·H_2_O over a CHCl_3_ solution of 5 containing anthracene. The latter was added as a potential guest molecule in the assembly. A preliminary structural analysis revealed the formation of solvated [Cu_3_(hfacac)_6_(5)]_*n*_, which crystallises in the same monoclinic space group (*P*2_1_/*n*) as [Cu_3_(hfacac)_6_(5)]_*n*_·2.8*n*C_7_H_8_·0.4*n*CHCl_3_ with similar cell dimensions (*a* = 20.693(4) Å, *b* = 21.145(4) Å, *c* = 34.404(7) Å, *β* = 104.72(3)°). The structure suffered severe disorder, but preliminary data confirmed the assembly of a 1D-coordination polymer that was essentially isostructural with that in [Cu_3_(hfacac)_6_(5)]_*n*_·2.8*n*C_7_H_8_·0.4*n*CHCl_3_.

**Fig. 2:**
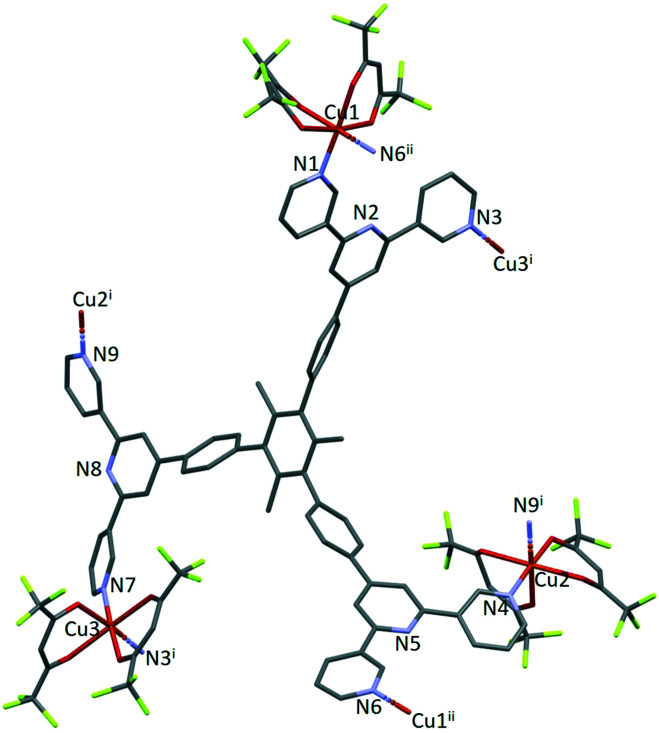
The repeat unit in [Cu_3_(hfacac)_6_(5)]_*n*_·2.8*n*C_7_H_8_·0.4*n*CHCl_3_ with symmetry-generated atoms. For clarity, H atoms and solvent are omitted, and only major occupancies are shown. Symmetry codes: i = 1 − *x*, 1−*y*, 1 − *z*; ii = 1 − *x*, −*y*, 1 − *z*.

The structure of the asymmetric unit in [Cu_3_(hfacac)_6_(5)]_*n*_·2.8*n*C_7_H_8_·0.4*n*CHCl_3_ is shown in Fig. S44.† The backbone of the coordinated [hfacac]^−^ ligand containing C108 and C109, CF_3_ groups having C87, C117, C118 and C122, the phenyl spacers containing the atoms C15–C18 and C24–C27, the pyridine ring containing N7, and solvent molecules are disordered and only major occupancies are shown in Fig. S44.† Disordered parts of the [hfacac]^−^ ligands were refined isotropically with geometrical restraints. Fig. 2 shows the repeat unit of the coordination polymer with symmetry-generated atoms; it contains three independent copper atoms and one independent ligand 5.

**Fig. 3:**
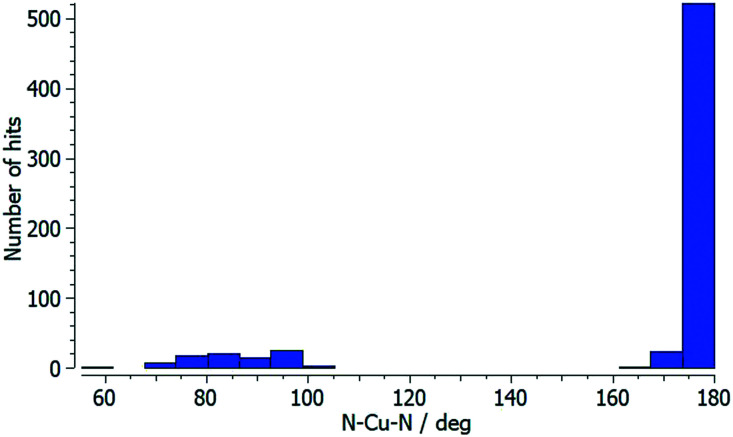
Distribution of the N–Cu–N coordination angle in {Cu(hfacac)_2_(N_1_)(N_2_)} units from a search of the Cambridge Structural Database (v. 2021.1.0) using ConQuest (v. 2021.1.0).

Each of atoms Cu1, Cu2 and Cu3 is octahedrally coordinated with *cis*-arrangements of coordinated [hfacac]^−^ ligands. Each Cu atom binds to two pyridine donor atoms of two different ligands 5. The bond lengths and angles for the coordination spheres of Cu1, Cu2 and Cu3 are given in Table 1. A search of the Cambridge Structural Database^59^ (CSD, v. 2021.1.0 using Conquest v. 2021.1.0)^60,68^ for structures containing in {Cu(hfacac)_2_(N_1_)(N_2_)} units reveals that in most structures the N–Cu–N angles are close to 180° (Fig. 3.), *i.e.* a *trans*-arrangement of N atoms. Most of the *cis* configurations are associated with chelating ligands. Hence, the *cis*-arrangement found in [Cu_3_(hfacac)_6_(5)]_*n*_·2.8*n*C_7_H_8_·0.4*n*CHCl_3_ is considered to be unusual.

**Table 1:** Selected bond lengths and angles in the copper(ii) coordination spheres

Metal atom	Cu–N/Å	N–Cu–N/°	Cu–O/Å	N–Cu–O/°
Cu1	2.013(5)	91.1(2)	2.320(4)	92.6(2), 90.6(2), 177.7(2), 94.69(19), 90.91(18), 175.1(2), 90.6(2), 93.6(2)
			1.993(6)	
			1.996(4)	
	1.995(6)			
			2.238(4)	
Cu2	2.032(5)	96.6(2)	1.981(5)	86.0(2), 92.2(2), 174.0(2), 94.2(2), 176.7(2), 94.4(2), 89.3(2), 90.4(2)
			2.222(6)	
	2.023(6)		2.001(5)	
			2.290(5)	
Cu3	2.075(9)	93.5(3)	1.983(9)	89.4(4), 95.6(3), 91.1(3), 175.4, 175.1(3), 89.7(3), 94.7(2), 88.2(3)
			2.206(7)	
	2.005(7)		2.231(5)	
			2.017(8)	

In coordinated ligand 5 (Fig. 2), the three tpy domains are crystallographically independent, and coordination occurs only through the two outer nitrogen atoms. The tpy unit containing N1, N2 and N3 and N4, N5 and N6 display a coordination directionality close to conformation A (Scheme 1), with N1 and N6 pointing out and N4 and N5 oriented towards the centre core of 5. In contrast, the tpy with N7, N8 and N9 adopts conformation C (Scheme 1). Angles between the least squares planes through pairs of adjacent pyridine rings are in the range 7.6–30.7° (Table 2). The larger torsion angles of 60.0, 84.2 and 56.7° between the planes of the arene core and the three arene spacers (Table 2) minimize unfavourable Me⋯H repulsions. Similar twists occur between the arene spacers and the central pyridine of the tpy substituents, although to a lesser extent (35.6, 30.5 and 53.4°), reducing the H⋯H inter-ring repulsions.

**Table 2:** Angles between the planes of pairs of connected rings in coordinated ligand 5

Tpy unit	Angle between planes of connected pyridine rings/°	Angle between the central pyridine ring and arene spacer/°	Angle between arene spacer and arene core/°
N1N2N3	7.6, 22.6	35.6	60.0
N4N5N6	17.0, 19.1	30.5	84.2
N7N8N9	30.7, 24.9	53.4	56.7

**Fig. 4:**
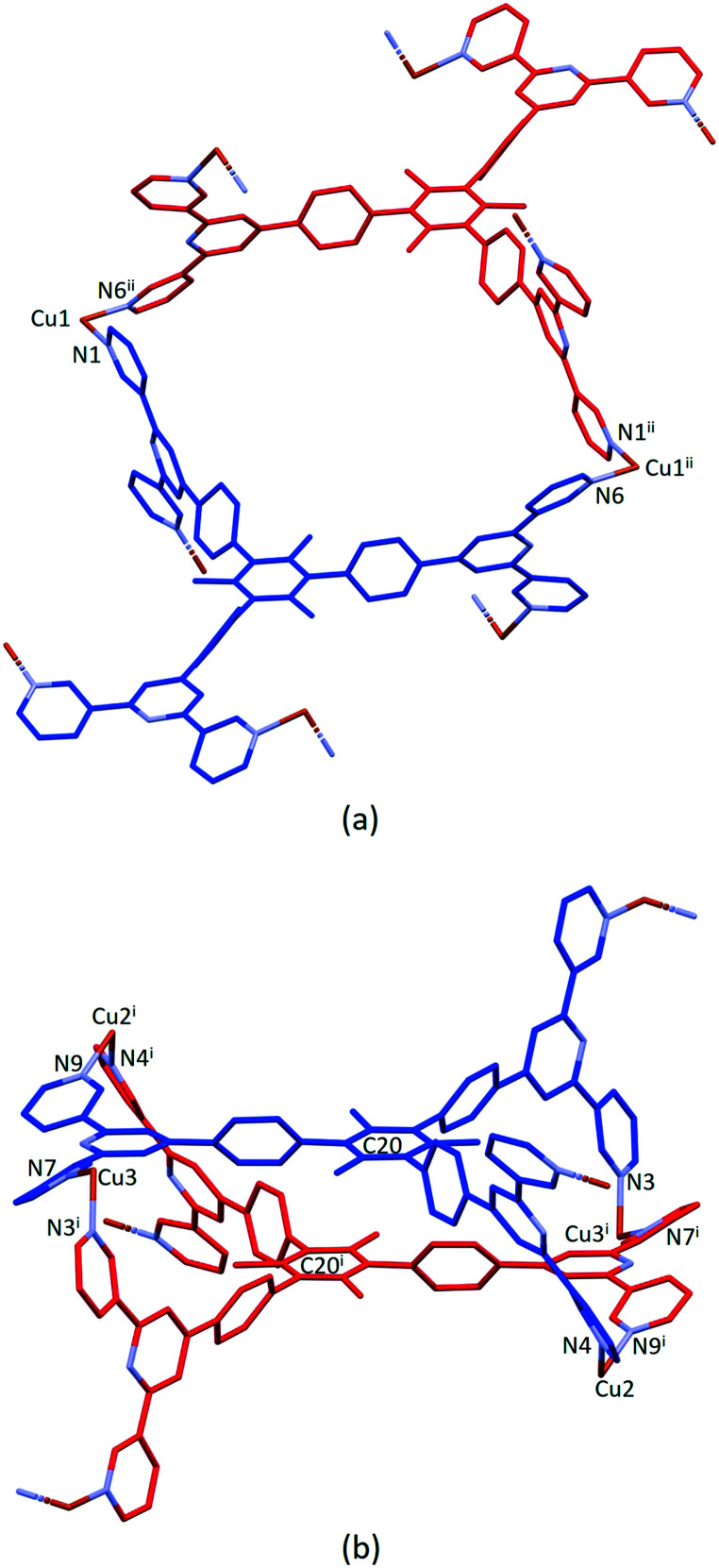
(a) Metallomacrocyclic unit consisting of 5 (blue), Cu1 and the symmetrical equivalents 5^ii^ (red) and Cu1^ii^. (b) The second motif in the 1D-polymer with symmetry generated copper centres and ligand 5^i^ (red). For clarity, H atoms, coordinated [hfacac]^−^ ligands and solvent are omitted, and only major occupancies are shown. Symmetry codes: i = 1 − *x*, 1 − *y*, 1 − *z*; ii = 1 − *x*, −*y*, 1 − *z*.

Ligand 5 presents a 6-connecting building block (Fig. 2) and, combined with the *cis*-arrangement of the pyridine donors in the Cu(ii) coordination sphere, this leads, rather unexpectedly, to a 1D-coordination polymer. There are two building blocks which alternate along the chain. The first is the centrosymmetric metallomacrocyclic unit in Fig. 4a in which Cu1 and Cu1^ii^ bridge pairs of 3,2′:6′,3′′-tpy domains bound through atoms N1 and N6^ii^, and N6 and N1^ii^ (symmetry code ii = 1 − *x*, −*y*, 1 − *z*).

**Fig. 5:**
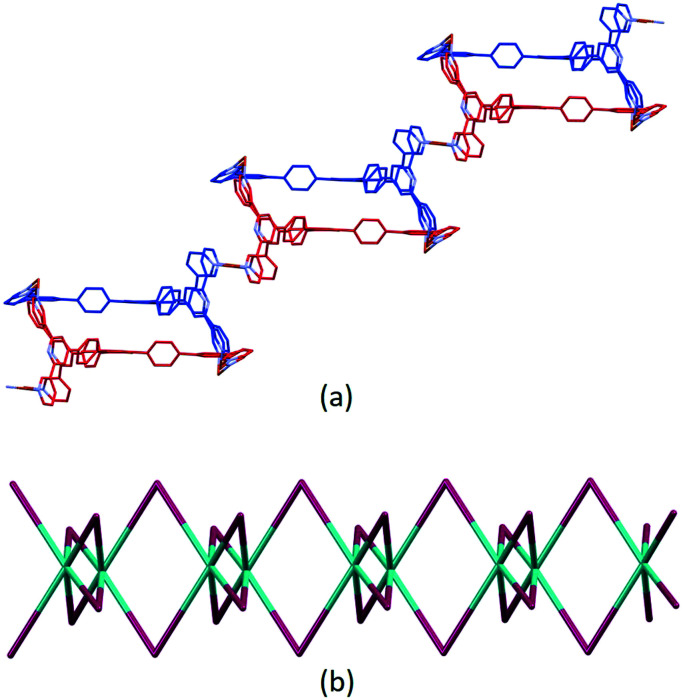
(a) Part of the 1D-coordination polymer. Tristpy building blocks are alternating coloured in blue and red. For clarity, H atoms, coordinated [hfacac]^−^ ligands and solvent are omitted, and only major occupancies are shown. (b) Loops forming the 1D-chain. Magenta: Cu; green: ligand centroids.

The second motif in the 1D-polymer consists of a head-to-tail pairs of two ligands 5 (Fig. 4b). The two tpy ligands are coordinated with Cu2, Cu2^i^, Cu3 and Cu3^i^ across an inversion centre (symmetry code i = 1 − *x*, 1 − *y*, 1 − *z*). All inwardly oriented nitrogen atoms of 5, which come from all three tpy domains, are involved in the building block. The tpy unit containing N7, N8 and N9 is connected to both the tpy unit with N1^i^, N2^i^ and N3^i^ and that with N4^i^, N5^i^ and N6^i^ with atoms Cu3 and Cu2^i^ acting as linkers (Fig. 4b, symmetry code i = 1 − *x*, 1 − *y*, 1 − *z*). Interestingly, there are no π-stacking interactions within the motif, even though the arene cores are coplanar. Centroid⋯centroid separations between for pairs of rings containing C20/C20^i^ is 5.00 Å (Fig. S45†), and it is clear that the steric hindrance caused by the methyl substituents prevents π-interactions. The combination of the two structural motifs in the 1D-polymer (Fig. 5a) leads to a series of connected loops with ligand 5 connecting pairs of Cu atoms (Fig. 5). Atoms Cu1 and Cu1^ii^ lie within a single loop in Fig. 5b, while Cu2, Cu3, Cu2^i^ and Cu3^i^ lie within the double loops in Fig. 5b.

**Fig. 6:**
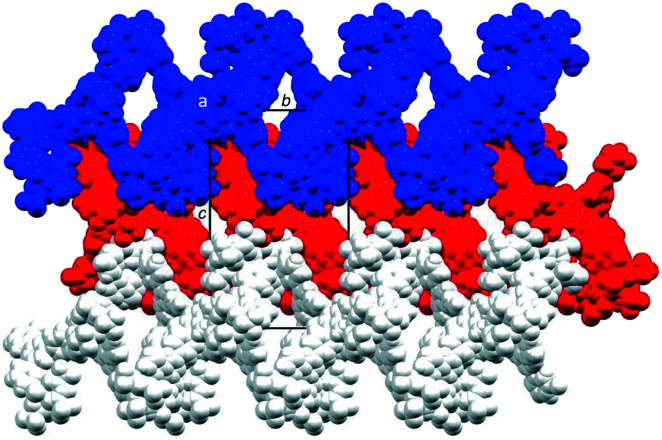
Packing of three 1D ribbons which follow the *b*-axis, shown in blue, red and white, and viewed down the crystallographic *a*-axis. For clarity, solvent is omitted and only major occupancies are shown.

The coordination polymer chains follow the crystallographic *b*-axis and packing interactions between the chains is dominated by short C–H⋯F–C and C–F⋯F–C contacts. However, the disordering of some CF_3_ groups means that detailed discussion is not meaningful. The nesting of the CP chains shown in Fig. 6 illustrates a zigzag arrangement with the chains slightly offset (Fig. S46†). Channels in the structure are occupied by highly disordered toluene and CHCl_3_ molecules.

### PXRD analysis

**Fig. 7:**
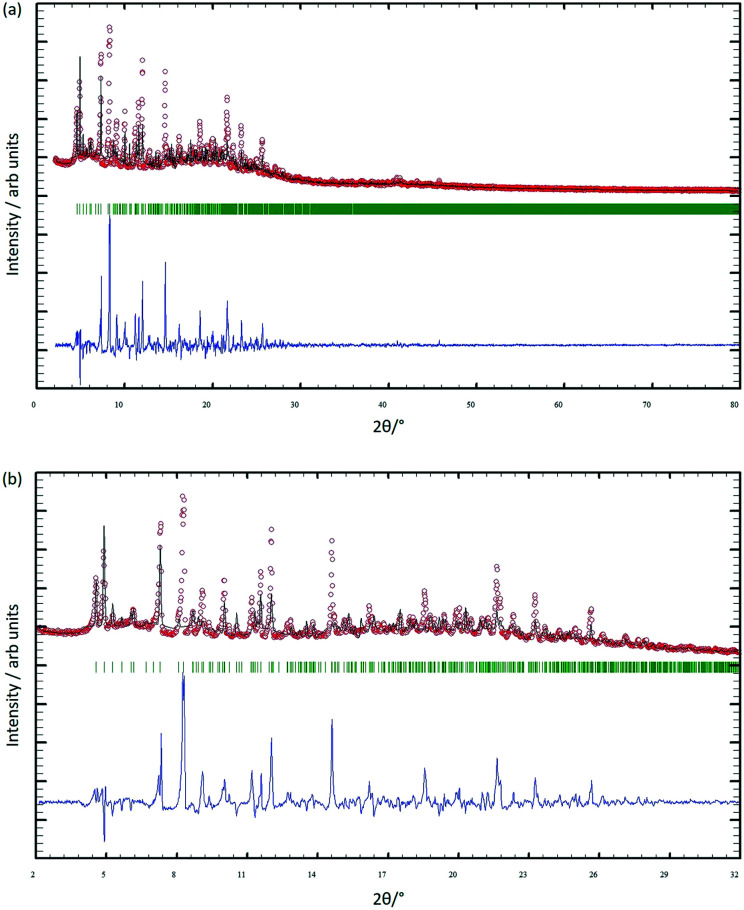
(a) X-ray diffraction (CuKα1 radiation) pattern (red circles) of the bulk crystalline material of [Cu_3_(hfacac)_6_(5)]_*n*_·2.8*n*C_7_H_8_·0.4*n*CHCl_3_, fitting the predicted pattern from the single crystal structure. The black lines are the best fit from Rietveld refinements, and green lines display the Bragg peak positions. The blue plot gives the difference between calculated and experimental points (see text). (b) Expansion in the 2–32° range.

After four single crystals of [Cu_3_(hfacac)_6_(5)]_*n*_·2.8*n*C_7_H_8_·0.4*n*CHCl_3_ had been selected for single-crystal X-ray structure determination, the bulk material was analysed by powder X-ray diffraction (PXRD). The crystals lose solvent rapidly on exposure to air and were, therefore, analysed wet and without washing them. Despite careful handling of the crystals, loss of solvent could not be avoided, and as a consequence, the PXRD spectrum was of poor quality. Confirmation that the single crystal selected was representative of the main phase of the bulk sample came from a comparison of the experimental PXRD pattern (shown in red in Fig. 7) with the pattern predicted from the single crystal structure (black traces in Fig. 7). Minor phases observed in the PXRD spectrum of the bulk material could not be assigned and most likely appear as a consequence of solvent loss during sample preparation and measurement. The differences in intensities (blue traces in Fig. 7) can be justified in terms of differences in the preferred orientations of the crystallites in the bulk powder samples.

### Comments on 1D-coordination polymers containing terpyridine metal-binding domains

**Scheme 5:**
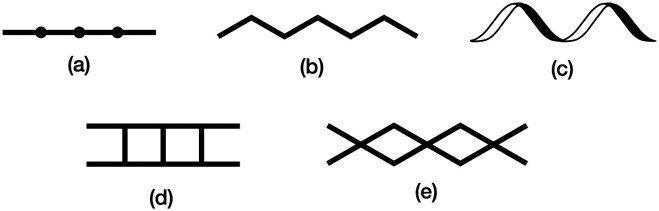
Examples of structure types found in 1D-coordination polymers: (a) linear chain, (b) zigzag chain, (c) helical chain, (d) ladder, (e) series of loops with 4-connecting nodes.

The structure of the 1D-chain in [Cu_3_(hfacac)_6_(5)]_*n*_ consists of alternating single and double loops (Fig. 5b) with this motif being a consequence of the 6-connecting node presented by ligand 5. The use of the term ‘node’ in a 1D-chain has been described as ‘subjective’,^1^ since the chain can be described unambiguously in terms of a combination of metal linkers and ligand linkers. Topologically, the 1D-chains in Scheme 5 are all equivalent, but they are structurally distinct in terms of the organization of the chemical building blocks, with, for example, Scheme 5d and e having enclosed space within the 1D-chain.

**Fig. 8:**
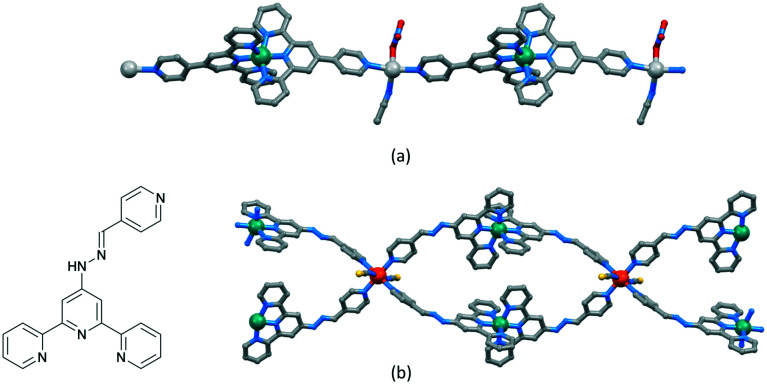
(a) Part of the linear 1D-chain in [{Ru(pytpy)_2_Ag(NCMe)(NO_3_)}_*n*_][NO_3_]_2*n*_·*n*H_2_O·*n*MeCN (CSD refcode WICSIL). (b) Structure of ligand 6 and part of the chain comprising interconnected loops in [{Fe(NCS)_2_(Ru(6)_2_)_2_}_*n*_][Fe_2_(NCS)_6_(OEt)_2_(EtOH)_2_]_*n*_[NCS]_2*n*_·4*n*EtOH·*n*H_2_O (CSD refcode TOPLIU).

The versatility of terpyridine as a building block for 1D-CPs is worthy of comment. With a 2,2′:6′,2′′-tpy metal-binding domain, {M(tpy)_2_}^*n*+^ units can be functionalized in the 4′-position to provide a linear building block. An example is [Ru(pytpy)_2_]^2+^ (pytpy = 4′-(4-pyridyl)-2,2′:6′,2′′-terpyridine), the [PF_6_]^−^ salt of which reacts with AgNO_3_ in aqueous MeCN solution to give [{Ru(pytpy)_2_Ag(NCMe)(NO_3_)}_*n*_][NO_3_]_2*n*_·*n*H_2_O·*n*MeCN containing the 1D-chain shown in Fig. 8a.^69^ The linearity (Scheme 5a and Fig. 8a) of the chain is a consequence of the vectorial properties of the [Ru(pytpy)_2_]^2+^ unit coupled with the N–Ag–N angle of 150.4(1)°. In contrast, ongoing from pytpy to 4-pyridinecarbaldehyde 4′-(2,2′:6′,2′′-terpyridyl)hydrazone (6, Fig. 8b), a significant degree of curvature is introduced into the [Ru(6)_2_]^2+^ unit compared to [Ru(pytpy)_2_]^2+^. This renders the [Ru(6)_2_]^2+^ domain amenable to the formation of 1D-chains comprising a string of loops as seen in [{Fe(NCS)_2_(Ru(6)_2_)_2_}_*n*_]^4*n*+^ (Fig. 8b).^70^

**Fig. 9:**
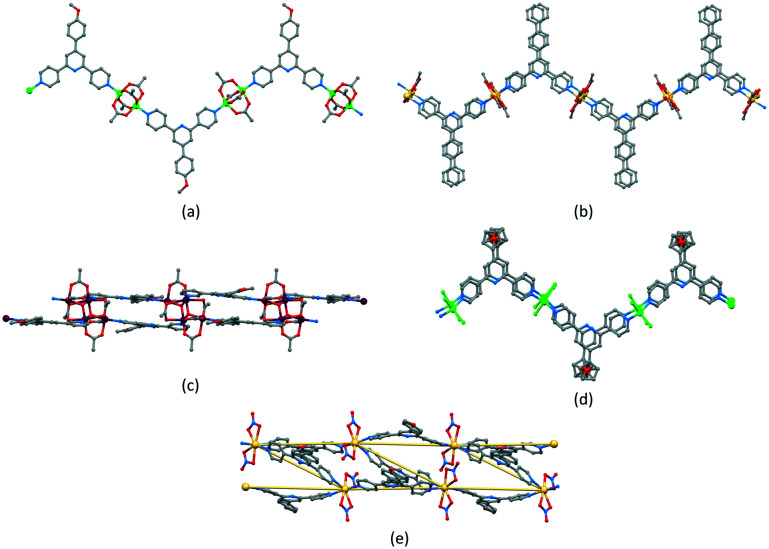
(a) Part of the zigzag chain in [Zn_2_(OAc)_4_(4′-(4-MeOC_6_H_4_)-4,2′:6′,4′′-tpy)]_*n*_ (CSD refcode SOXSEF). Parts of the double stranded zigzag chains in (b) [Cd_2_(OAc)_4_(4′-(4-PhC_6_H_4_)-4,2′:6′,4′′-tpy)]_*n*_ (refcode RIGJEY), (c) [Cu_4_(μ_3_-OH)_2_(OAc)_6_(4′-(4-MeOC_6_H_4_)-3,2′:6′,3′′-tpy)_2_]_*n*_ (refcode KUCHIC), and (d) [Zn_2_Cl_2_(1,1′-bis(4,2′:6′,4′′-terpyridin-4′-yl)ferrocene)]_*n*_ (refcode UMUYUY). (e) Part of the ladder assembly in [Cd_2_(4′-(4-MeOC_6_H_4_)-4,2′:6′,4′′-tpy)_3_(NO_3_)_4_]_*n*_ (refcode BUDZIL); the Cd⋯Cd vectors which define the ladder are highlighted.

Zigzag chains (Scheme 5b) directed by metal paddle-wheel domains such as {Zn_2_(μ-O_2_CMe)_4_} and {Cu_2_(μ-O_2_CMe)_4_} are well represented for a wide range of 4′-substituted 4,2′:6′,4′′-tpy ligands (*e.g.*Fig. 9a^71^) and the conformational flexibility of the 3,2′:6′,3′′-tpy domain leads to structural variants on the zigzag backbone.^72^ Multiply-stranded chains^18^ which retain the zigzag profile arise when multinuclear metal units can bind to more than two ligands, *e.g.* [Cd_2_(OAc)_4_(4′-(4-PhC_6_H_4_)-4,2′:6′,4′′-tpy)]_*n*_ in which each {Cd_2_(OAc)_4_} (a non-paddle wheel motif) binds to four N donors (Fig. 9b^73^), and [Cu_4_(μ_3_-OH)_2_(OAc)_6_(4′-(4-MeOC_6_H_4_)-3,2′:6′,3′′-tpy)_2_]_*n*_ (Fig. 9c^74^). In contrast, the rotational freedom of the ferrocenyl core in the tetratopic ligand 1,1′-bis(4,2′:6′,4′′-terpyridin-4′-yl)ferrocene allows the assembly of a double-stranded chain in which the ligand ‘folds over’ to produce the two strands (Fig. 9d^75^). Helical chains are also common. Many are based upon tetrahedral {Zn_2_X_2_N_2_} domains, and are exemplified by [ZnCl_2_(4,2′:6′,4′′-tpy)]_*n*_ which was the first CP reported which was directed by a 4,2′:6′,4′′-tpy ligand.^76^ Ladders (Scheme 5d) in which the uprights and rungs of the ladder are both defined by ligand linkers are less common, and one example is [Cd_2_(4′-(4-MeOC_6_H_4_)-4,2′:6′,4′′-tpy)_3_(NO_3_)_4_]_*n*_ (Fig. 9e^77^).

The short survey above is not comprehensive, but illustrates the diversity of 1D-CPs that can be assembled using ligands with 2,2′:6′,2′′-, 3,2′:6′,3′′- and 4,2′:6′,4′′-tpy metal-binding domains. To the best of our knowledge, the looped-structure of the chain in the solvates of [Cu_3_(hfacac)_6_(5)]_*n*_ presented here is unique among terpyridine-based complexes.

## Conclusions

We have prepared and characterised five new 1–5 tris(terpyridine) ligands, two with 4,2′:6′,4′′-tpy units and three with 3,2′:6′,3′′-tpy metal-binding domains. The crystal structure of 1 was determined. It possesses a propeller-shaped structure and efficient face-to-face π-stacking interactions predominate in the packing. Reaction of 5 with [Cu(hfacac)_2_]·H_2_O under ambient conditions of crystal growth by layering using a combination of CHCl_3_ and toluene unexpectedly led to the assembly of the 1D-coordination polymer [Cu_3_(hfacac)_6_(5)]_*n*_·2.8*n*C_7_H_8_·0.4*n*CHCl_3_. This possesses an unusual 1D-chain consisting of a series of alternating single and double loops in which the {Cu(hfacac)_2_(N_1_)(N_2_)} units display a *cis*-arrangement of N atoms. In coordinated ligand 5, two of the crystallographically independent 3,2′:6′,3′′-tpy domains adopt conformation A (Scheme 1), while the third exhibits conformation C. PXRD confirms that the single crystal structure is representative of the bulk material. A preliminary structural analysis has also shown that a switch from toluene to 1,2-dichlorobenzene leads to a reproducible assembly of the 1D-looped-chain. We will now further explore the effects of using different spacers in the hexatopic motifs of tris(4,2′:6′,4′′-tpy) and tris(3,2′:6′,3′′-tpy) building blocks.

## Experimental

### Materials and instrumentation

1,3,5-Tribromobenzene and benzene-1,3,5-tricarbaldehyde were purchased from Acros Organics. 3-Acetylpyridine, 4-acetylpyridine and [Pd(PPh_3_)_4_] were bought from Sigma Aldrich and [Cu(hfacac)_2_]·H_2_O was purchased from abcr GmbH. Bis(pinacolato)diboron and Pd(dppf)Cl_2_ were purchased from Fluorochem. All chemicals were used as received.

4′-(4-Bromophenyl)-3,2′:6′,3′′-terpyridine and 4′-(4-bromophenyl)-4,2′:6′,4′′-terpyridine were prepared according to the one-pot methods of Hanan^23^ as reported by Zhang^78^ but using KOH in place of NaOH. 2,4,6-Tribromomesitylene was synthesized as previously described in literature.^79^

Analytical thin-layer chromatography was conducted with pre-coated silica gel 60 *F*_254_ or with aluminium oxide 60 *F*_254_, neutral, aluminium sheets and visualized using ultraviolet light (254 nm). Neutral aluminium oxide 60 *F*_254_ (20 × 20 cm; 1.5 mm) sheets were used for the preparative layer chromatography (PLC). Flash column chromatography was performed on a Biotage Selekt system with self-packed silica gel columns (SiliaFlash® P60, 40–63 μm, 230 400 mesh from SiliCycle Inc.) or using Biotage Sfär Silica HC D columns (50 g, 20 μm).

^1^H and ^13^C{^1^H} NMR spectra were recorded on a Bruker Avance III-500 spectrometer at 298 K. The ^1^H and ^13^C NMR chemical shifts were referenced with respect to the residual solvent peak (*δ* TMS = 0). MALDI-TOF mass spectra were recorded on a Shimadzu MALDI 8020 without matrix, in some cases using α-cyano-4-hydroxycinnamic acid as matrix. High-resolution electrospray (HR-ESI) mass spectra were measured on a Bruker maXis 4G QTOF instrument. PerkinElmer UATR Two and Cary-5000 instruments were used to record FT-infrared (IR) and UV-vis absorption spectra, respectively. Melting temperatures were determined using a Stuart melting point SMP 30 device. Reactions and procedures under microwave conditions were carried out in a Biotage Initiator 8 reactor. Microwave vials were from Biotage and were selected depending on the required solvent volume. For centrifugation an Eppendorf Centrifuge 5415 R was used with 2, 10 and 50 mL samples.

### Synthesis

#### 1,3,5-Tris(4,2′:6′,4′′-terpyridin-4′-yl)benzene (1)

Benzene-1,3,5-tricarbaldehyde (300 mg, 1.81 mmol) was dissolved in 25 mL EtOH, then 4-acetylpyridine (1.51 mL, 13.6 mmol) was added to the solution and crushed KOH (762 mg, 13.6 mmol) was added in one portion. NH_3_ solution (32%, 17 mL) was slowly added and the reaction mixture was stirred at room temperature for 3 days. The solid that formed was collected by centrifugation, washed with water (3 × 50 mL), ethanol (2 × 50 mL) and diethyl ether (2 × 50 mL), and then dried *in vacuo* overnight. The product was recrystallized from 40 mL hot DMF (cooled down to 5 °C). Pale yellow crystals were isolated by filtration, washed with ethanol and then dried *in vacuo* for 24 hours. 1·1.5DMF (341 mg, 0.39 mmol, 22%). M.p. > 390 °C. ^1^H NMR (500 MHz, DMSO-d_6_) *δ*/ppm 8.81 (m, 12H, H^A2^), 8.80 (s, 3H, H^C2^), 8.77 (s, 6H, H^B3^), 8.41 (d, *J* = 6.16 Hz, 12H, H^A3^). ^13^C{^1^H} NMR (126 MHz, DMSO-d_6_) *δ*/ppm 154.5 (C^B2^), 150.4 (C^A2^), 150.1 (C^B4^), 145.4 (C^A4^), 139.3 (C^C1^), 128.1 (C^C2^), 121.4 (C^A3^), 120.0 (C^B3^). UV-vis (DMF, 1.0 × 10^−5^ mol dm^−3^) *λ*/nm 306 (*ε*/dm^3^ mol^−1^ cm^−1^ 30 600). MALDI-TOF-MS *m*/*z* 772.03 [M + H]^+^ (calc. 772.29). Found C 75.92, H 4.85, N 16.56; required for C_51_H_33_N_9_·1.5DMF: C 75.62, H 4.97, N 16.68.

#### 1,3,5-Tris(3,2′:6′,3′′-terpyridin-4′-yl)benzene (2)

Benzene-1,3,5-tricarbaldehyde (300 mg, 1.81 mmol) was dissolved in 25 mL EtOH, then 3-acetylpyridine (1.49 mL, 13.6 mmol) was added to the solution and crushed KOH (762 mg, 13.6 mmol) was added in one portion. NH_3_ solution (32%, 17 mL) was slowly added and the reaction mixture was stirred at room temperature for 3 days. The solid that formed was collected by centrifugation, washed with water (3 × 50 mL), ethanol (3 × 30 mL) and then dried *in vacuo*. The product was dissolved in the minimum amount of CHCl_3_, then 100 mL Et_2_O were added and the mixture was cooled down to −20 °C. After one day a colourless precipitate was isolated by cold filtration, washed 5 mL Et_2_O and then dried *in vacuo* for 24 hours (172 mg, 0.22 mmol, 12.3%). M.p. > 390 °C. ^1^H NMR (500 MHz, DMSO-d_6_): *δ*/ppm 9.61 (dd, *J* = 2.3, 0.9 H, 6H, H^A2^), 8.79 (s, 3H, H^C2^), 8.77 (ddd, *J* = 8.0, 2.4, 1.7 H, 6H, H^A4^), 8.72 (dd, *J* = 4.7, 1.7 Hz, 6H, H^A6^), 8.68 (s, 6H, H^B3^), 7.62 (ddd, *J* = 8.0, 4.7, 0.9 Hz, 6H, H^A5^). ^13^C{^1^H} NMR (126 MHz, DMSO-d_6_) *δ*/ppm 154.8 (C^B2^), 150.2 (C^A6^), 149.6 (C^B4^), 148.5 (C^A2^), 139.4 (C^C1^), 134.6 (C^A4^), 134.0 (C^A3^), 127.8 (C^C2^), 123.8 (C^A5^), 118.5 (C^B3^). UV-vis (DMF, 1.0 × 10^−5^ mol dm^−3^) *λ*/nm 312 (*ε*/dm^3^ mol^−1^ cm^−1^ 25 700). MALDI-TOF-MS *m*/*z* 772.33 [M + H]^+^ (calc. 772.29). Found C 79.02, H 4.31, N 16.58; required for C_51_H_33_N_9_: C 79.36, H 4.31, N 16.33.

#### 4′-(4-Pinacolatoboronphenyl)-4,2′:6′,4′′-terpyridine (**P1**)

A 20 mL microwave vial was charged with Pd(dppf)Cl_2_ (69.6 mg, 8.52 × 10^−2^ mmol), AcOK (836 mg, 8.52 mmol) and bis(pinacolato)diboron (B_2_pin_2_ 762 mg, 2.97 mmol) and flushed with nitrogen. Degassed DMF (14 mL) and 4′-(4-bromophenyl)-4,2′:6′,4′′-terpyridine (1099 mg, 2.83 mmol) were then added. The mixture was stirred and heated at the microwave at 120 °C for 5 h 30 min under nitrogen. Toluene (100 mL) was added to the product and the toluene–DMF mixture washed with water (3 × 100 mL). The toluene layer was dried over MgSO_4_ and filtered. The solvent was removed from the filtrate by rotary evaporation to give a pale yellow solid. The residue was dissolved the minimum amount of hot CHCl_3_/hexane (1 : 1) and then precipitated at −20 °C to give a colourless solid. The product was isolated by filtration, washed with EtOH and hexane and then dried *in vacuo*. The filtrate was dried and the yellow residue purified by column chromatography (50 g Biotage Sfär Silica HC D column, Biotage Select, eluent: 30% acetone–70% cyclohexane) to yield a second crop of product (661 mg, 1.52 mmol, 53.7%). M.p. = 229.8–231.5 °C. ^1^H NMR (500 MHz, CDCl_3_): *δ*/ppm 8.80 (d, *J* = 6.1 Hz, 4H, H^A2^), 8.09 (d, *J* = 6.2 Hz, 4H, H^A3^), 8.06 (s, 2H, H^B3^), 8.00 (d, *J* = 8.1 Hz, 2H, H^C3^), 7.75 (d, *J* = 8.2 Hz, 2H, H^C2^), 1.39 (s, 12 H, H^b^). ^13^C{^1^H} NMR (126 MHz, CDCl_3_) *δ*/ppm 155.5 (C^B2^), 151.2 (C^B4^), 150.7 (C^A2^), 146.2 (C^A4^), 140.6 (C^C1^), 135.9 (C^C3^), 130.5 (C^C4^), 126.6 (C^C2^), 121.4 (C^A3^), 119.2 (C^B3^), 84.3 (C^a^), 25.1 (C^b^). UV-vis (CHCl_3_, 2.6 × 10^−5^ mol dm^−3^) *λ*/nm 264 (*ε*/dm^3^ mol^−1^ cm^−1^ 45 466). MALDI-TOF-MS *m*/*z* 436.19 [M + H]^+^ (calc. 436.22). Found C 73.68, H 6.08, N 9.83; required for C_27_H_26_N_3_O_2_B: C 74.49, H 6.02, N 9.65.

#### 4′-(4-Pinacolatoboronphenyl)-3,2′:6′,3′′-terpyridine (**P2**)

A 20 mL microwave vial was charged with Pd(dppf)Cl_2_ (69.8 mg, 8.55 × 10^−2^ mmol), AcOK (839 mg, 8.55 mmol) and B_2_pin_2_ (765 mg, 2.98 mmol) and flushed with nitrogen. Degassed DMF (14 mL) and 4′-(4-bromophenyl)-3,2′:6′,3′′-terpyridine (1103 mg, 2.98 mmol) were then added. The mixture was stirred and heated at the microwave at 120 °C for 5 h and 30 min under nitrogen. CHCl_3_ (100 mL) was added to the product and the CHCl_3_–DMF mixture washed with water (3 × 100 mL). The CHCl_3_ layer was dried over MgSO_4_ and filtered. The solvent was removed by rotary evaporation to give a brown residue. Purification by column chromatography (16 × 4 cm self-packed silica gel column, Biotage Select, eluent: 80% EtOAc – 20% cyclohexane) gave the desired product (516 g, 1.19 mmol, 41.7%) as a colourless solid. M.p. = 187.0–187.2 °C. ^1^H NMR (500 MHz, CDCl_3_): *δ*/ppm 9.39 (dd, *J* = 2.3, 0.9 Hz, 2H, H^A2^), 8.71 (dd, *J* = 4.8, 1.6 Hz, 2H, H^A6^), 8.51 (ddd, *J* = 7.9, 2.3, 1.7 Hz, 2H, H^A4^), 7.99 (d, *J* = 8.2 Hz, 2H, H^C3^), 7.97 (s, 2H, H^B3^), 7.76 (d, *J* = 8.1 Hz, 2H, H^C2^), 7.47 (ddd, *J* = 7.9, 4.8, 0.9 Hz, 2H, H^A5^), 1.39 (s, 12 H, H^b^). ^13^C{^1^H} NMR (126 MHz, CDCl_3_) *δ*/ppm 155.6 (C^B2^), 150.9 (C^B4^), 150.4 (C^A6^), 148.7 (C^A2^), 140.9 (C^C1^), 135.8 (C^C3^), 134.8 (C^A3^), 134.7 (C^A4^), 130.2 (C^C4^), 126.6 (C^C2^), 123.8 (C^A5^), 118.0 (C^B3^), 84.3 (C^a^), 25.1 (C^b^). UV-vis (CHCl_3_, 2.6 × 10^−5^ mol dm^−3^) *λ*/nm 263 (*ε*/dm^3^ mol^−1^ cm^−1^ 41 712). MALDI-TOF-MS *m*/*z* 436.16 [M + H]^+^ (calc. 436.22). Found C 74.08, H 5.88, N 9.52; required for C_27_H_26_N_3_O_2_B: C 74.49, H 6.02, N 9.65.

#### 1,3,5-Tris{4-(4,2′:6′,4′′-terpyridin-4′-yl)phenyl}benzene (3)

In a Schlenk tube, a mixture of 4′-(4-pinacolatoboronphenyl)-4,2′:6′,4′′-terpyridine (634 mg, 1.46 mmol), 1,3,5-tribromobenzene (115 mg, 0.364 mmol), Pd(PPh_3_)_4_ (42 mg 0.036 mmol), 0.2 M aqueous Na_2_CO_3_ (10.9 mL, 2.18 mmol) and degassed THF (53 mL) was refluxed for 12 h under nitrogen. The off-white precipitate was isolated by filtration, washed with H_2_O, THF and Et_2_O. The white solid was dried under vacuum at 80 °C up to constant weight (137 mg, 0.137 mmol, 37.6%). M.p. > 390 °C. ^1^H NMR (500 MHz, DMSO-d_6_ + TFA-d) *δ*/ppm 9.15 (d, *J* = 6.1 Hz, 12H, H^A2^), 9.11 (d, *J* = 6.2 Hz, 12H, H^A3^), 8.98 (s, 6H, H^B3^), 8.35 (d, *J* = 7.9 Hz, 6H, H^C2^), 8.17 (m, 9H, H^C3+D2^). ^13^C{^1^H} NMR (126 MHz, DMSO-d_6_ + TFA-d) *δ*/ppm 154.3 (C^A4^), 152.6 (C^B2^), 151.8 (C^B4^), 143.1 (C^A2^), 142.7 (C^C4^), 141.9 (C^D1^), 136.1 (C^C1^), 129.1 (C^C2^), 128.8 (C^C3^), 126.1 (C^D2^), 125.2 (C^A3^), 123.3 (C^B3^). UV-vis (benzonitrile, 8.6 × 10^−6^ mol dm^−3^) *λ*/nm 307 (*ε*/dm^3^ mol^−1^ cm^−1^ 88 183). MALDI-TOF-MS *m*/*z* 1000.30 [M + H]^+^ (calc. 1000.39). HR-ESI MS *m*/*z* 1000.3860 [M + H]^+^ (calc. 1000.3871).

#### 1,3,5-Tris{4-(3,2′:6′,3′′-terpyridin-4′-yl)phenyl}benzene (4)

In a Schlenk tube, a mixture of 4′-(4-pinacolatoboronphenyl)-3,2′:6′,3′′-terpyridine (675 mg, 1.55 mmol), 1,3,5-tribromobenzene (122 mg, 0.388 mmol), Pd(PPh_3_)_4_ (45 mg 0.039 mmol), 0.2 M aqueous Na_2_CO_3_ (11.6 mL, 2.33 mmol) and degassed THF (56 mL) was refluxed for 12 h under nitrogen. The off-white precipitate was isolated by filtration, washed with H_2_O, THF and Et_2_O. The off-white solid was dried under vacuum at 80 °C up to constant weight (313 mg, 0.313 mmol, 80.7%). M.p. = over 390 °C. ^1^H NMR (500 MHz, DMF-d_7_): *δ*/ppm 9.68 (dd, *J* = 2.4, 0.9 Hz, 6H, H^A2^), 8.86 (ddd, *J* = 8.1, 2.4, 1.7 Hz, 6H, H^A4^), 8.77 (dd, *J* = 4.7, 1.6 Hz, 6H, H^A6^), 8.62 (s, 6H, H^B3^), 8.43 (d, *J* = 8.5 Hz, 6H, H^C2^), 8.34 (s, 3H, H^D2^), 8.31 (d, *J* = 8.5 Hz, 6H, H^C3^), 7.66 (ddd, *J* = 7.9, 4.7, 0.9 Hz, 6H, H^A5^). ^13^C{^1^H} NMR (126 MHz, DMF-d_7_) *δ*/ppm 155.6 (C^B2^), 150.7 (C^A6^), 150.3 (C^B4^), 149.0 (C^A2^), 141.9 (C^C4+D1^), 137.4 (C^C1^), 134.8 (C^A3+A4^), 128.5 (C^C2^), 128.5 (C^C3^), 125.5 (C^D2^), 124.1 (C^A5^), 117.9 (C^B3^). UV-vis (DMF, 8.0 × 10^−6^ mol dm^−3^) *λ*/nm 305 (*ε*/dm^3^ mol^−1^ cm^−1^ 108 675). MALDI-TOF-MS *m*/*z* 1000.41 [M + H]^+^ (calc. 1000.39). HR-ESI MS *m*/*z* 1000.3859 [M + H]^+^ (calc. 1000.3871).

#### 1,3,5-Trimethyl-2,4,6-tris{4-(3,2′:6′,3′′-terpyridin-4′-yl)phenyl}benzene (5)

In a Schlenk tube, a mixture of 4′-(4-pinacolatoboronphenyl)-3,2′:6′,3′′-terpyridine (200 mg, 0.459 mmol), 2,4,6-tribromomesitylene (36.4 mg, 0.102 mmol), Na_2_CO_3_ (162 mg, 1.53 mmol), and a solvent mixture of water (8 mL) and toluene (8 mL) were added. The system was freeze-pump-thawed (3 times), back filled with nitrogen; and then Pd(dppf)Cl_2_ (14.9 mg 0.0204 mmol) was added. The resultant suspension was refluxed for 1 hour under nitrogen. The acqueous layer was extracted with CHCl_3_ (3 × 20 mL). The combined organic phase was dried (MgSO_4_) and concentrated *in vacuo* to give a brown residue. An initial purification was performed by a single PLC (neutral Alox, eluent: CHCl_3_); then the mixture was divided into 4 portions and each was separately purified by PLC under the same conditions. A colourless solid was isolated (34.9 mg, 0.033 mmol, 32.8%). M.p. > 390 °C. ^1^H NMR (500 MHz, DMF-d_7_): *δ*/ppm 9.66 (dd, *J* = 2.3, 0.9 Hz, 6H, H^A2^), 8.84 (ddd, *J* = 8.0, 2.3, 1.6 Hz, 6H, H^A4^), 8.75 (dd, *J* = 4.7, 1.7 Hz, 6H, H^A6^), 8.61 (s, 6H, H^B3^), 8.41 (d, *J* = 8.5 Hz, 6H, H^C2^), 7.64 (ddd, *J* = 8.0, 4.7, 0.9 Hz, 6H, H^A5^), 7.57 (d, *J* = 8.5 Hz, 6H, H^C3^), 1.93 (s, 9H, H^CH3^). ^13^C{^1^H} NMR (126 MHz, DMF-d_7_) *δ*/ppm 155.6 (C^B2^), 150.7 (C^A6^), 150.4 (C^B4^), 148.9 (C^A2^), 143.6 (C^C4^), 139.8 (C^D1^), 136.3 (C^C1^), 134.8 (C^A3+A4^), 132.9 (C^D2^), 130.6 (C^C3^), 128.3 (C^C2^), 124.1 (C^A5^), 117.8 (C^B3^), 19.6 (C^CH3^). UV-vis (CHCl_3_, 8.0 × 10^−6^ mol dm^−3^) *λ*/nm 272 (*ε*/dm^3^ mol^−1^ cm^−1^ 121 400). MALDI-TOF-MS *m*/*z* 1042.48 [M + H]^+^ (calc. 1042.43). HR-ESI MS *m*/*z* 1042.4313 [M + H]^+^ (calc. 1042.4334), 521.7207 [M + 2H]^2+^ (calc. 521.7206).

#### [Cu_3_(hfacac)_6_(5)]_*n*_·2.8*n*C_7_H_8_·0.4*n*CHCl_3_

A toluene (7 mL) solution of [Cu(hfacac)_2_]·H_2_O (7.6 mg, 0.016 mmol) was layered over a CHCl_3_ solution (6 mL) of 5 (5.2 mg, 0.005 mmol) in a crystallization tube (i.d. = 13.6 mm, vol. = 24 mL) initially sealed with a septum; after 10 days a syringe-needle was introduced into the septum opening the tube to the air. The tube was left to stand at room temperature (*ca.* 22 °C) for 10 days. A fine light green suspension was obtained and removed by filtration. The filtrate was left to evaporate in the air in a test tube with a septum pierced with a syringe-needle. Light green block-like crystals visible to the eye were first obtained after three months, and four single crystals were selected for X-ray diffraction after another month. All the remaining crystals were mounted wet in a sample holder (to avoid loss of solvent from the crystals) and analysed by powder X-ray diffraction (PXRD).

In an another attempt, compound 5 (5.2 mg, 0.005 mmol) was dissolved in CHCl_3_ (6 mL), and then a solution of [Cu(hfacac)_2_]·H_2_O (15.3 mg, 0.031 mmol) in toluene (6 mL) was added. A formation of a pale green precipitate was immediately observed. After filtration, the solution was left to evaporate in the air in a test tube with a septum pierced with a syringe-needle. Light green block-like crystals were obtained after 3 months, and cell checks revealed that these were [Cu_3_(hfacac)_6_(5)]_*n*_·2.8*n*C_7_H_8_·0.4*n*CHCl_3_ (cell parameters, *a* = 20.784(6) Å, *b* = 21.270(3) Å, *c* = 34.158(9) Å, *β* = 103.57(2)°).

An additional reaction was carried out to prepare a sample for E.A. and IR spectroscopy. Compound 5 (10.4 mg, 0.010 mmol) was dissolved in CHCl_3_ (6 mL), and then a solution of [Cu(hfacac)_2_]·H_2_O (15.3 mg, 0.031 mmol) in toluene (6 mL) was added. A formation of a pale green precipitate was immediately observed. The solid that formed was collected by centrifugation, washed with CHCl_3_ and toluene, and then dried *in vacuo*. The product was isolated as a pale green powder. Yield for [Cu_3_(hfacac)_6_(5)]_*n*_·0.5*n*C_7_H_8_ (25.0 mg, 0.010 mmol, 99.2%). Found C 50.77, H 2.64, N 5.34; required for C_105.5_H_61_Cu_3_F_36_N_9_O_12_: C 50.26, H 2.44, N 5.00. PXRD showed that the resultant product was amorphous.

#### Second solvate of [Cu_3_(hfacac)_6_(5)]_*n*_

A 1,2-dichlorobenzene (6 mL) solution of [Cu(hfacac)_2_]·H_2_O (7.6 mg, 0.016 mmol) was layered over a CHCl_3_ solution (6 mL) of 5 (5.2 mg, 0.005 mmol) and anthracene (13.4 mg, 0.075 mmol) in a crystallization tube (i.d. = 13.6 mm, vol. = 24 mL) initially sealed with a septum. After 3 months a fine light green suspension had formed and was removed by filtration. The filtrate was left to evaporate in the air in a test tube with a septum pierced with a syringe-needle. Light green block-like crystals visible to the eye were first obtained after 1 year, and a single crystal was selected for X-ray diffraction after another month.

### Crystallography

Single crystal data for 1·1.75DMF were collected either on a STOE StadiVari diffractometer equipped with a Metaljet D2 source (GaKα radiation) and a Pilatus300K detector. For the CPs, a X06DA-PXIII beamline at the Swiss Light Source (Paul Scherrer Institute, 5232 Villigen, Switzerland) with synchrotron radiation (0.72083 Å) and a PILATUS 2 M-F detector were used. For the former, structures were solved using ShelXT v. 2018/2 (ref. 80) and Olex2,^81^ and the model was refined with ShelXL v. 2018/3.^82^ For the latter, data reduction, solution, and refinement used the programs XDS,^83^ Olex2,^81^ and ShelXL 2018/3.^82^ All H atoms were included at geometrically calculated positions and refined using a riding model with *U*_iso_ = 1.2 of the parent atom. Structure analysis used CSD Mercury 2020.2.0.^68^ In [Cu_3_(hfacac)_6_(5)]_*n*_·2.8*n*C_7_H_8_·0.4*n*CHCl_3_ some CF_3_ groups of the polymer and solvent molecules were disordered and were refined isotropically with geometrical restraints. A certain amount of the solvent was removed using the solvent mask procedure, and this was added to the formulae and appropriated numbers. In the structural discussions, only the major (or one of the equal) occupancy sites are considered in each disordered entity. A mask was also used to treat the solvent region in 1; 1.75 DMF molecules were found and added to the formulae and numbers.

PXRD data were collected at room temperature in transmission mode using a Stoe Stadi P diffractometer, equipped with CuKα1 radiation (Ge(111) monochromator) and a DECTRIS MYTHEN 1K detector. Whole-pattern decomposition (profile matching) analysis of the diffraction patterns was done using the package FULLPROF SUITE (v. September 2020)^84,85^ using a previously determined instrument resolution function based on a NIST640d standard. The structural models were derived from the single crystal X-ray diffraction data. Refined parameters in Rietveld were scale factor, zero shift, lattice parameters, Cu and halogen atomic positions, background points, and peaks shapes as a Thompson Cox Hastings pseudo-Voigt function. Preferred orientations as a March–Dollase multi-axial phenomenological model were incorporated into the analysis.

1·1.75DMF C_51_H_33_N_9_·C_5.25_H_12.25_N_1.75_O_1.75_, *M*_r_ = 899.78, yellow block, orthorhombic, *Pbca*, *a* = 20.3991(5) Å, *b* = 15.4857(3) Å, *c* = 30.0422(5) Å, *V* = 9490.2(3) Å^3^, *T* = 150 K, *Z* = 8, *μ*(GaK_α_) = 0.405. Total 59 250 reflections, 9577 unique (*R*_int_ = 0.0486). Refinement of 5670 reflections (541 parameters) with *I* > 2*σ*(*I*) converged at final *R*_1_ = 0.0515 (*R*_1_ all data = 0.0746), w*R*_2_ = 0.1520 (w*R*_2_ all data = 0.1612), *F*(000) = 3776, gof = 1.177. CCDC 2096132.

[Cu_3_(hfacac)_6_(5)]_*n*_·2.8*n*C_7_H_8_·0.4*n*CHCl_3_: C_122_H_80.1_Cl_1.2_Cu_3_F_36_N_9_O_12_, *M*_r_ = 2781.21, green block, monoclinic, *P*2_1_/*n*, *a* = 19.889(4) Å, *b* = 21.178(4) Å, *c* = 34.375(7) Å, *β* = 104.32(3)°, *V* = 14 029(5) Å^3^, *T* = 100 K, *Z* = 4, *μ*(synchrotron) = 0.595. Total 531 703 reflections, 29 721 unique (*R*_int_ = 0.0862). Refinement of 20 415 reflections (1623 parameters) with *I* > 2*σ*(*I*) converged at final *R*_1_ = 0.1912 (*R*_1_ all data = 0.2092), w*R*_2_ = 0.4998 (w*R*_2_ all data = 0.5408), *F*(000) = 5610, gof = 1.177. CCDC 2096133.

## Author contributions

Investigation and methodology, G. M.; crystallography, A. P.; powder diffraction, G. M.; manuscript writing, G. M., C. E. H.; manuscript editing, C. E. H.; E. C. C.; supervision and project administration, C. E. H.; E. C. C.; funding acquisition, C. E. H.

## Conflicts of interest

There are no conflicts to declare.

## Supplementary Material

CE-024-D1CE01531A-s001

CE-024-D1CE01531A-s002

## References

[cit1] Batten S. R. , Neville S. M. and Turner D. R., Coordination Polymers: Design, Analysis and Application, RSC Publishing, Cambridge, 2009

[cit2] Berger J. E. , Kerrn aller Fridrichs=Städtschen Begebenheiten, Manuskript, Berlin, ca.1730 (Berlin, Staatsbibliothek zu Berlin—Preußischer Kulturbesitz, Handschriftenabteilung, Ms. Boruss. quart. 124)

[cit3] Constable E. C. , in Supramolecular Chemistry: From Molecules to Nanomaterials, ed. P. A. Gale and J. W. Steed, Wiley, Chichester, 2012, vol. 6, p. 3073

[cit4] Loukopoulos E., Kostakis G. E. (2018). J. Coord. Chem..

[cit5] Chen J., Shen K., Li Y. (2017). ChemSusChem.

[cit6] Mukherjee S. , Kumar A. and Zaworotko M. J., in Metal-Organic Frameworks (MOFs) for Environmental Applications, ed. S. K. Ghosh, Elsevier, Amsterdam, 2019, pp. 5–61

[cit7] Sumida K., Rogow D. L., Mason J. A., McDonald T. M., Bloch E. D., Herm Z. R., Bae T.-H., Long J. R. (2012). Chem. Rev..

[cit8] Duan J., Jin W., Kitagawa S. (2017). Coord. Chem. Rev..

[cit9] Peng Y., Yang W. (2020). Adv. Mater. Interfaces.

[cit10] Lin R.-B., Xiang S., Xing H., Zhou W., Chen B. (2019). Coord. Chem. Rev..

[cit11] Zhang Y., Cheng X., Jiang X., Urban J. J., Lau C. H., Liu S., Shao L. (2020). Mater. Today.

[cit12] Ye Y., Gong L., Xiang S., Zhang Z., Chen B. (2020). Adv. Mater..

[cit13] Deng J., Wu F., Yu P., Mao L. (2018). Appl. Mater. Today.

[cit14] Liu J.-Q., Luo Z.-D., Pan Y., Singh A. K., Trivedi M., Kumar A. (2020). Coord. Chem. Rev..

[cit15] Elahi S. M., Raizada M., Sahu P. K., Konar S. (2021). Chem. – Eur. J..

[cit16] Constable E. C., Housecroft C. E. (2017). Coord. Chem. Rev..

[cit17] Constable E. C. (2008). Coord. Chem. Rev..

[cit18] Housecroft C. E. (2014). Dalton Trans..

[cit19] Housecroft C. E. (2015). CrystEngComm.

[cit20] Housecroft C. E., Constable E. C. (2019). Chimia.

[cit21] Housecroft C. E., Constable E. C. (2020). Chem. Commun..

[cit22] Housecroft C. E., Constable E. C. (2021). Molecules.

[cit23] Wang J., Hanan G. S. (2005). Synlett.

[cit24] Kröhnke F. (1976). Synthesis.

[cit25] Liu B., Hou L., Wu W.-P., Dou A.-N., Wang Y.-Y. (2015). Dalton Trans..

[cit26] Wu Y., Wu J., Luo Z., Wang J., Li Y., Han Y., Liu J. (2017). RSC Adv..

[cit27] Yuan F., Yuan C.-M., Hu H.-M., Wang T.-T., Zhou C.-S. (2018). J. Solid State Chem..

[cit28] Housecroft C. E., Constable E. C. (2018). J. Inorg. Organomet. Polym. Mater..

[cit29] Yoshida J., Nishikiori S.-I., Yuge H. (2013). J. Coord. Chem..

[cit30] Klein Y. M., Constable E. C., Housecroft C. E., Prescimone A. (2015). CrystEngComm.

[cit31] Manfroni G., Prescimone A., Batten S. R., Klein Y. M., Gawryluk D. J., Constable E. C., Housecroft C. E. (2019). Crystals.

[cit32] Constable E. C., Thompson A. M. W. C. (1992). J. Chem. Soc., Chem. Commun..

[cit33] Chakraborty S., Newkome G. R. (2018). Chem. Soc. Rev..

[cit34] Wild A., Winter A., Schlütter F., Schubert U. S. (2011). Chem. Soc. Rev..

[cit35] Wu T., Yuan J., Song B., Chen Y.-S., Chen M., Xue X., Liu Q., Wang J., Chan Y.-T., Wang P. (2017). Chem. Commun..

[cit36] Lu X., Li X., Cao Y., Schultz A., Wang J.-L., Moorefield C. N., Wesdemiotis C., Cheng S. Z. D., Newkome G. R. (2013). Angew. Chem., Int. Ed..

[cit37] Wang J.-L., Li X., Shreiner C. D., Lu X., Moorefield C. N., Tummalapalli S. R., Medvetz D. A., Panzner M. J., Fronczek F. R., Wesdemiotis C., Newkome G. R. (2012). New J. Chem..

[cit38] Xie T.-Z., Liao S.-Y., Guo K., Lu X., Dong X., Huang M., Moorefield C. N., Cheng S. Z. D., Liu X., Wesdemiotis C., Newkome G. R. (2014). J. Am. Chem. Soc..

[cit39] Chen M., Liu D., Huang J., Li Y., Wang M., Li K., Wang J., Jiang Z., Li X., Wang P. (2019). Inorg. Chem..

[cit40] Wang G., Chen M., Wang J., Jiang Z., Liu D., Lou D., Zhao H., Li K., Li S., Wu T., Jiang Z., Sun X., Wang P. (2020). J. Am. Chem. Soc..

[cit41] Chakraborty S., Endres K. J., Bera R., Wojtas L., Moorefield C. N., Saunders M. J., Das N., Wesdemiotis C., Newkome G. R. (2018). Dalton Trans..

[cit42] Constable E. C., Housecroft C. E., Poleschak I. (1999). Inorg. Chem. Commun..

[cit43] Constable E. C., Eich O., Housecroft C. E. (1999). Inorg. Chem. Commun..

[cit44] Constable E. C., Harverson P. (1999). Polyhedron.

[cit45] Constable E. C., Eich O., Housecroft C. E., Rees D. C. (2000). Inorg. Chim. Acta.

[cit46] Constable E. C., Eich O., Fenske D., Housecroft C. E., Johnston L. A. (2000). Chem. – Eur. J..

[cit47] Sievers T. K., Vergin A., Möhwald H., Kurth D. G. (2007). Langmuir.

[cit48] Bauer T., Schlüter A. D., Sakamoto J. (2010). Synlett.

[cit49] Maeda H., Sakamoto R., Nishihara H. (2017). Coord. Chem. Rev..

[cit50] Kuai Y., Yang T., Yuan F., Dong Y., Song Q., Zhang C., Wong W.-Y. (2021). Dyes Pigm..

[cit51] Roy S., Chakraborty C. (2020). ACS Appl. Mater. Interfaces.

[cit52] Hu C.-W., Sato T., Zhang J., Moriyama S., Higuchi M. (2014). ACS Appl. Mater. Interfaces.

[cit53] Pandey R. K., Hossain M. D., Sato T., Rana U., Moriyama S., Higuchi M. (2015). RSC Adv..

[cit54] Kuai Y., Li W., Dong Y., Wong W.-Y., Yan S., Dai Y., Zhang C. (2019). Dalton Trans..

[cit55] Roy S., Chakraborty C. (2021). Chem. Commun..

[cit56] Liu Y., Sakamoto R., Ho C.-L., Nishihara H., Wong W.-Y. (2019). J. Mater. Chem. C.

[cit57] Takada K., Sakamoto R., Yi S.-T., Katagiri S., Kambe T., Nishihara H. (2015). J. Am. Chem. Soc..

[cit58] Tsukamoto T., Takada K., Sakamoto R., Matsuoka R., Toyoda R., Maeda H., Yagi T., Nishikawa M., Shinjo N., Amano S., Iokawa T., Ishibashi N., Oi T., Kanayama K., Kinugawa R., Koda Y., Komura T., Nakajima S., Fukuyama R., Fuse N., Mizui M., Miyasaki M., Yamashita Y., Yamada K., Zhang W., Han R., Liu W., Tsubomura T., Nishihara H. (2017). J. Am. Chem. Soc..

[cit59] Groom C. R., Bruno I. J., Lightfoot M. P., Ward S. C. (2016). Acta Crystallogr., Sect. B.

[cit60] Bruno I. J., Cole J. C., Edgington P. R., Kessler M., Macrae C. F., McCabe P., Pearson J., Taylor R. (2002). Acta Crystallogr., Sect. B.

[cit61] Cavazzini M., Quici S., Scalera C., Puntoriero F., La Ganga G., Campagna S. (2009). Inorg. Chem..

[cit62] Nguyen H. L., Gropp C., Ma Y., Zhu C., Yaghi O. M. (2020). J. Am. Chem. Soc..

[cit63] Moorthy J. N., Natarajan P. (2010). Chem. – Eur. J..

[cit64] Yamagishi H., Sato H., Hori A., Sato Y., Matsuda R., Kato K., Aida T. (2018). Science.

[cit65] Liu T., Wang B., He R., Arman H., Schanze K. S., Xiang S., Li D., Chen B. (2020). Can. J. Chem..

[cit66] Carvalho M. F. N. N., Fernanda M. T. A., Adelino M. G., Armando J. L. P. (2003). J. Organomet. Chem..

[cit67] Janiak C. (2000). J. Chem. Soc., Dalton Trans..

[cit68] Macrae C. F., Sovago I., Cottrell S. J., Galek P. T. A., McCabe P., Pidcock E., Platings M., Shields G. P., Stevens J. S., Towler M., Wood P. A. (2020). J. Appl. Crystallogr..

[cit69] Beves J. E., Constable E. C., Housecroft C. E., Kepert C. J., Price D. J. (2007). CrystEngComm.

[cit70] Beves J. E., Constable E. C., Housecroft C. E., Kepert C. J., Neuburger M., Price D. J., Schaffner S., Zampese J. A. (2008). Dalton Trans..

[cit71] Klein Y. M., Constable E. C., Housecroft C. E., Zampese J. A., Crochet A. (2014). CrystEngComm.

[cit72] Rocco D., Novak S., Prescimone A., Constable E. C., Housecroft C. E. (2021). Chemistry.

[cit73] Constable E. C., Housecroft C. E., Neuburger M., Schönle J., Vujovic S., Zampese J. A. (2013). Polyhedron.

[cit74] Rocco D., Manfroni G., Prescimone A., Klein Y. M., Gawryluk D. J., Constable E. C., Housecroft C. E. (2020). Polymer.

[cit75] Klein Y. M., Prescimone A., Constable E. C., Housecroft C. E. (2016). Inorg. Chem. Commun..

[cit76] Barquín M., Cancela J., González Garmendia M. J., Quintanilla J., Amador U. (1998). Polyhedron.

[cit77] Klein Y. M., Constable E. C., Housecroft C. E., Prescimone A. (2014). Inorg. Chem. Commun..

[cit78] Liu C.-L., Zheng C.-J., Liu X.-K., Chen Z., Yang J.-P., Li F., Ou X.-M., Zhang X.-H. (2015). J. Mater. Chem. C.

[cit79] Anthony J. E., Khan S. I., Rubin Y. (1997). Tetrahedron Lett..

[cit80] Sheldrick G. (2015). Acta Crystallogr., Sect. A.

[cit81] Dolomanov O. V., Bourhis L. J., Gildea R. J., Howard J. A. K., Puschmann H. (2009). J. Appl. Crystallogr..

[cit82] Sheldrick G. (2015). Acta Crystallogr., Sect. C.

[cit83] Kabsch W. (2010). Acta Crystallogr., Sect. D.

[cit84] Rodríguez-Carvajal J. (1993). Phys. B.

[cit85] Roisnel T. and Rodríguez-Carvajal J., in In Materials Science Forum. Proceedings of the European Powder Diffraction Conf. (EPDIC 7), Barcelona, Spain, 2001, pp. 118–123

