# Supplementary material for: Stars and stripes: hexatopic tris(3,2′:6′,3′′-terpyridine) ligands that unexpectedly form one-dimensional coordination polymers
Source: CrystEngComm. 2021 Dec 6;24(3):491–503. doi: 10.1039/d1ce01531a (PMC8764615; doi:10.1039/d1ce01531a)
Supplement: CE-024-D1CE01531A-s001 [file CE-024-D1CE01531A-s001.pdf]

Electronic Supplementary Material (ESI) for CrystEngComm.
This journal is © The Royal Society of Chemistry 2021

Supporting Material

Stars and stripes: hexatopic tris(3,2':6',3''-terpyridine) ligands that unexpectedly form one-dimensional coordination polymers

Giacomo Manfroni, Alessandro Prescimone, Edwin C. Constable, and Catherine E. Housecroft*

Department of Chemistry, University of Basel, BPR 1096, Mattenstrasse 24a, CH-4058 Basel, Switzerland

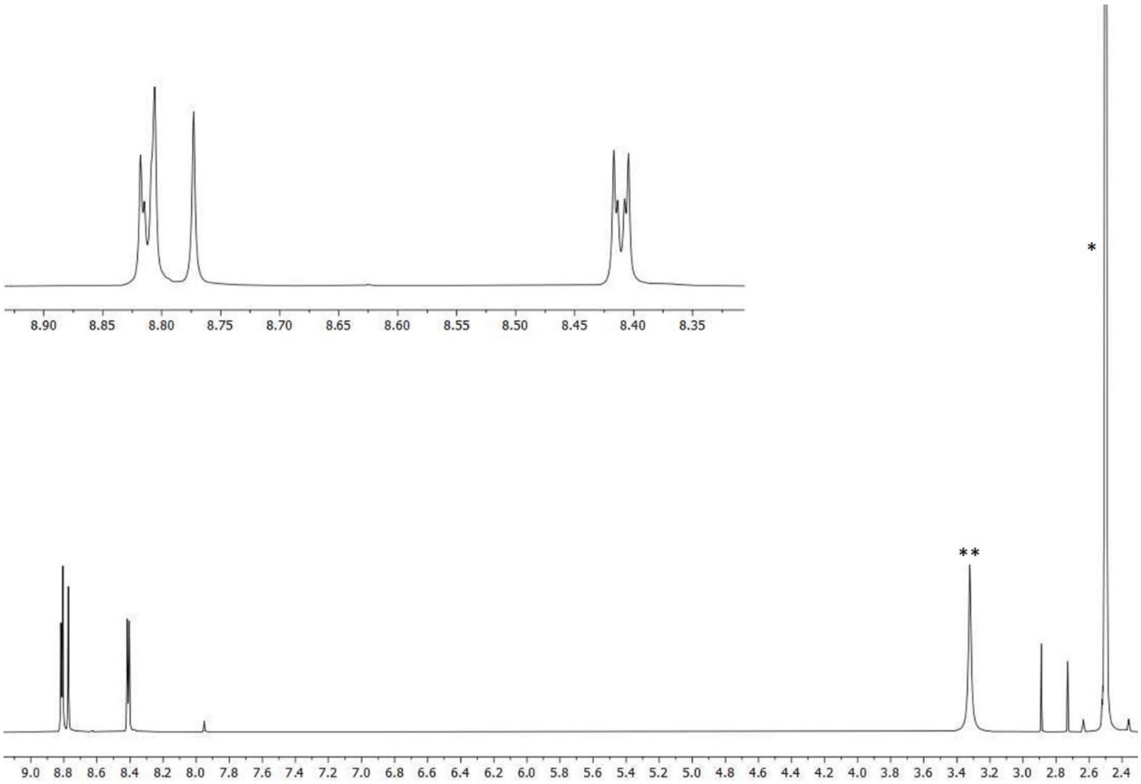

Figure S1. ^{1}H NMR spectrum (500 MHz, DMSO-d$_6$, 298 K) of **1**. * = DMSO-d$_5$; ** = H_2O.

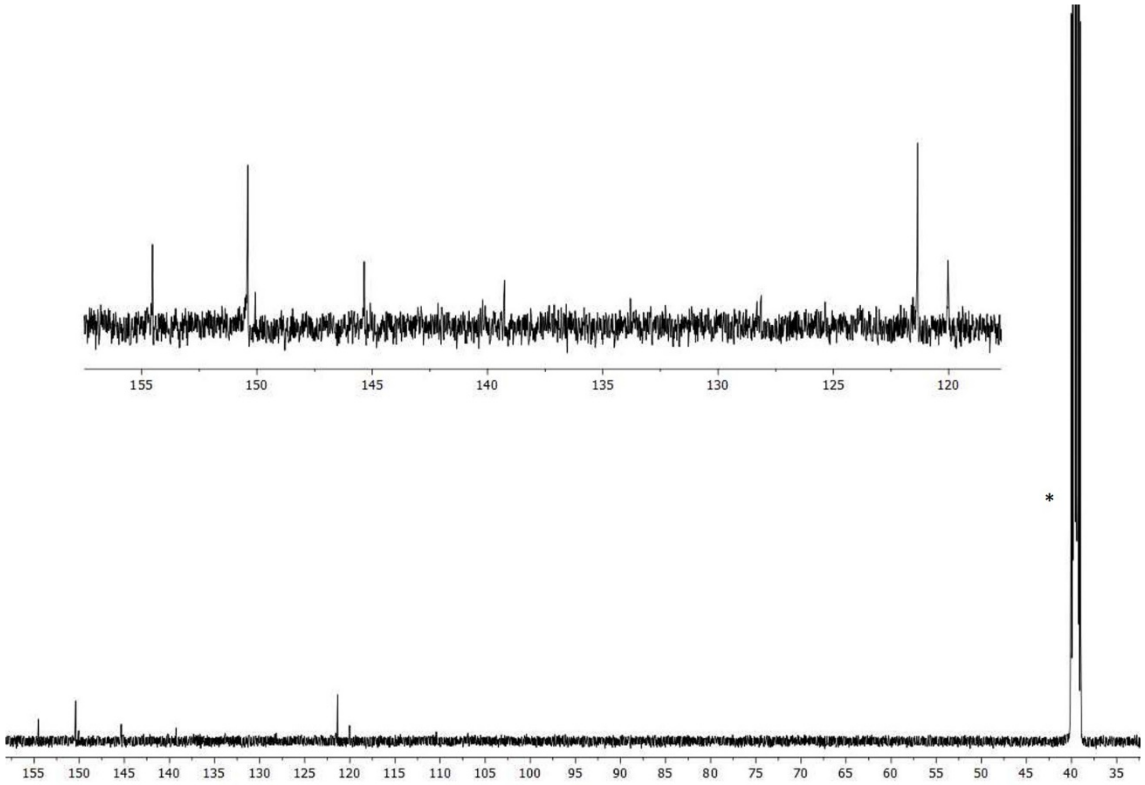

Figure S2. $^{13}C\{^{1}H\}$ NMR spectrum (126 MHz, DMSO-d_6, 298 K) of **1**. * = DMSO-d_6.

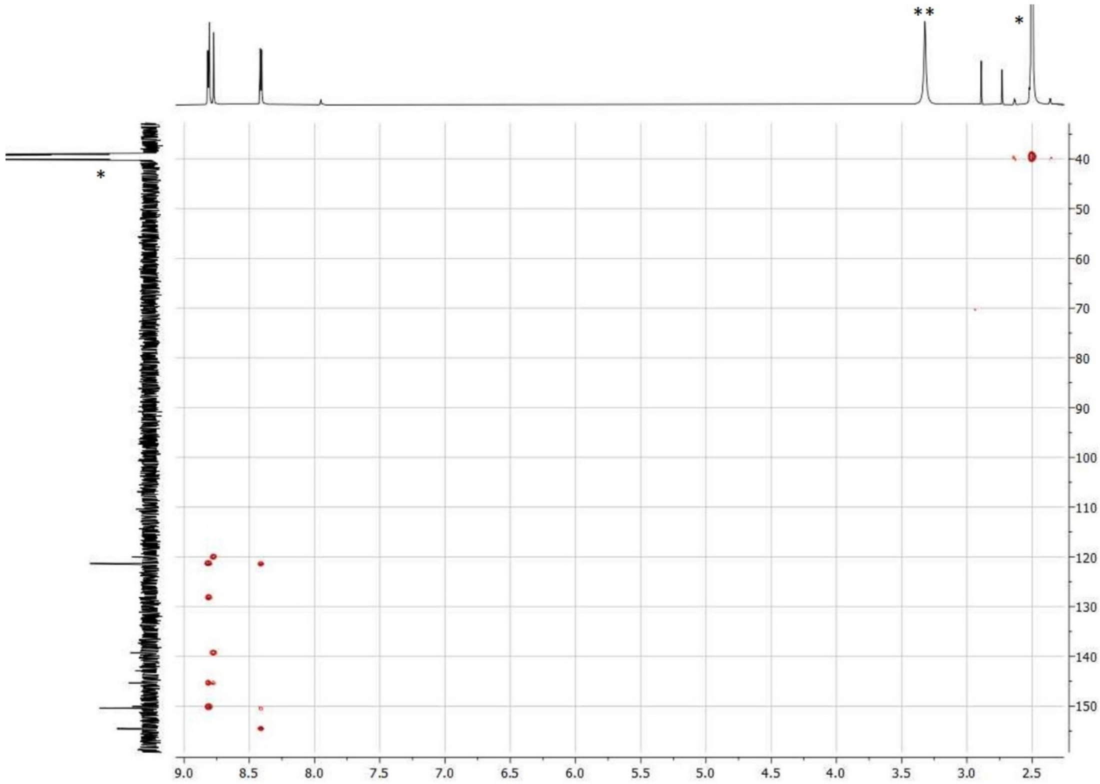

Figure S3. HMBC spectrum (^{1}H 500 MHz, $^{13}C\{^{1}H\}$ 126 MHz, DMSO-d_6, 298 K) of compound **1**. * = DMSO-d_6, ** = H_2O.

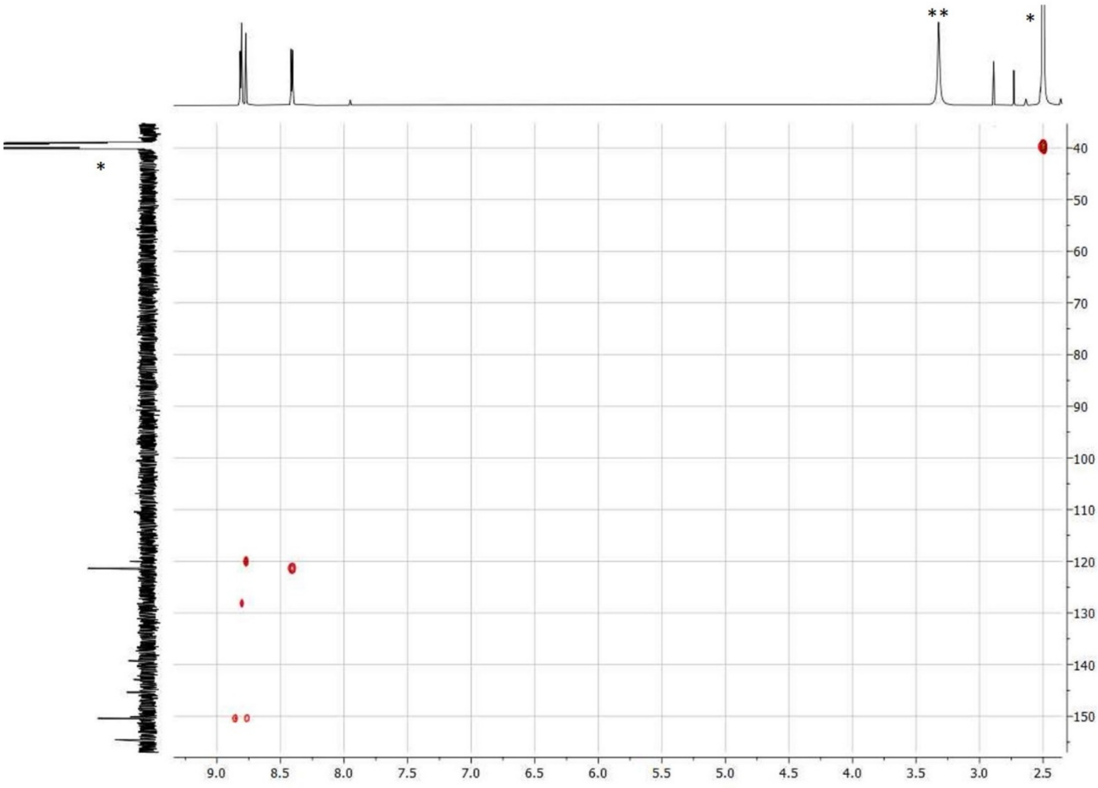

Figure S4. HMQC spectrum (^{1}H 500 MHz, $^{13}C\{^1H\}$ 126 MHz, DMSO-d_6, 298 K) of compound **1**. * = DMSO-d_6, ** = H_2O.

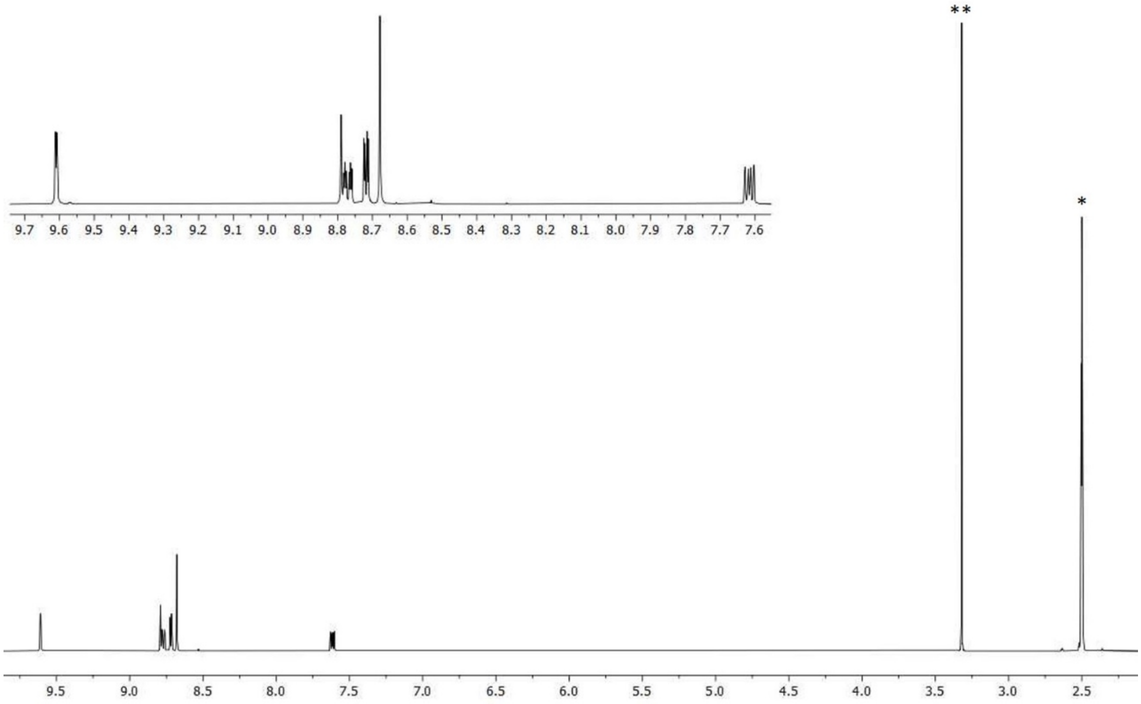

Figure S5. ^{1}H NMR spectrum (500 MHz, DMSO-d_6, 298 K) of **2**. * = DMSO-d_5; ** = H_2O.

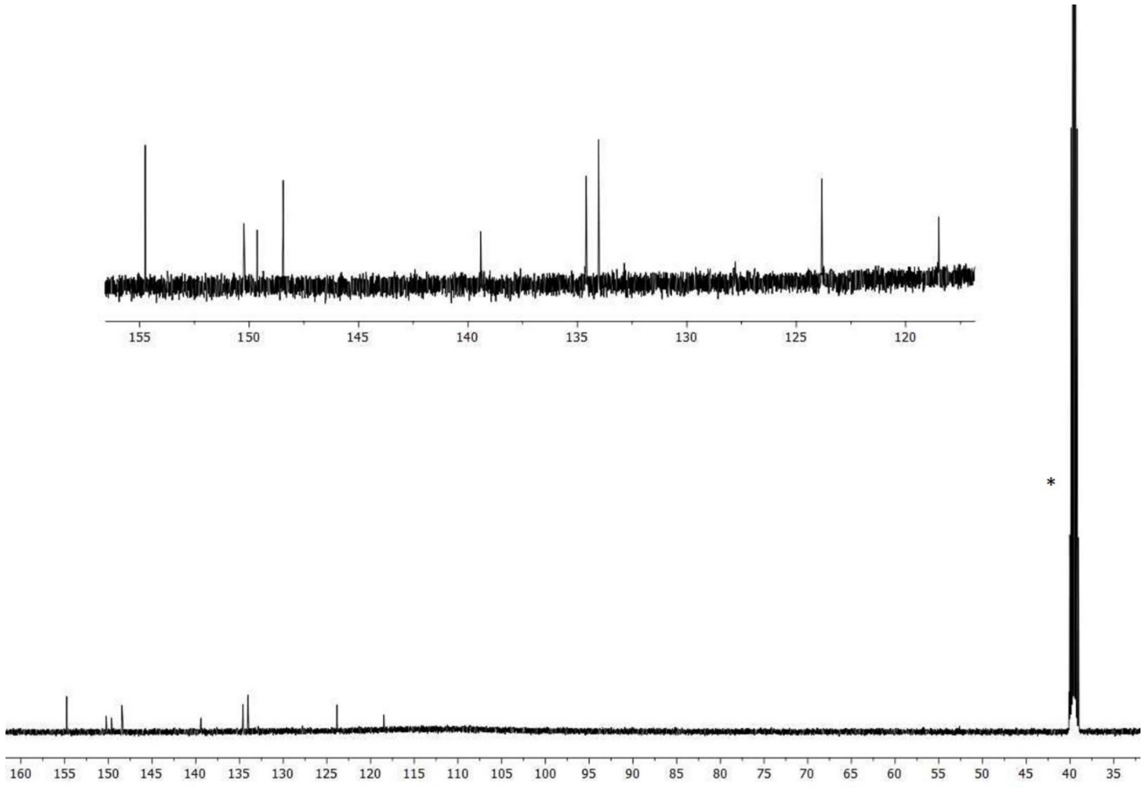

Figure S6. $^{13}C\{^{1}H\}$ NMR spectrum (126 MHz, DMSO-d_6, 298 K) of **2**. * = DMSO-d_6.

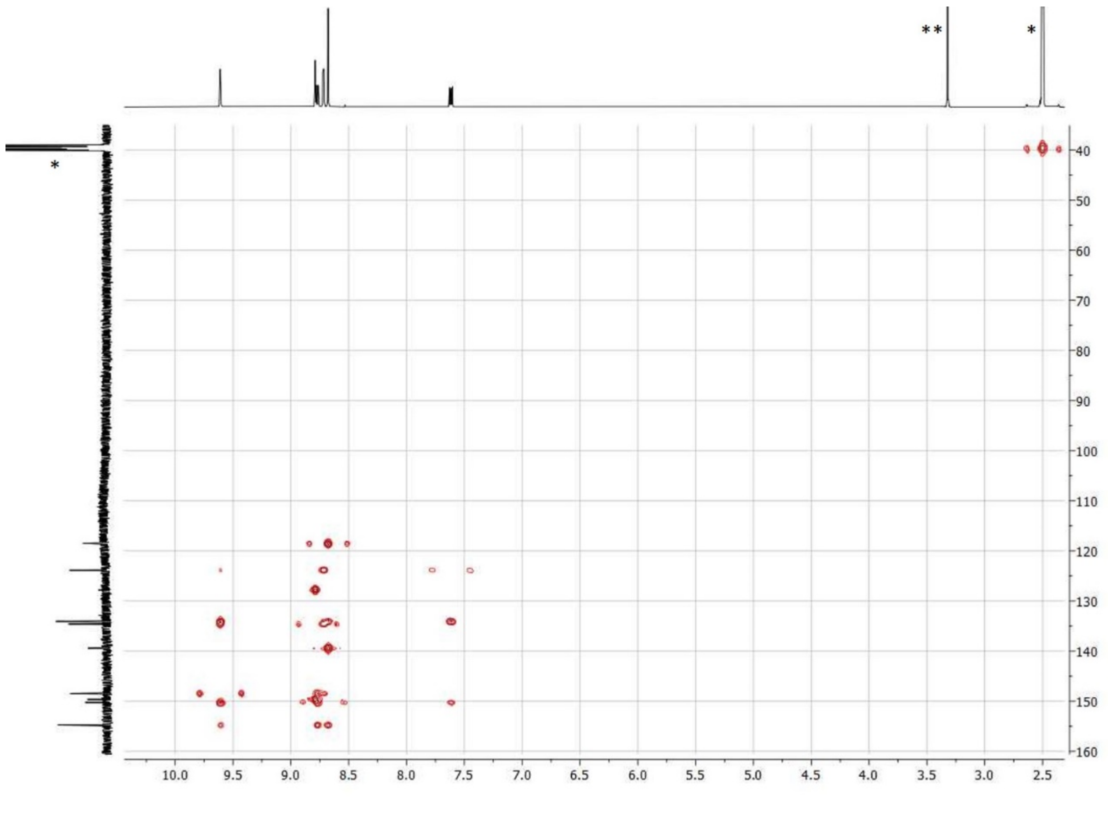

Figure S7. HMBC spectrum (^{1}H 500 MHz, $^{13}C\{^{1}H\}$ 126 MHz, DMSO-d_6, 298 K) of compound **2**. * = DMSO-d_6, ** = H_2O.

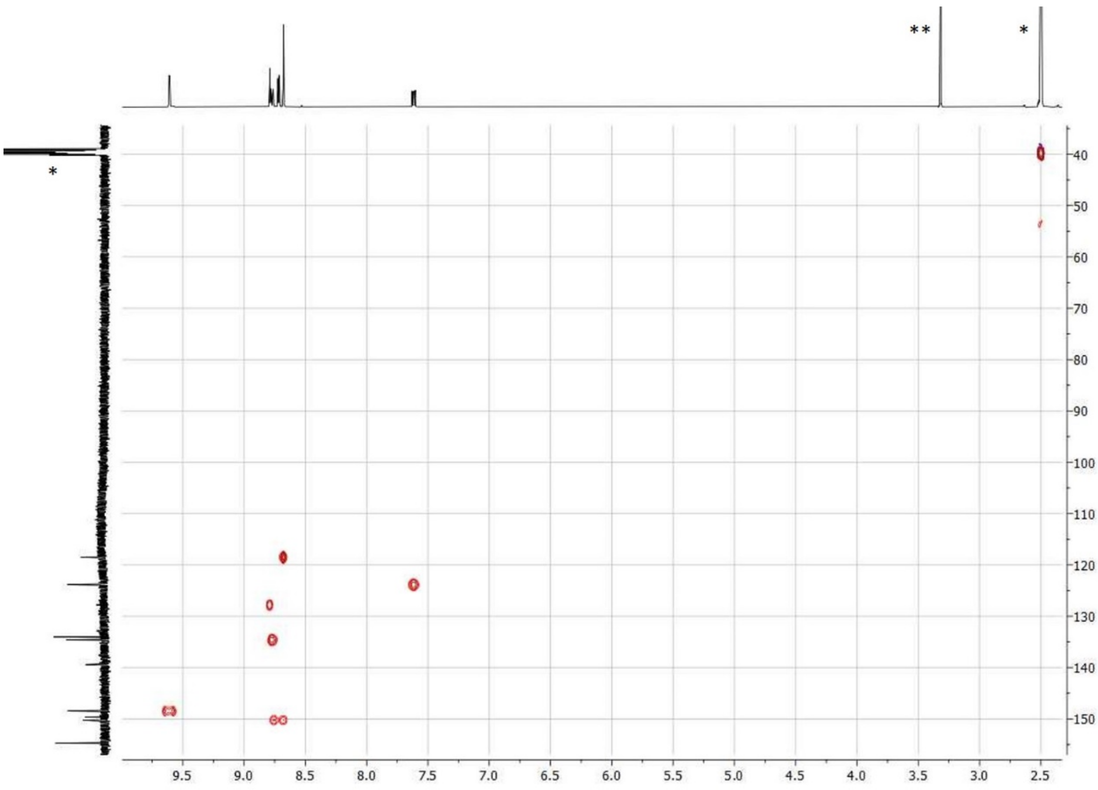

Figure S8. HMQC spectrum (^{1}H 500 MHz, ^{13}C{^{1}H} 126 MHz, DMSO-d$_6$, 298 K) of compound **2**. * = DMSO-d$_6$, ** = H_2O.

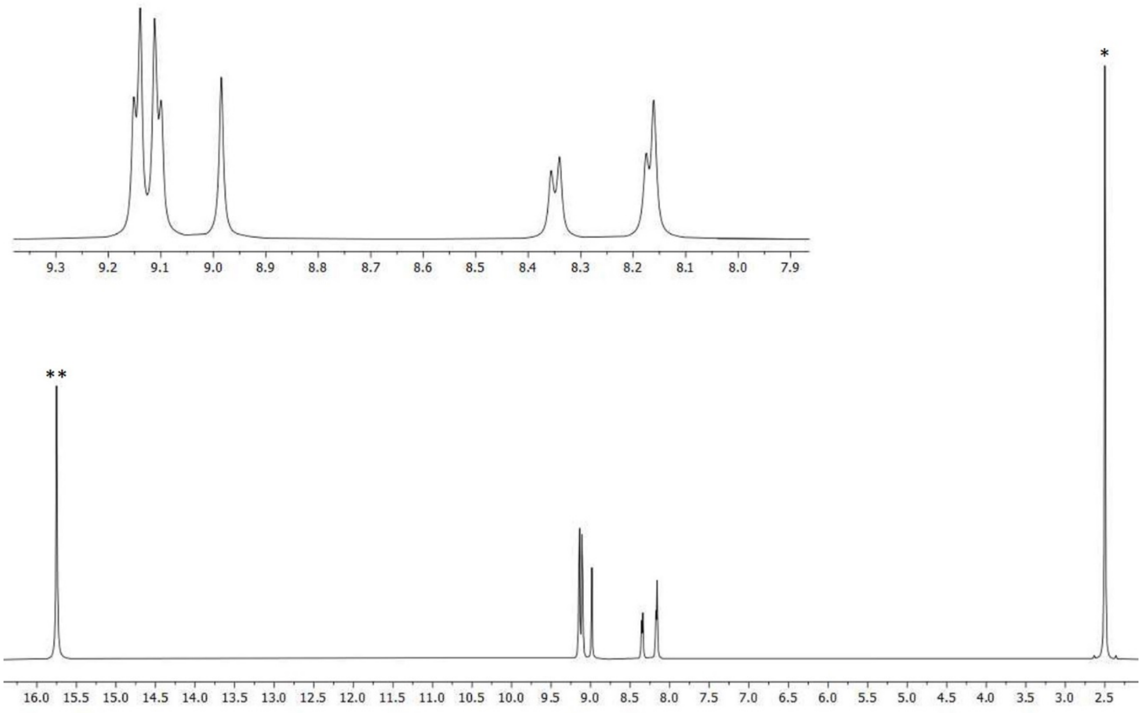

Figure S9. ^{1}H NMR spectrum (500 MHz, DMSO-d$_6$ + TFA-d, 298 K) of **3**. * = DMSO-d$_5$; ** = CF_3COOH.

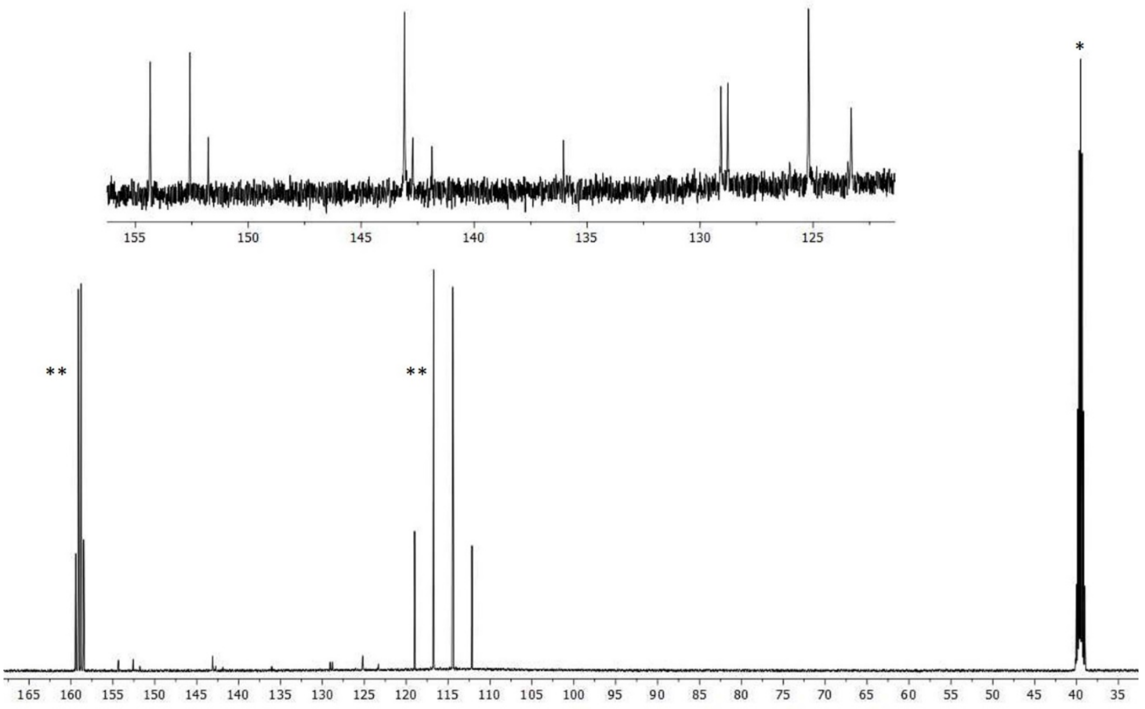

Figure S10. $^{13}C\{^{1}H\}$ NMR spectrum (126 MHz, DMSO-d_6 + TFA-d, 298 K) of **3**. * = DMSO-d_6; ** = CF_3COOD.

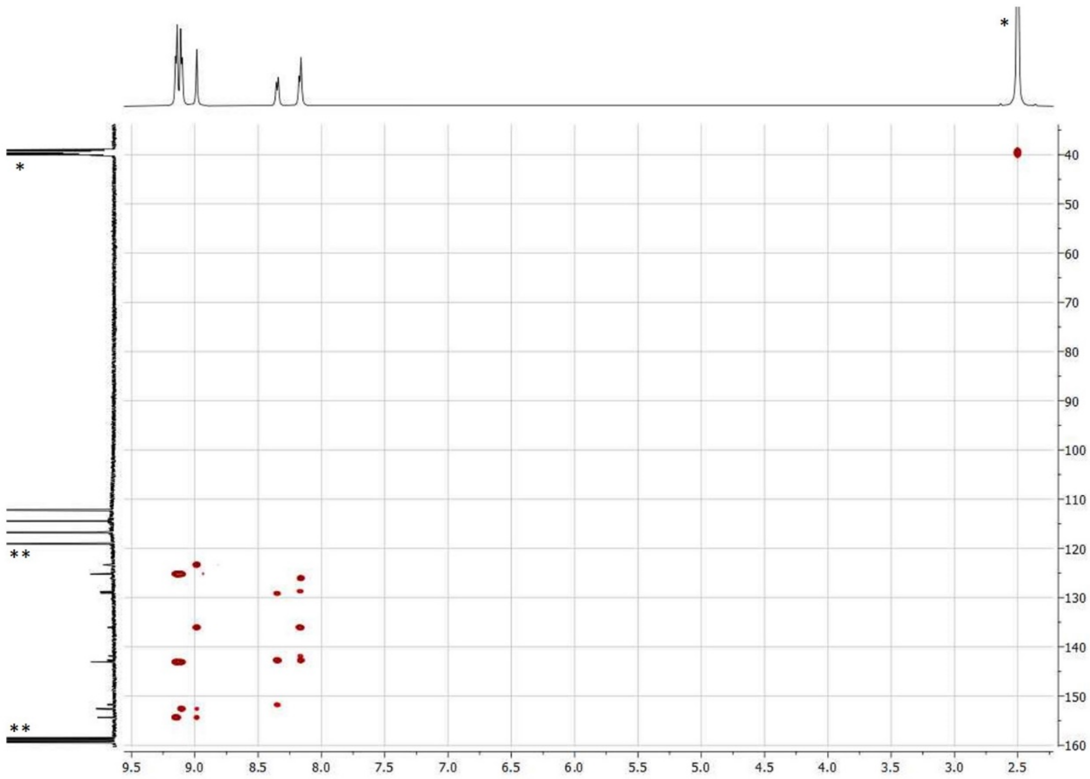

Figure S11. HMBC spectrum (^{1}H 500 MHz, $^{13}C\{^{1}H\}$ 126 MHz, DMSO-d_6 + TFA-d, 298 K) of **3**. * = DMSO-d_6; ** = CF_3COOD.

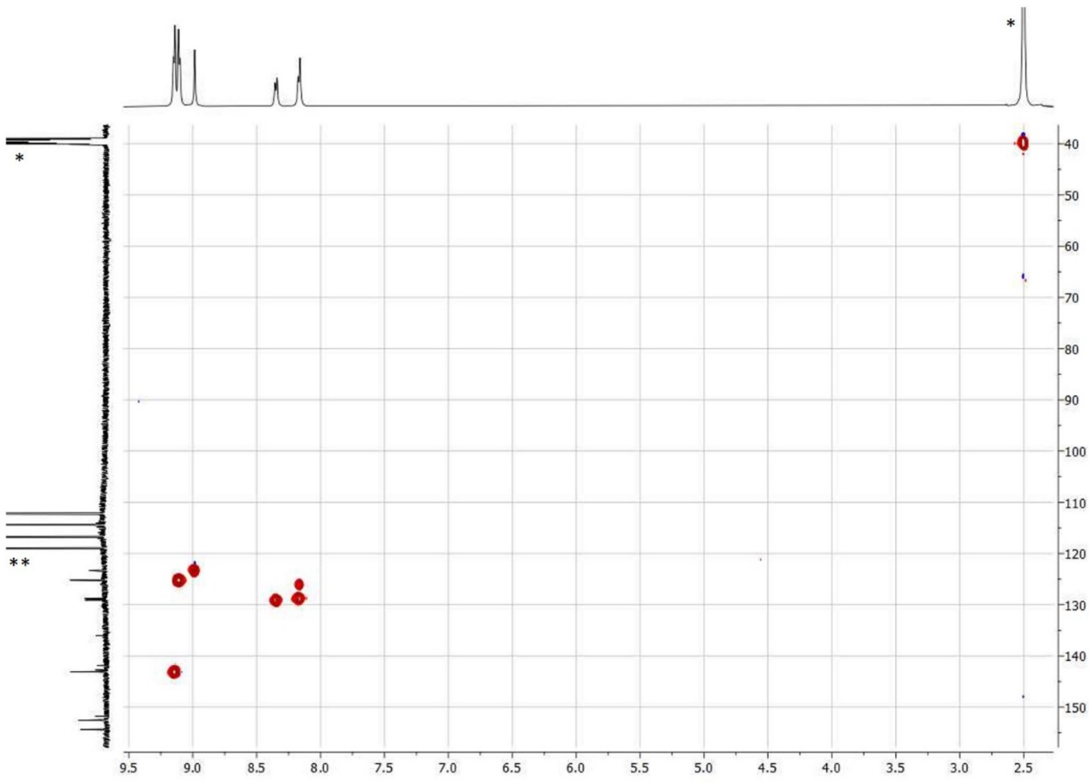

Figure S12. HMQC spectrum (^{1}H 500 MHz, $^{13}C\{^{1}H\}$ 126 MHz, DMSO-d_6 + TFA-d, 298 K) of **3**. * = DMSO-d_6; ** = CF_3COOD.

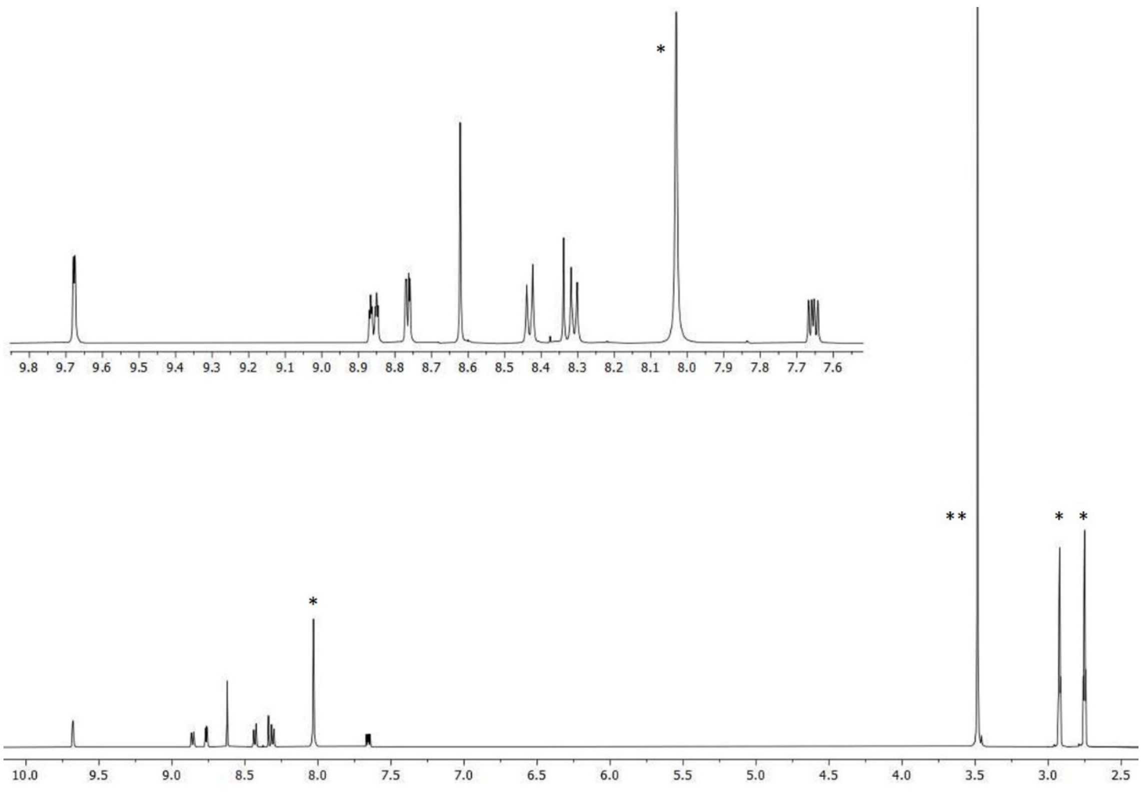

Figure S13. ^{1}H NMR spectrum (500 MHz, DMF-d_7, 298 K) of **4**. * = DMF-d_6; ** = H_2O.

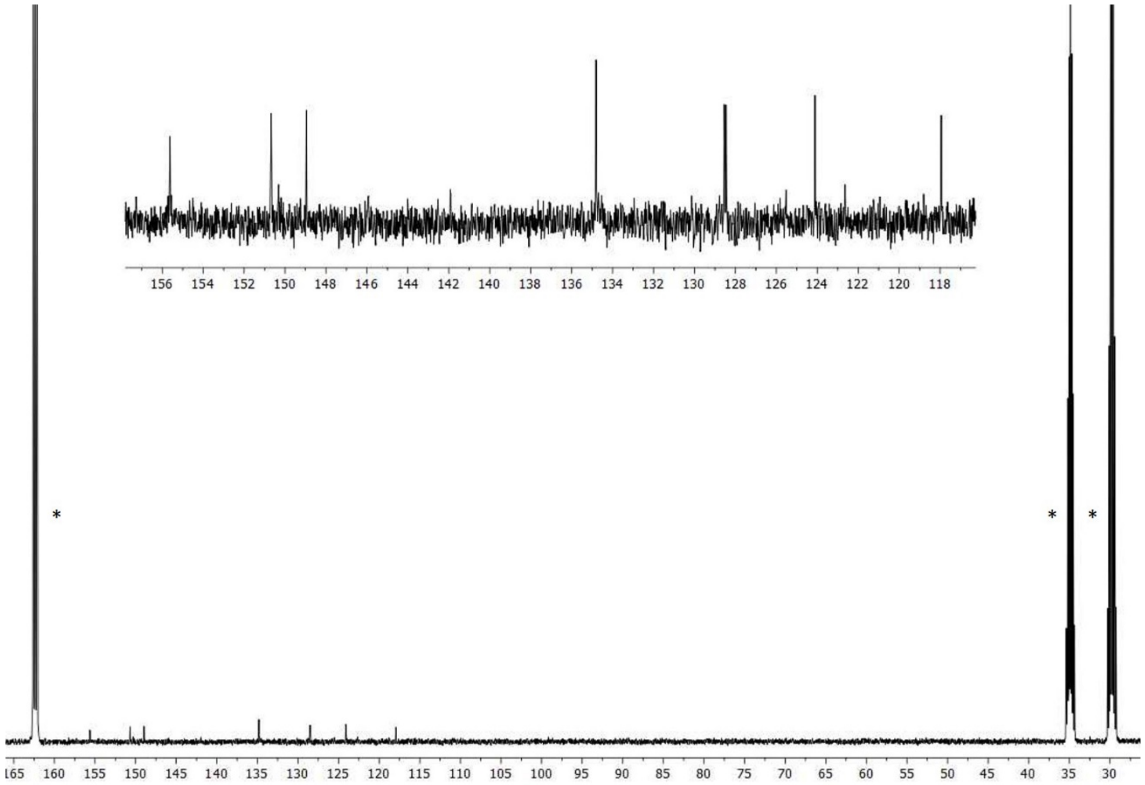

Figure S14. $^{13}C\{^1H\}$ NMR spectrum (126 MHz, DMF-d_7, 298 K) of **4**. * = DMF-d_7.

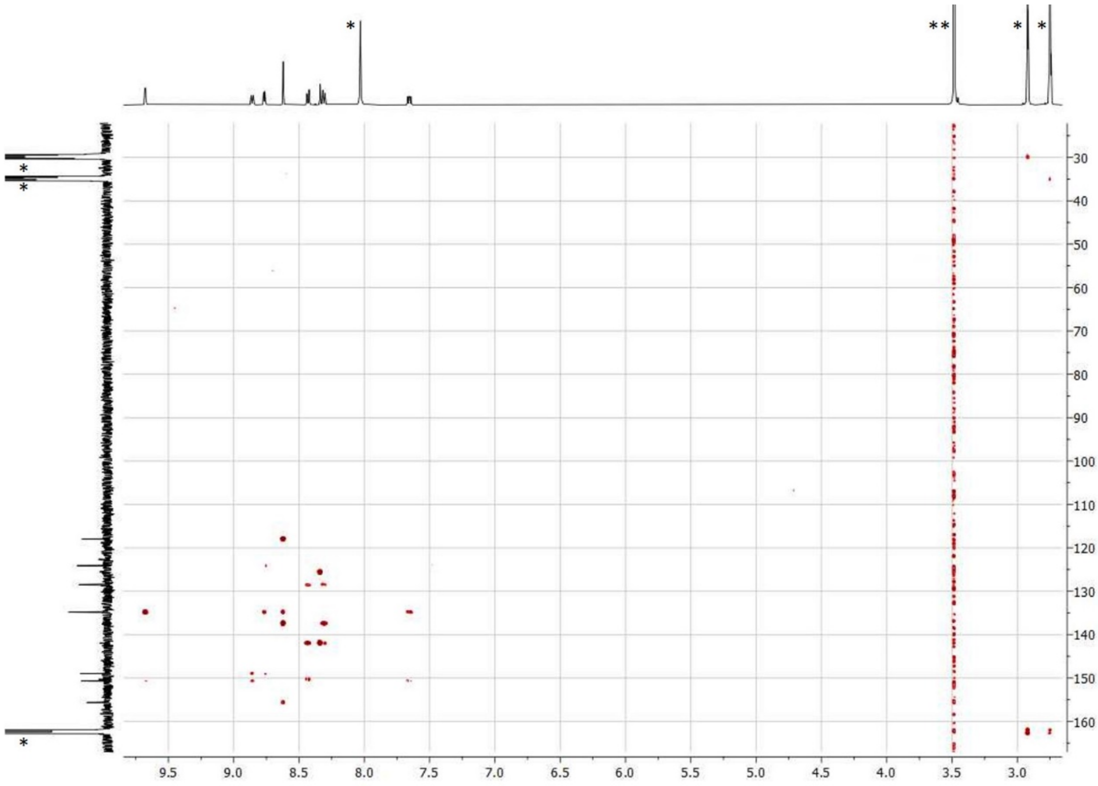

Figure S15. HMBC spectrum (^1H 500 MHz, $^{13}C\{^1H\}$ 126 MHz, DMF-d_7, 298 K) of **4**. * = DMF-d_7; ** = H_2O.

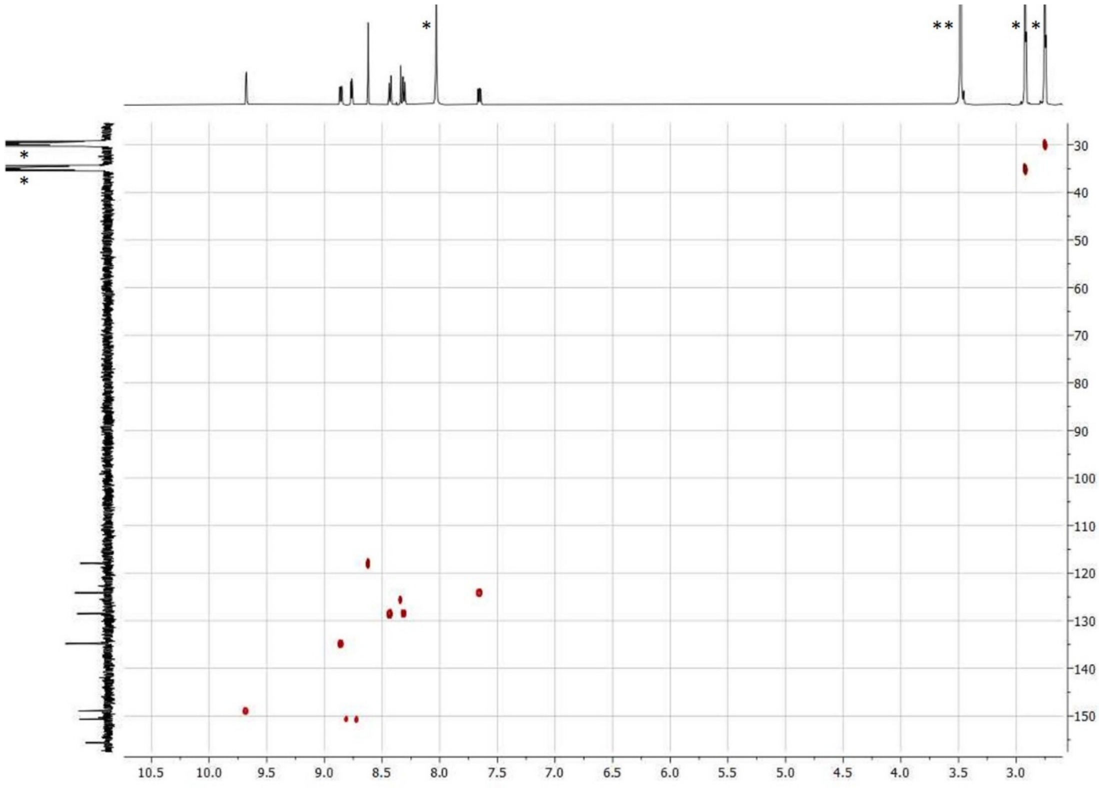

Figure S16. HMQC spectrum (^1H 500 MHz, $^{13}C\{^1H\}$ 126 MHz, DMF-d_7, 298 K) of **4**. * = DMF-d_7; ** = H_2O.

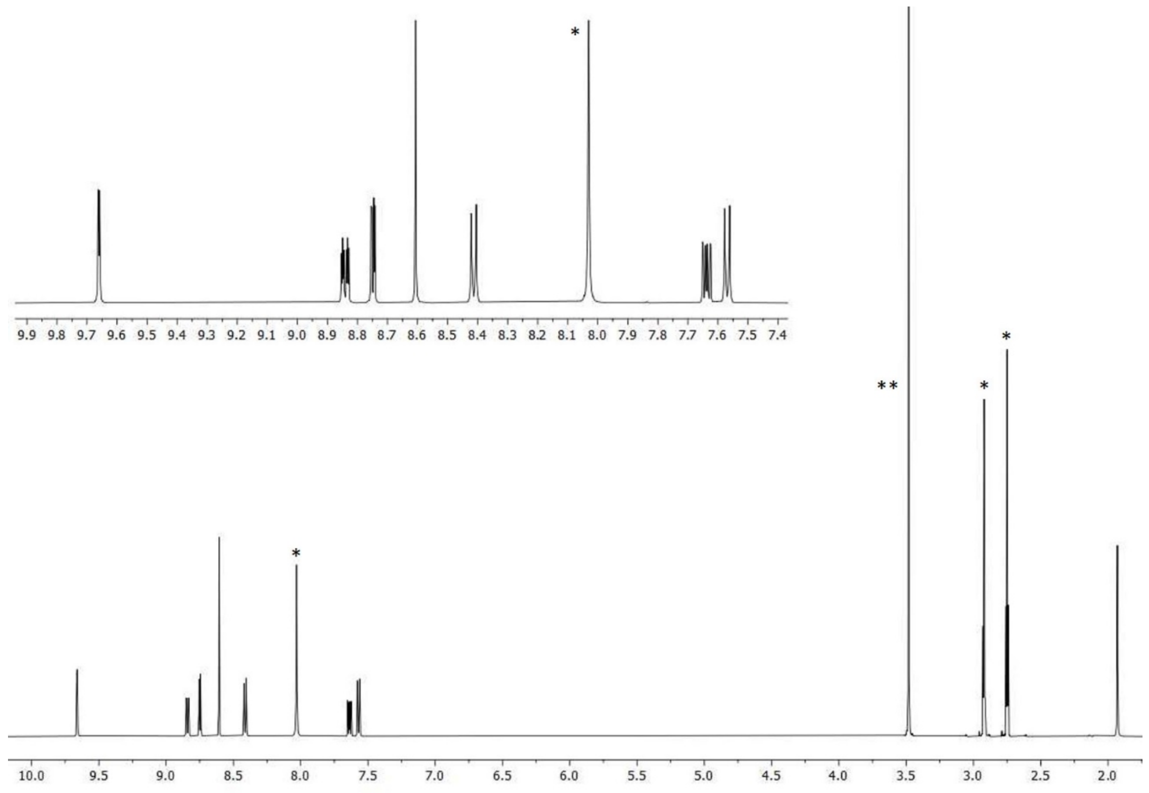

Figure S17. ^1H NMR spectrum (500 MHz, DMF-d_7, 298 K) of **5**. * = DMF-d_6; ** = H_2O.

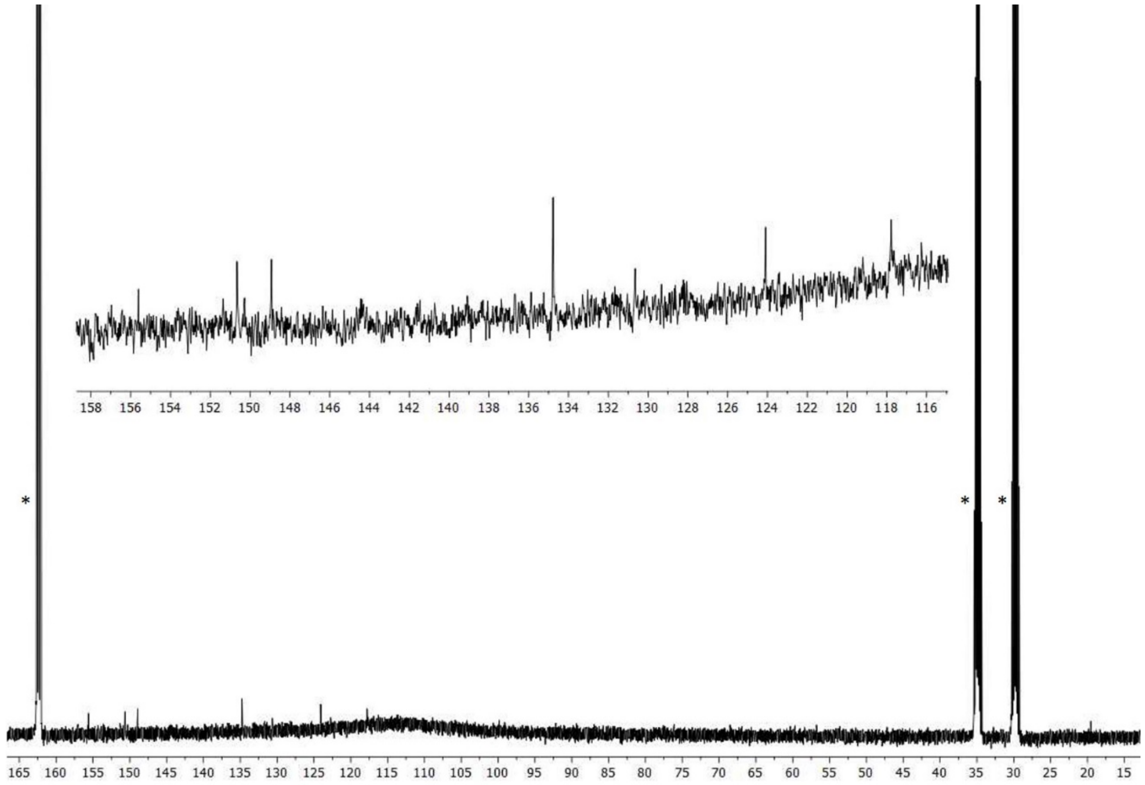

Figure S18. $^{13}C\{^{1}H\}$ NMR spectrum (126 MHz, DMF-d_7, 298 K) of **5**. * = DMF-d_7.

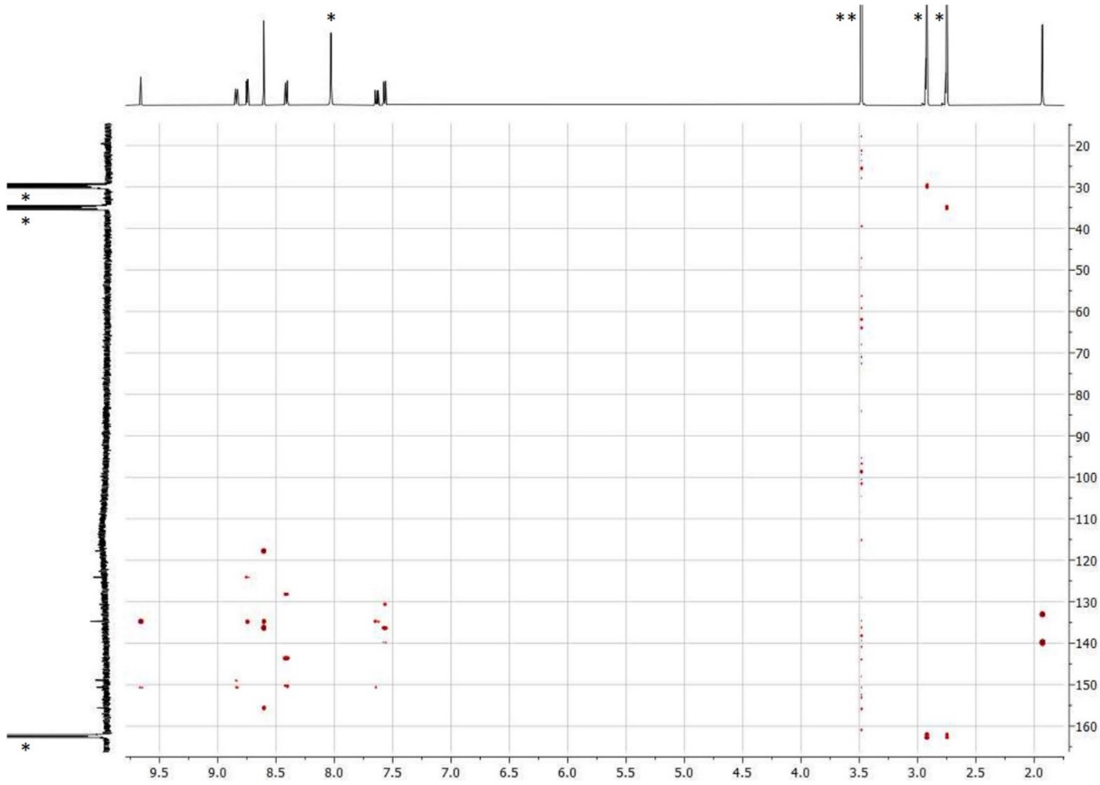

Figure S19. HMBC spectrum (^{1}H 500 MHz, $^{13}C\{^{1}H\}$ 126 MHz, DMF-d_7, 298 K) of **5**. * = DMF-d_7; ** = H_2O.

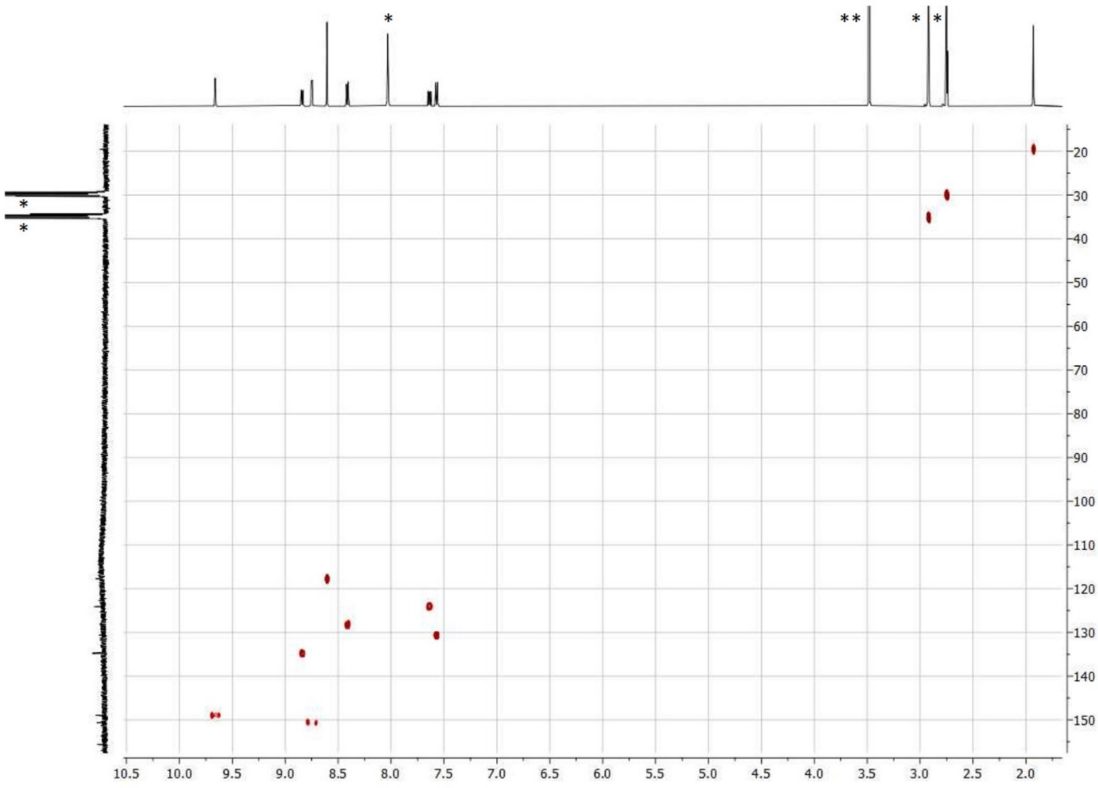

Figure S20. HMQC spectrum (^1H 500 MHz, $^{13}C\{^1H\}$ 126 MHz, DMF-d$_7$, 298 K) of **5**. * = DMF-d$_7$ ** = H_2O.

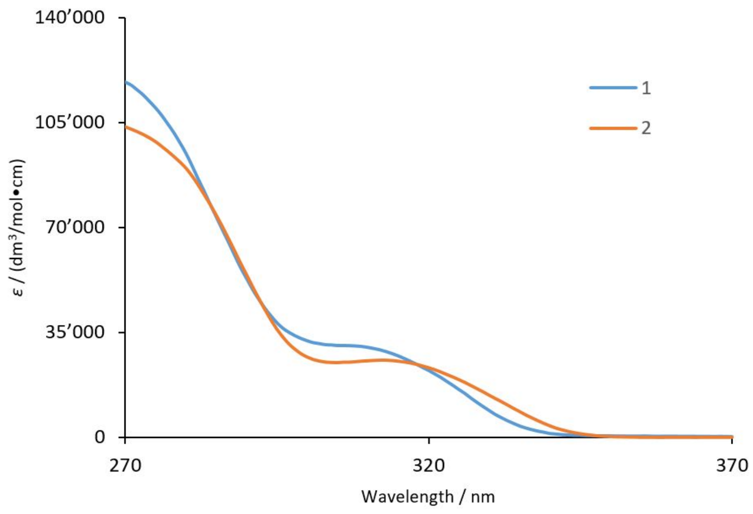

Figure S21. Solution absorption spectra of **1** and **2** in DMF (1.0×10^{-5} mol dm^{-3}).

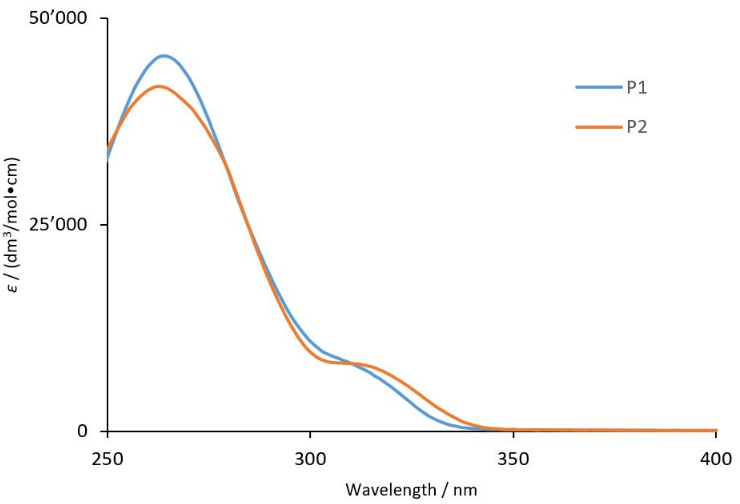

Figure S22. Solution absorption spectra of **P1** and **P2** in $CHCl_3$ (2.6×10^{-5} mol dm^{-3}).

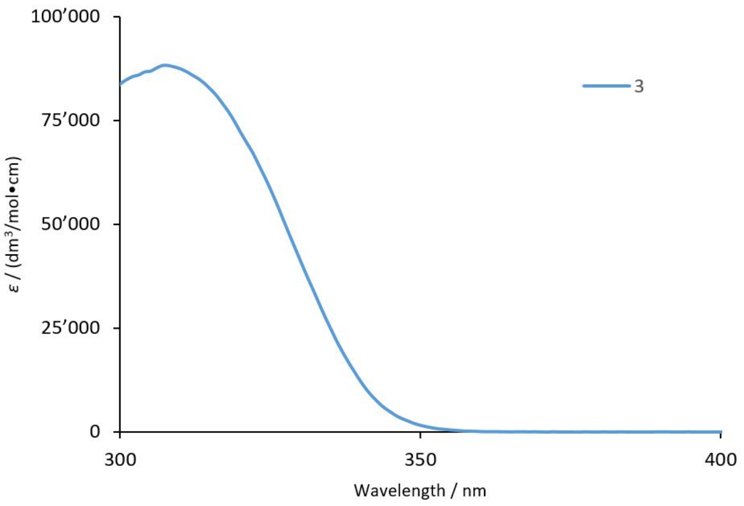

Figure S23. Solution absorption spectrum of **3** in benzonitrile (8.6×10^{-6} mol dm^{-3}).

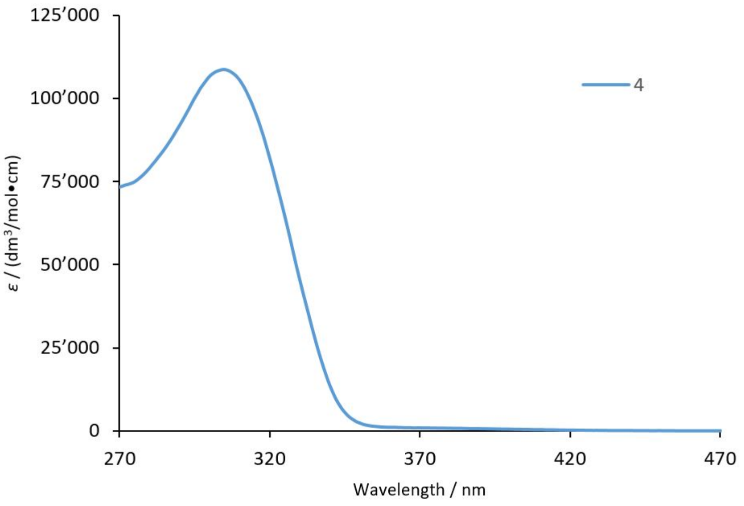

Figure S24. Solution absorption spectrum of **4** in DMF (8.0×10^{-6} mol dm^{-3}).

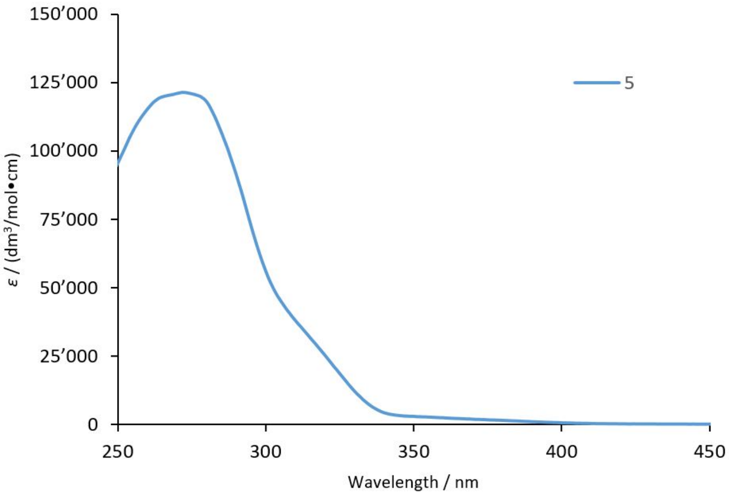

Figure S25. Solution absorption spectrum of **5** in $CHCl_3$ (8.0×10^{-6} mol dm^{-3}).

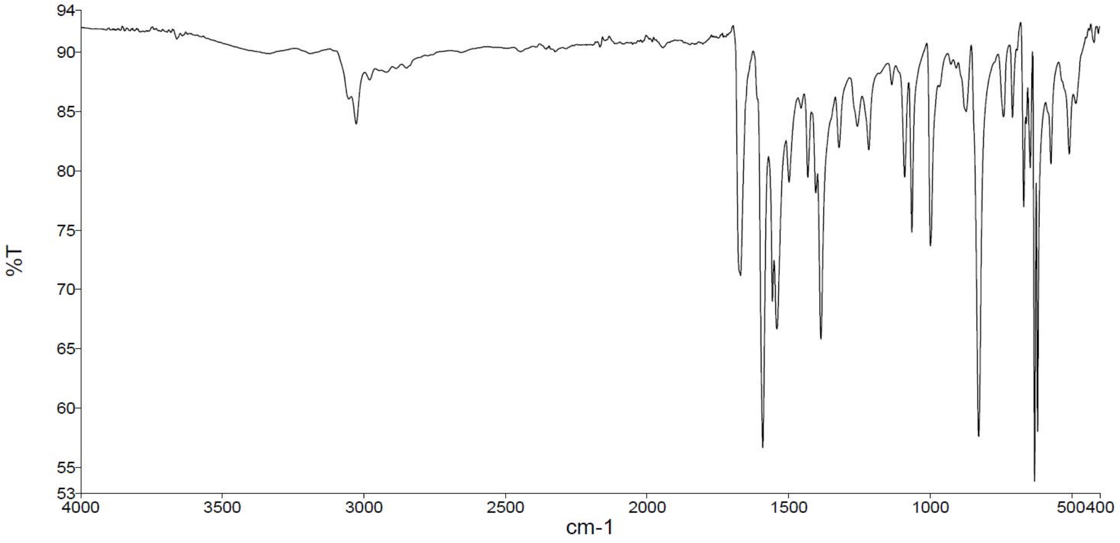

Figure S26. The solid state FT-IR spectrum of **1**.

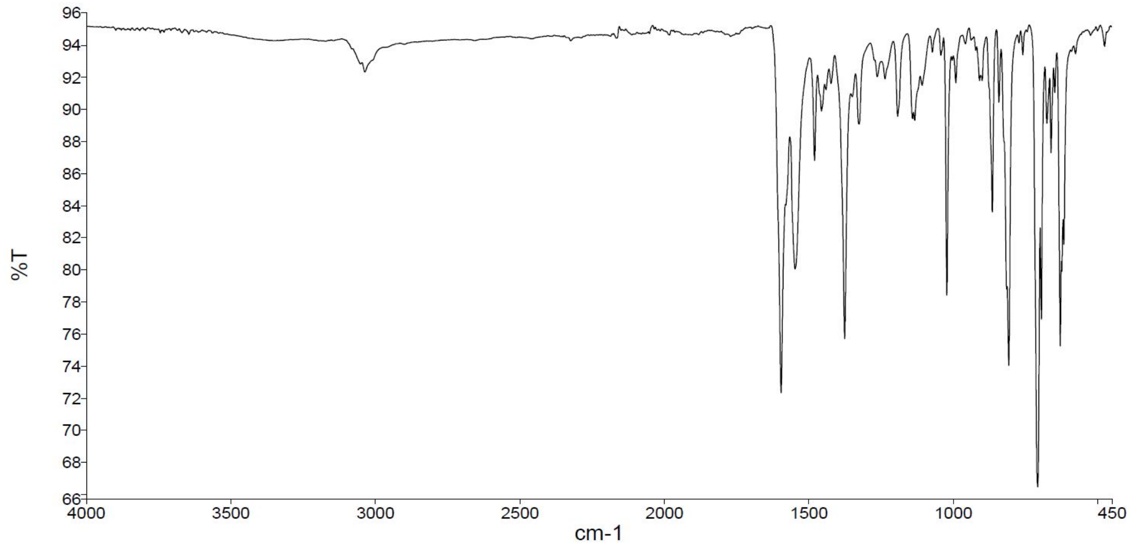

Figure S27. The solid state FT-IR spectrum of **2**.

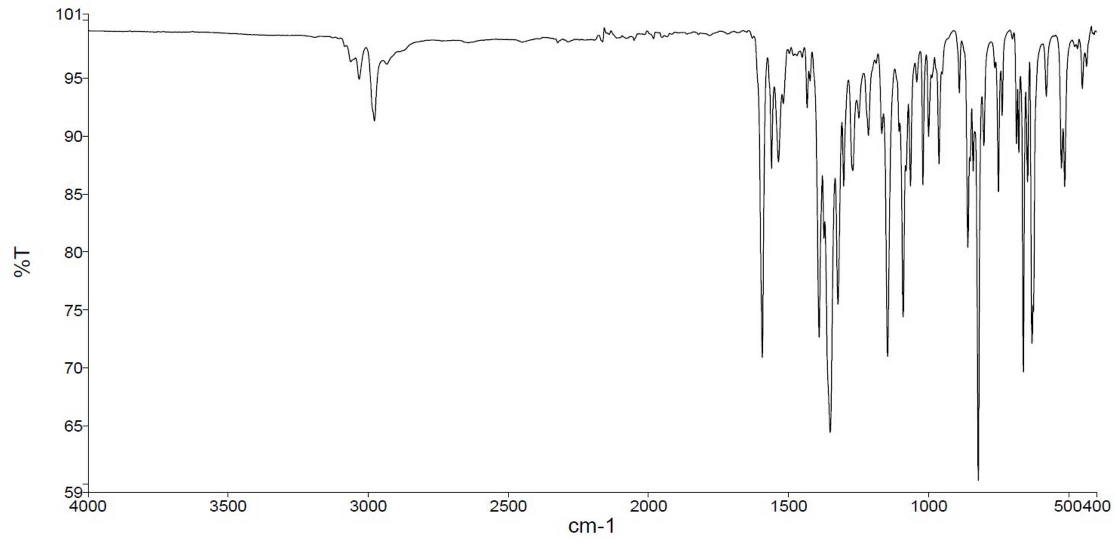

Figure S28. The solid state FT-IR spectrum of **P1**.

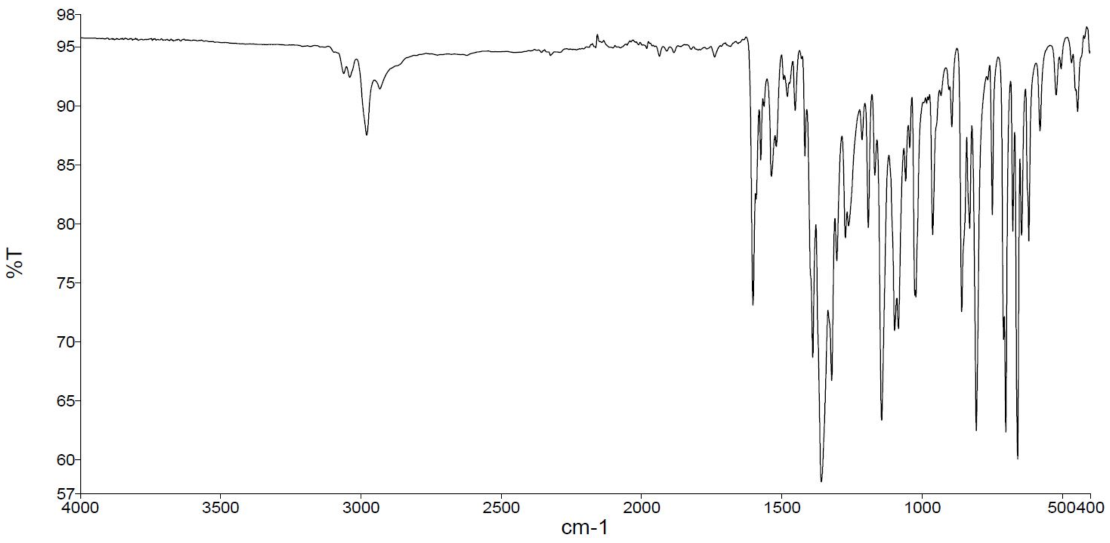

Figure S29. The solid state FT-IR spectrum of **P2**.

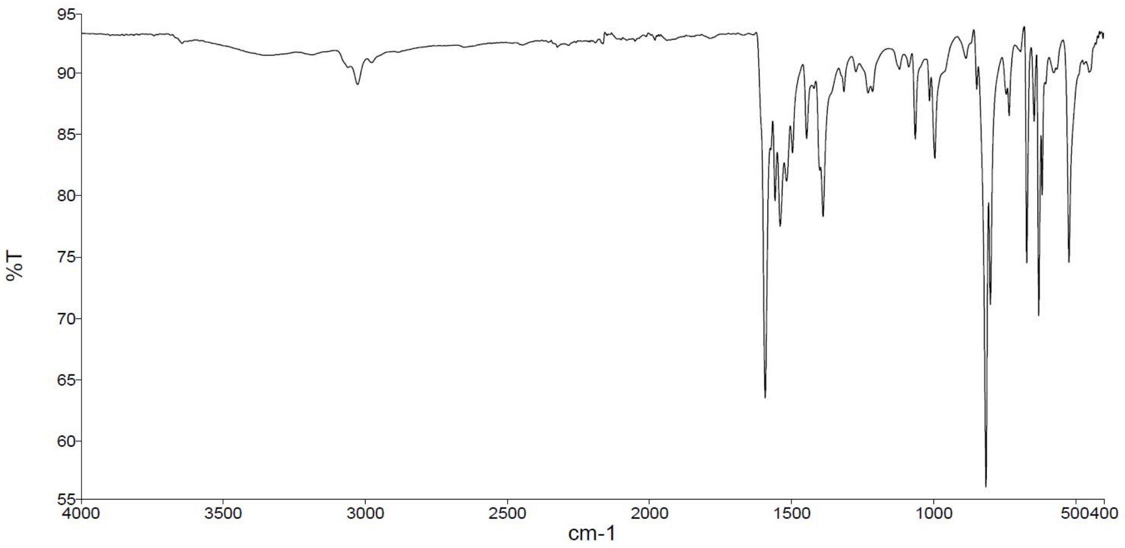

Figure S30. The solid state FT-IR spectrum of **3**.

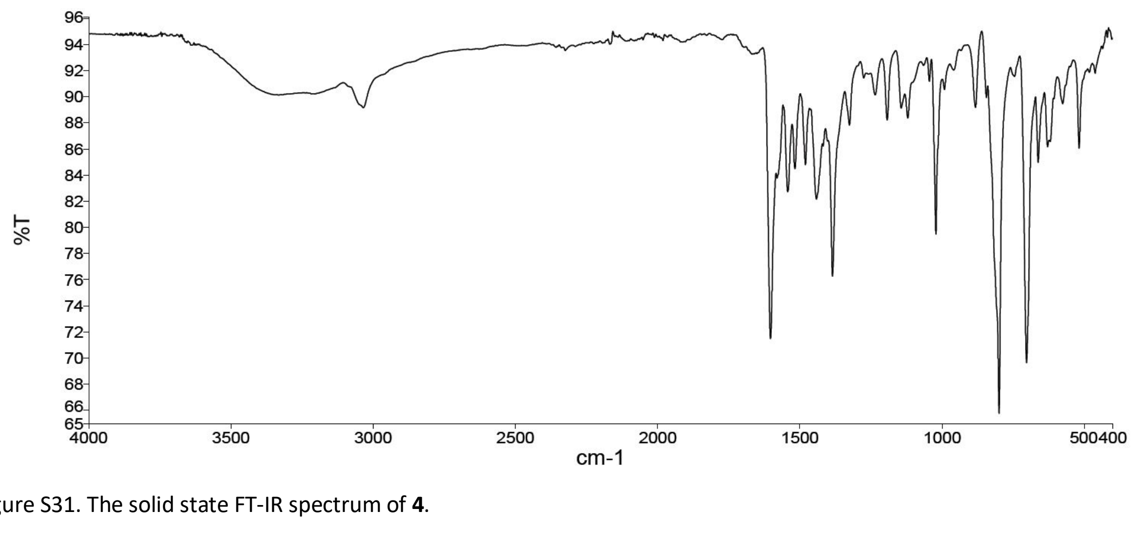

Figure S31. The solid state FT-IR spectrum of **4**.

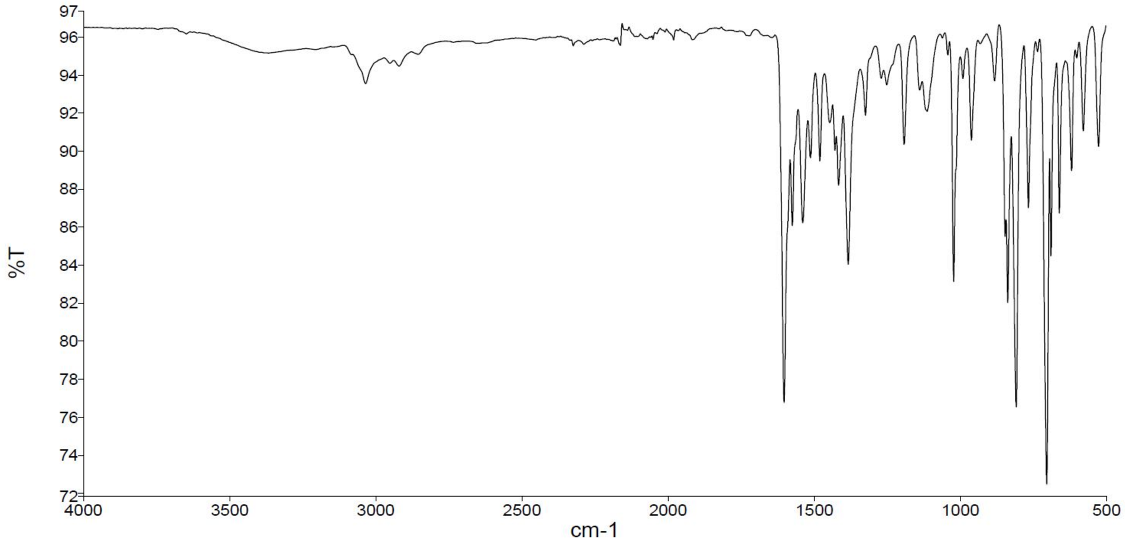

Figure S32. The solid state FT-IR spectrum of **5**.

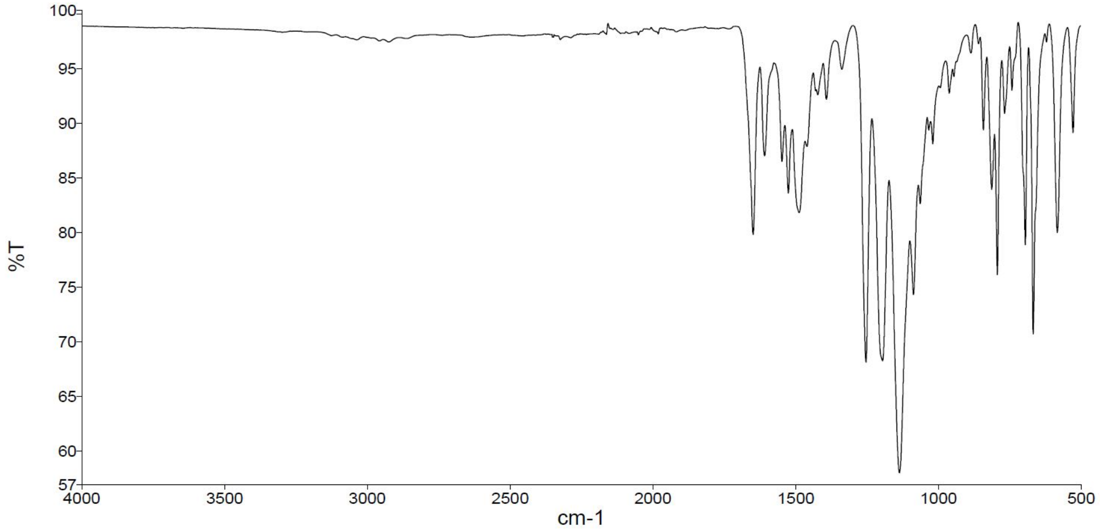

Figure S33. The solid state FT-IR spectrum of $[Cu_3(hfacac)_6(\mathbf{5})]_n{\cdot}0.5nC_7H_8$.

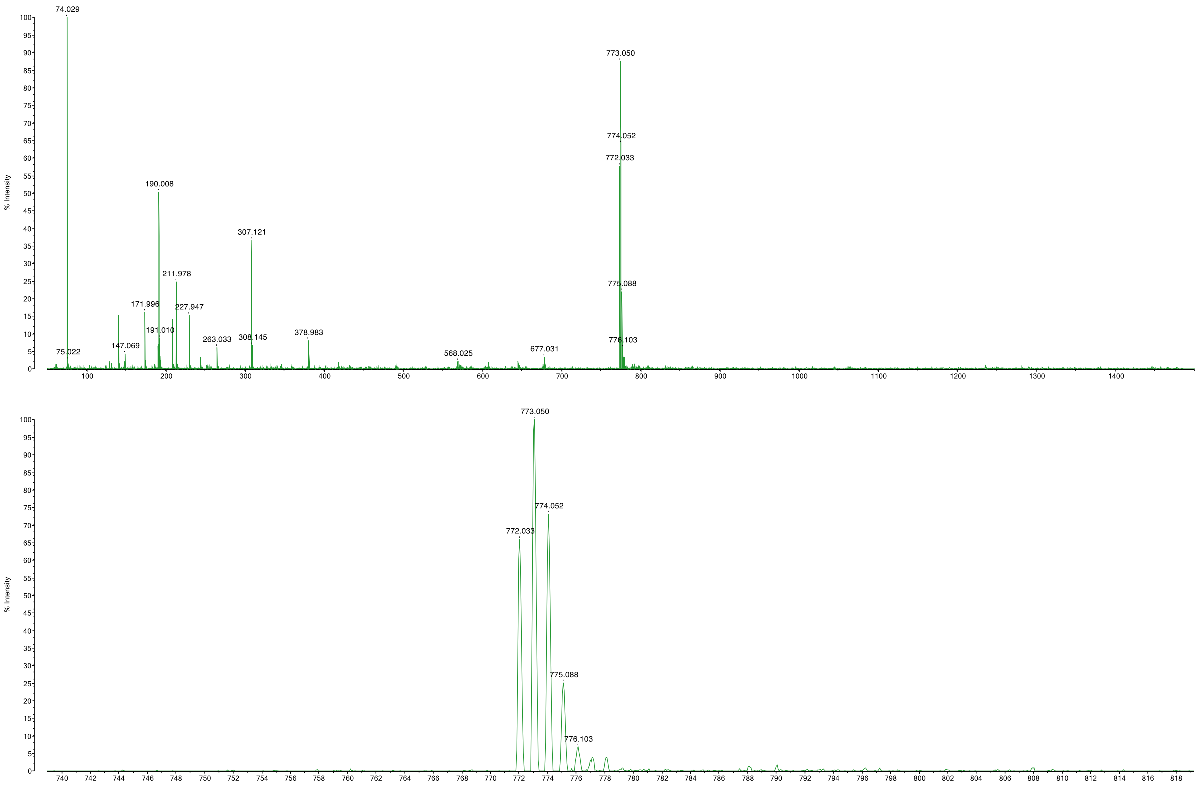

Figure S34. The MALDI-TOF mass spectrum of **1**, and expansion of the base peak.

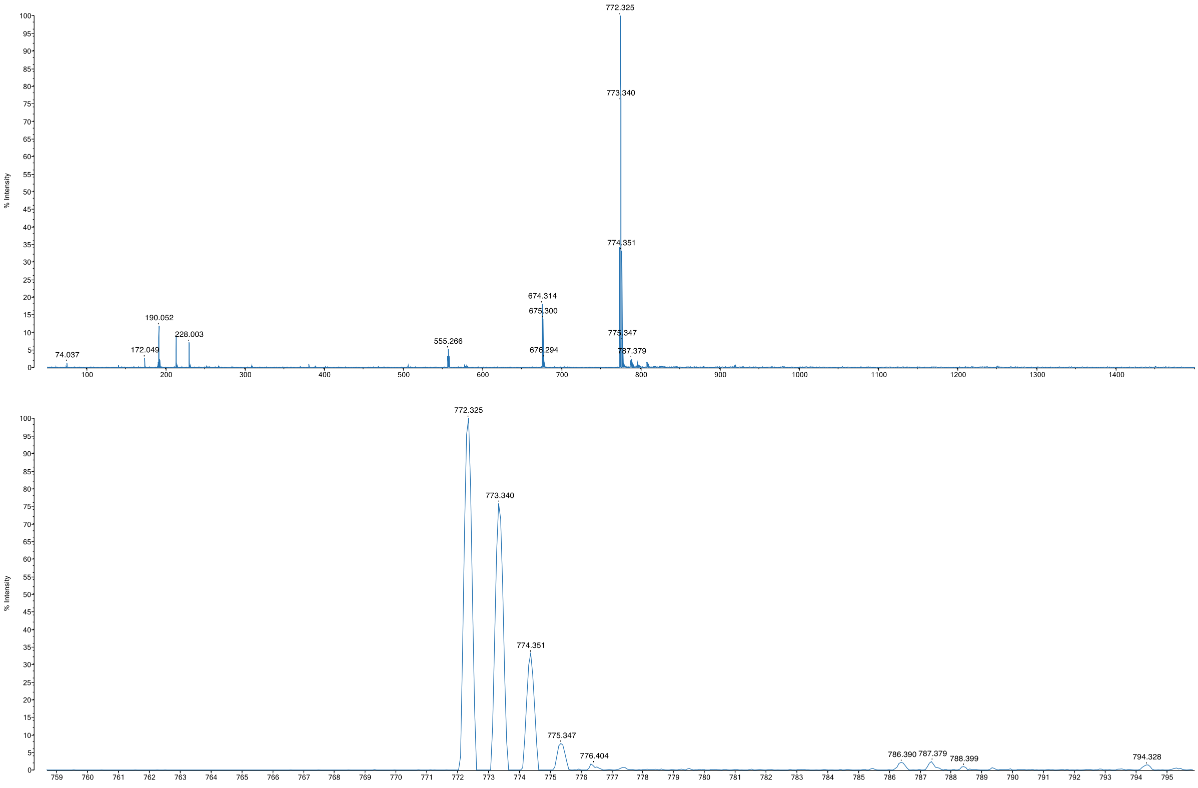

Figure S35. The MALDI-TOF mass spectrum of **2**, and expansion of the base peak.

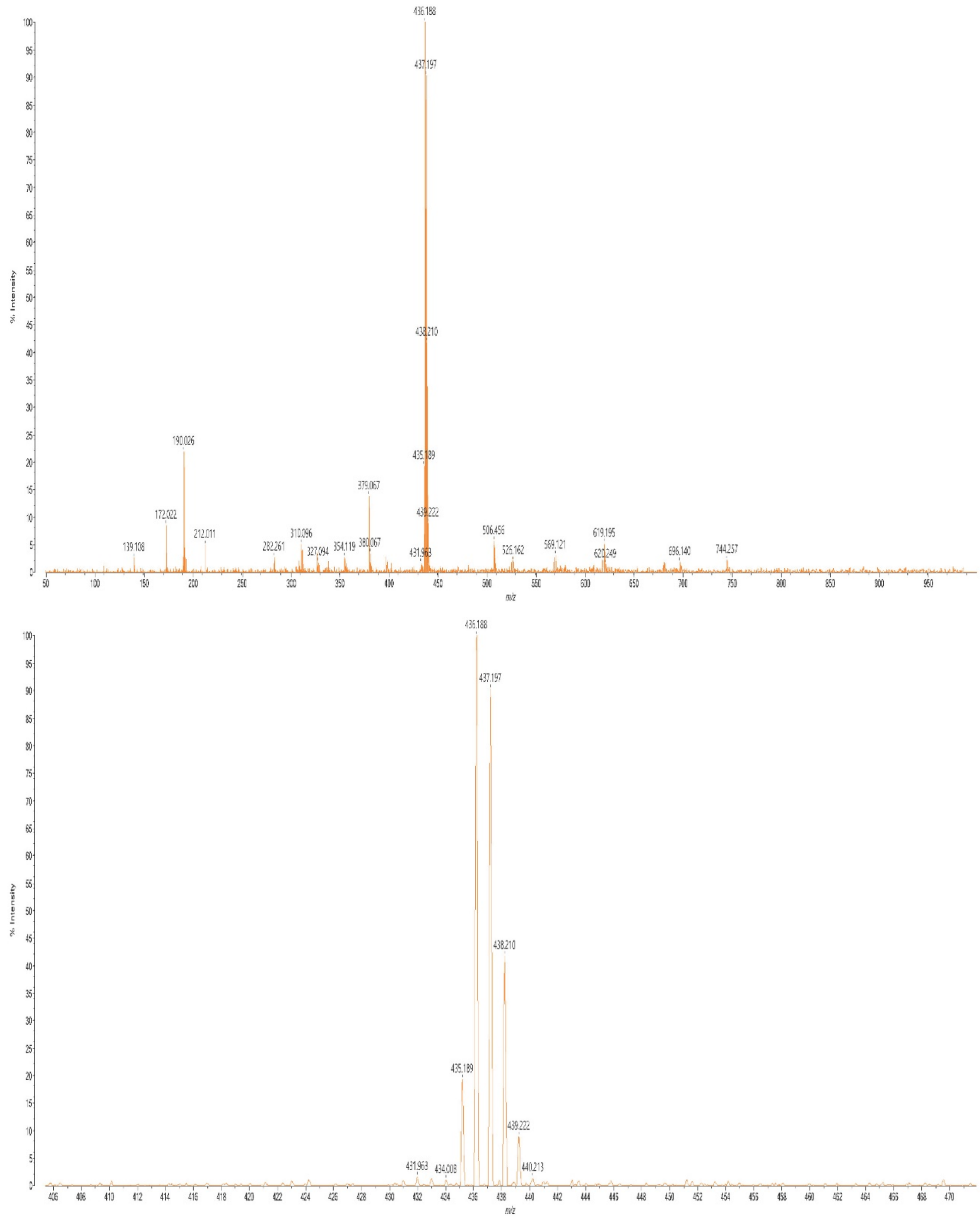

Figure S36. The MALDI-TOF mass spectrum of **P1**, and expansion of the base peak.

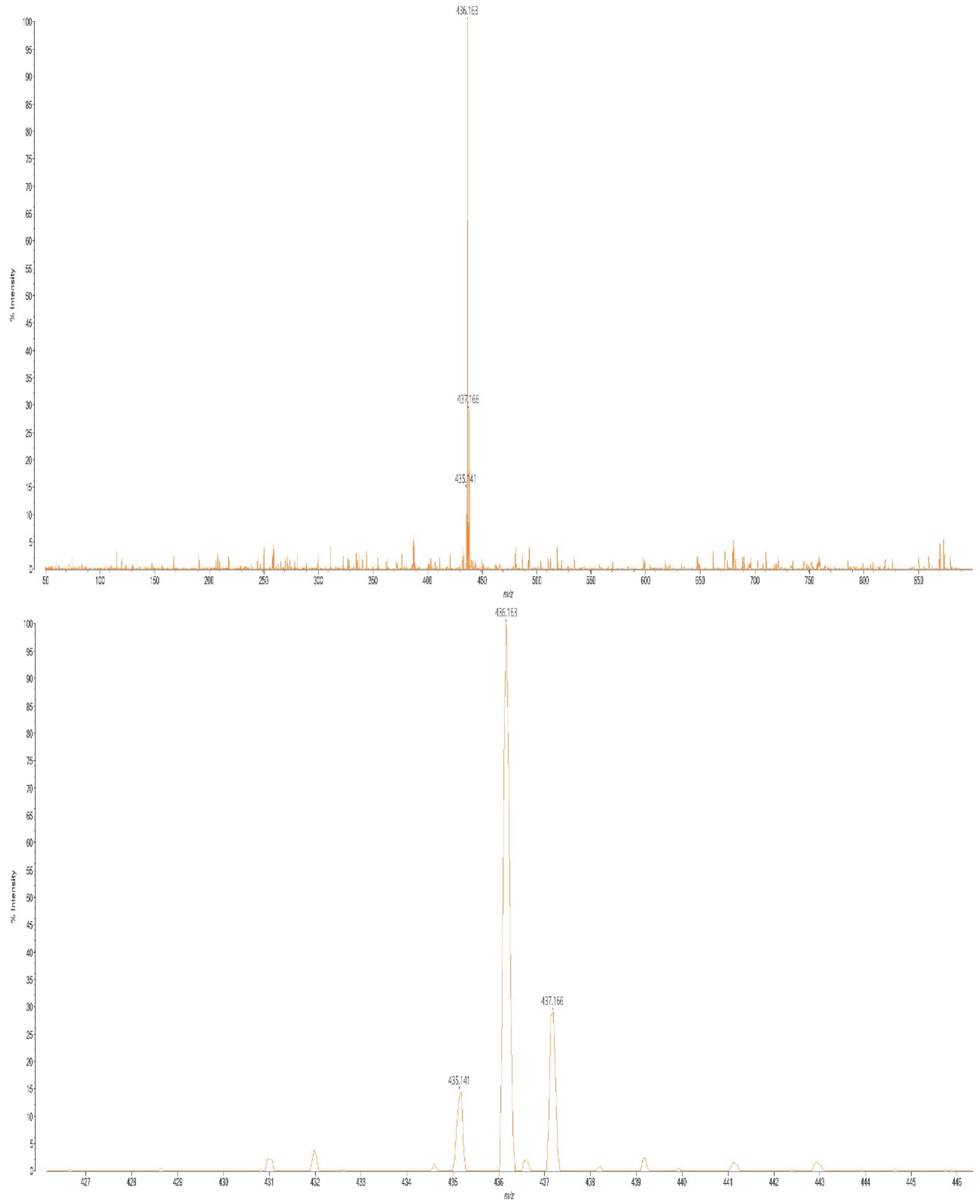

Figure S37. The MALDI-TOF mass spectrum of **P2**, and expansion of the base peak.

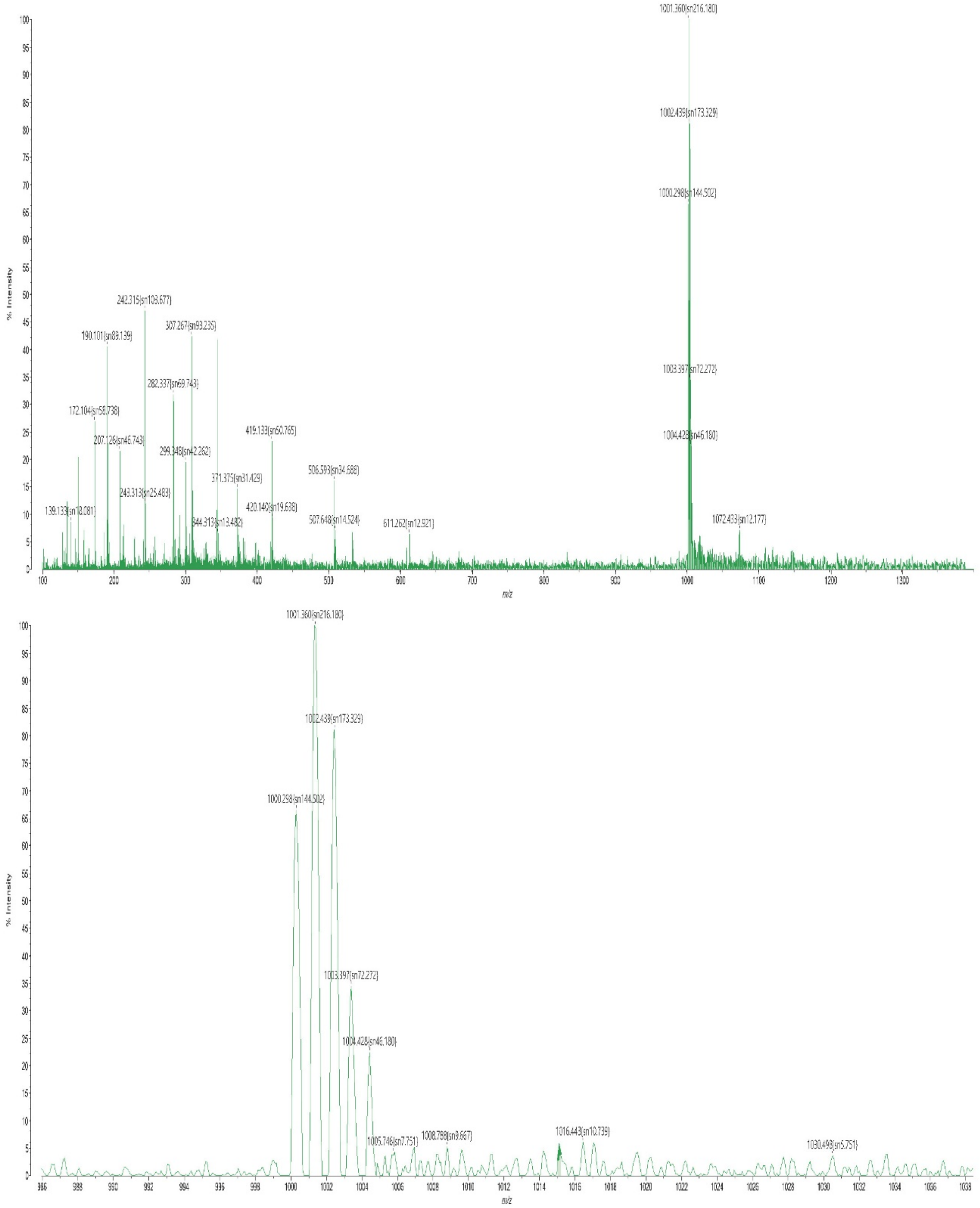

Figure S38. The MALDI-TOF mass spectrum of **3**, and expansion of the base peak.

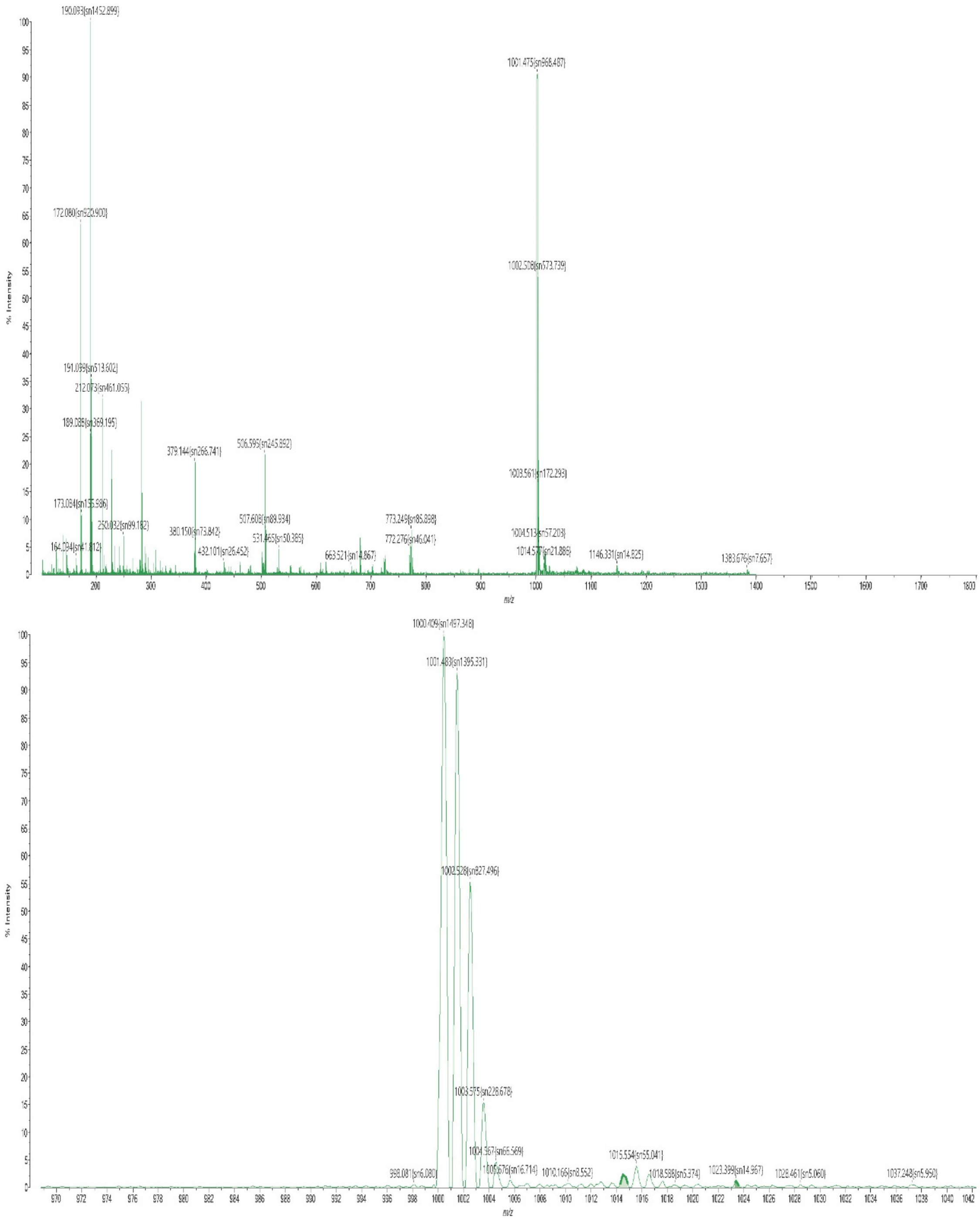

Figure S39. The MALDI-TOF mass spectrum of **4**, and expansion of the $[M+H]^+$ peak. The base peak at m/z = 190 in the upper spectrum arises from the matrix.

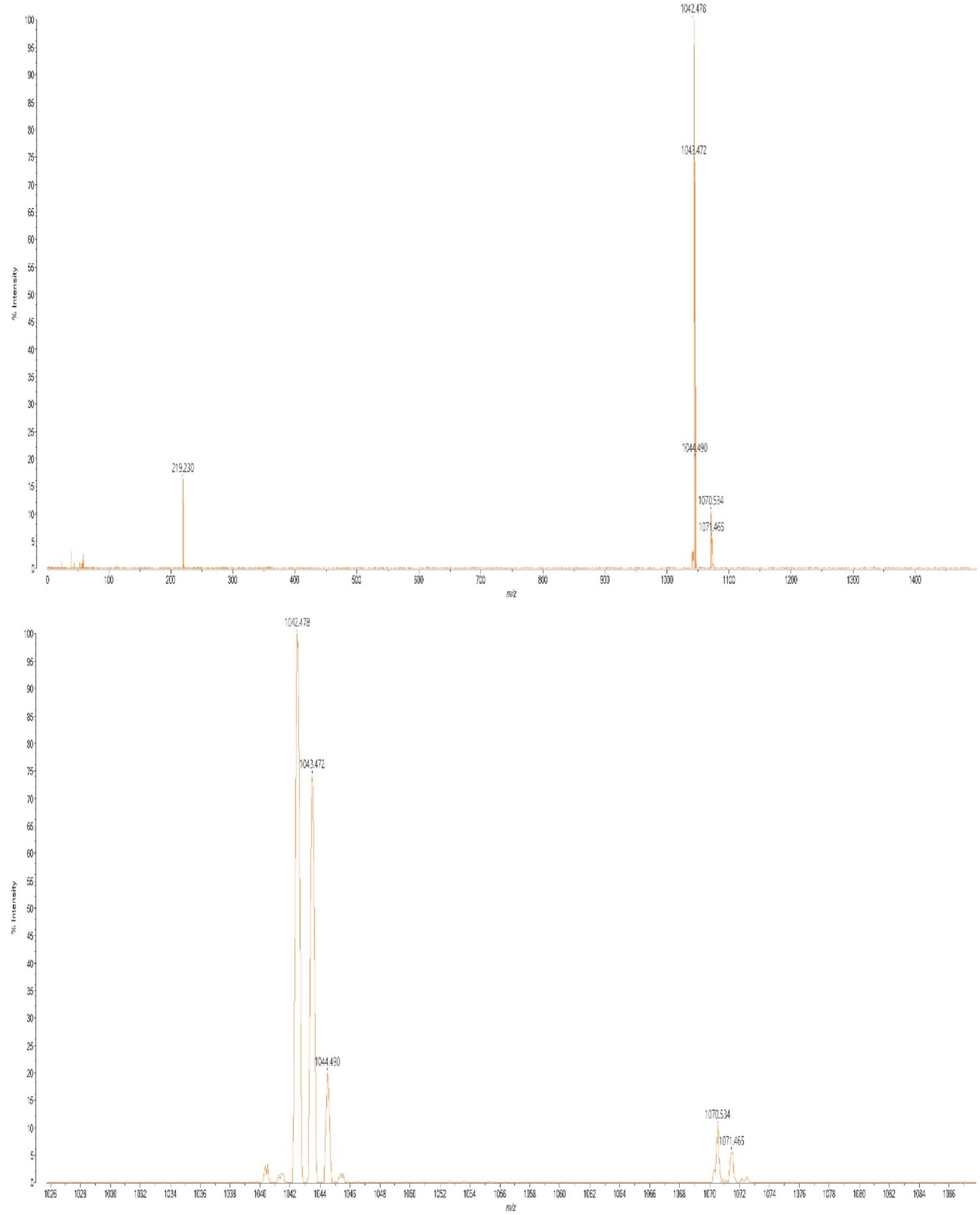

Figure S40. The MALDI-TOF mass spectrum of **5**, and expansion of the base peak.

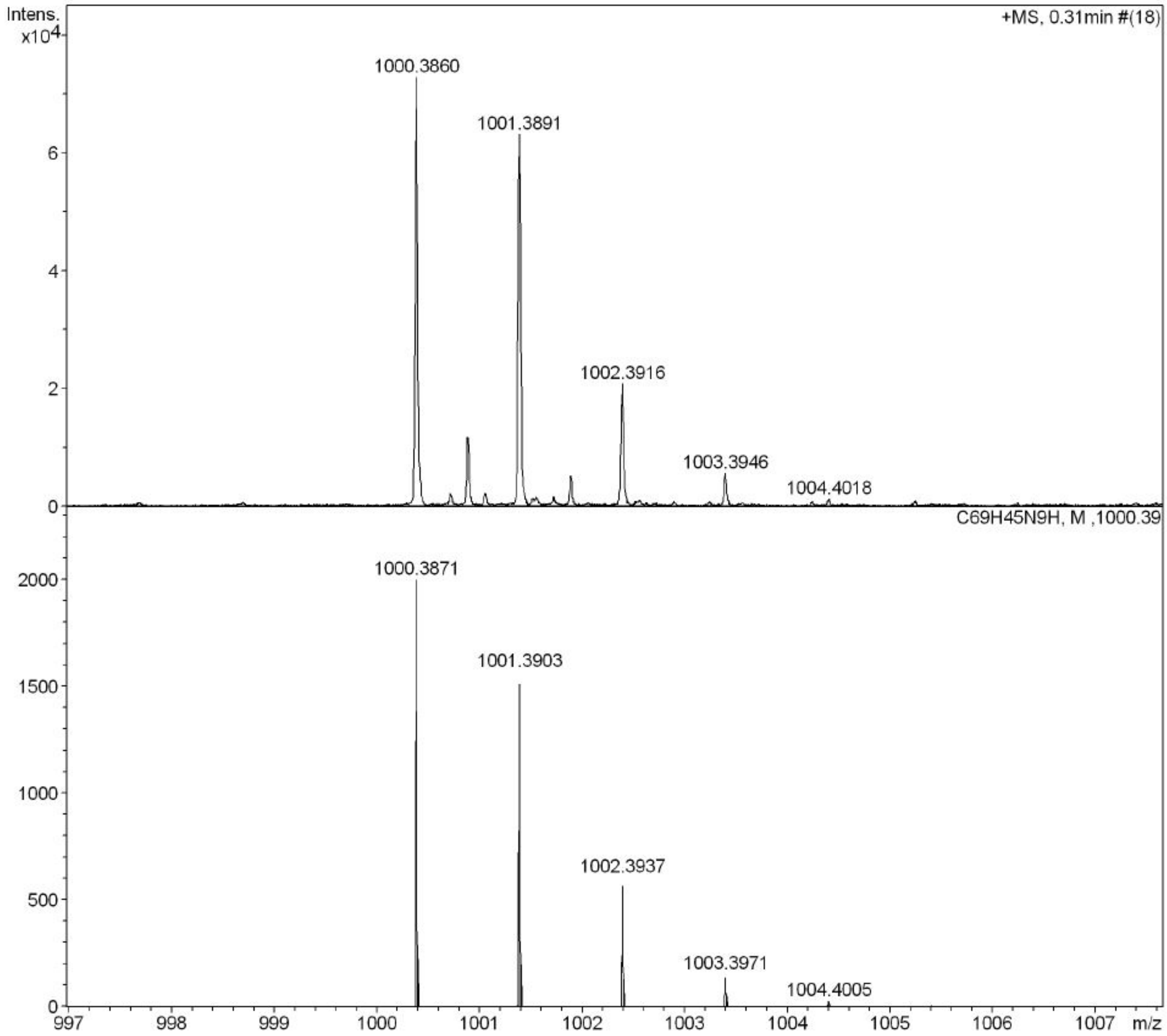

Figure S41. High-resolution mass spectrum of **3** comparing the experimental isotope pattern for the base peak arising from $[M+H]^+$ (top) with the calculated isotope pattern (bottom).

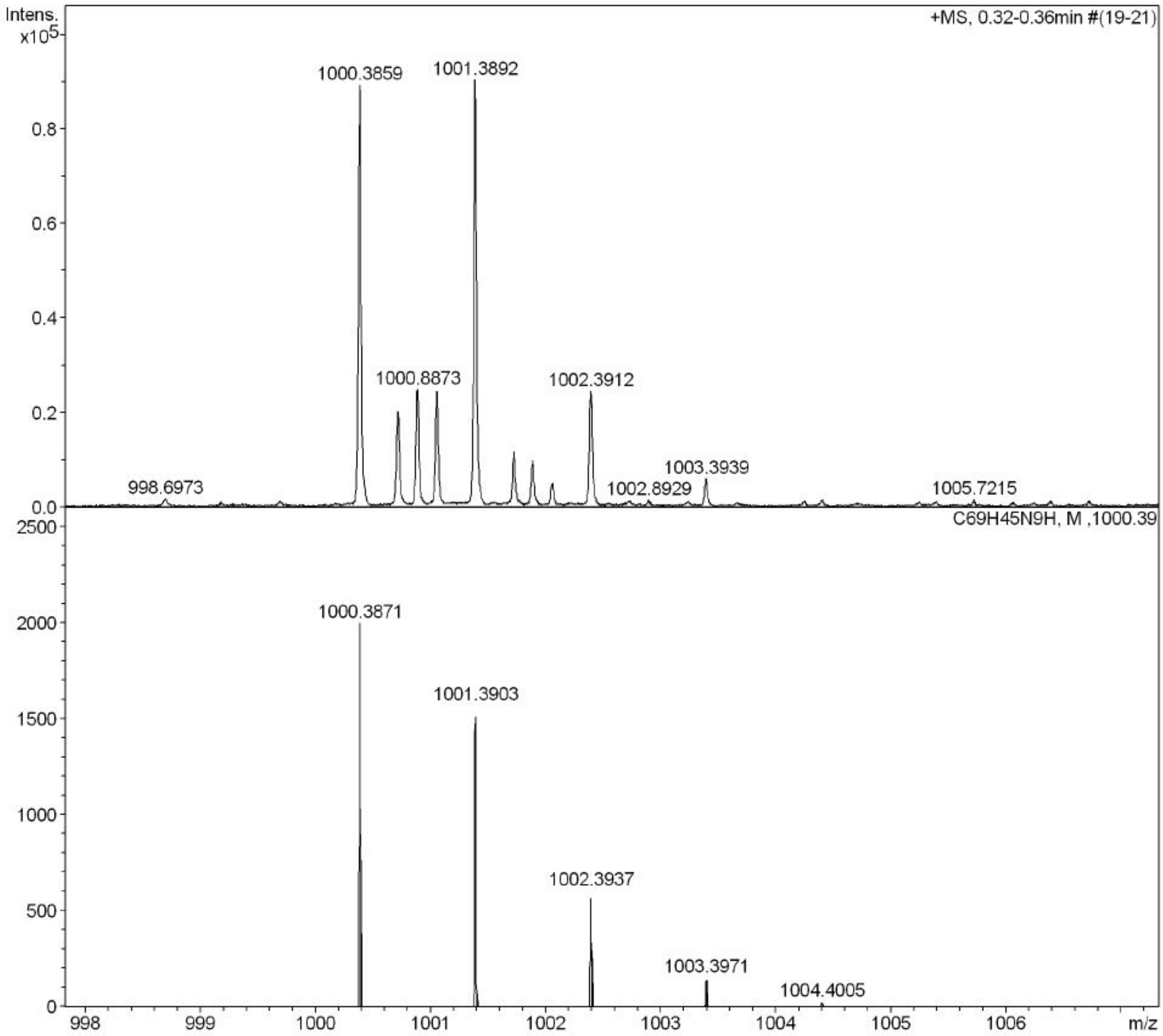

Figure S42. High-resolution mass spectrum of **4** comparing the experimental isotope pattern for the base peak arising from $[M+H]^+$ (top) with the calculated isotope pattern (bottom).

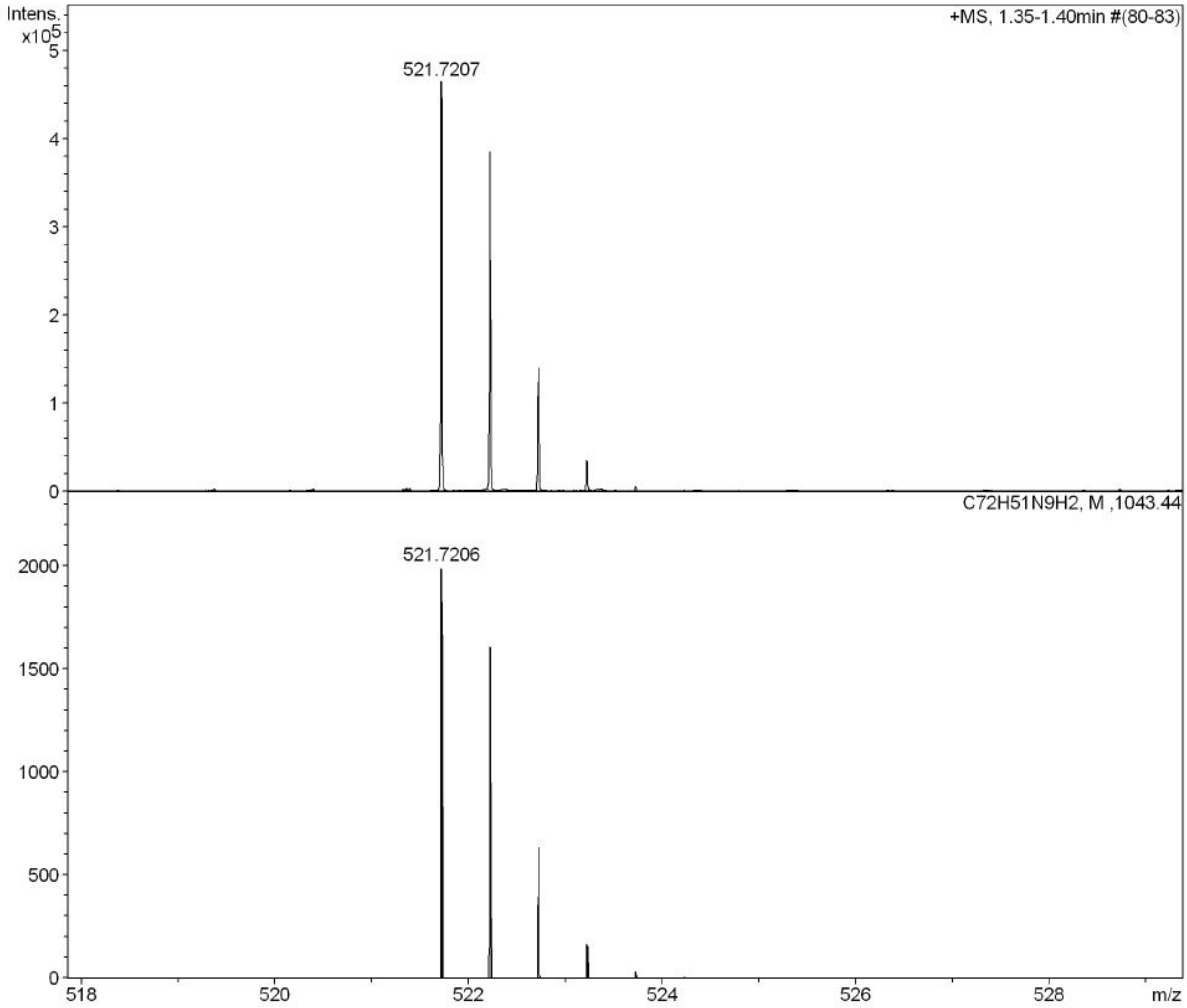

Figure S43. High-resolution mass spectrum of **5** comparing the experimental isotope pattern for the base peak arising from $[M+2H]^{2+}$ (top) with the calculated isotope pattern (bottom).

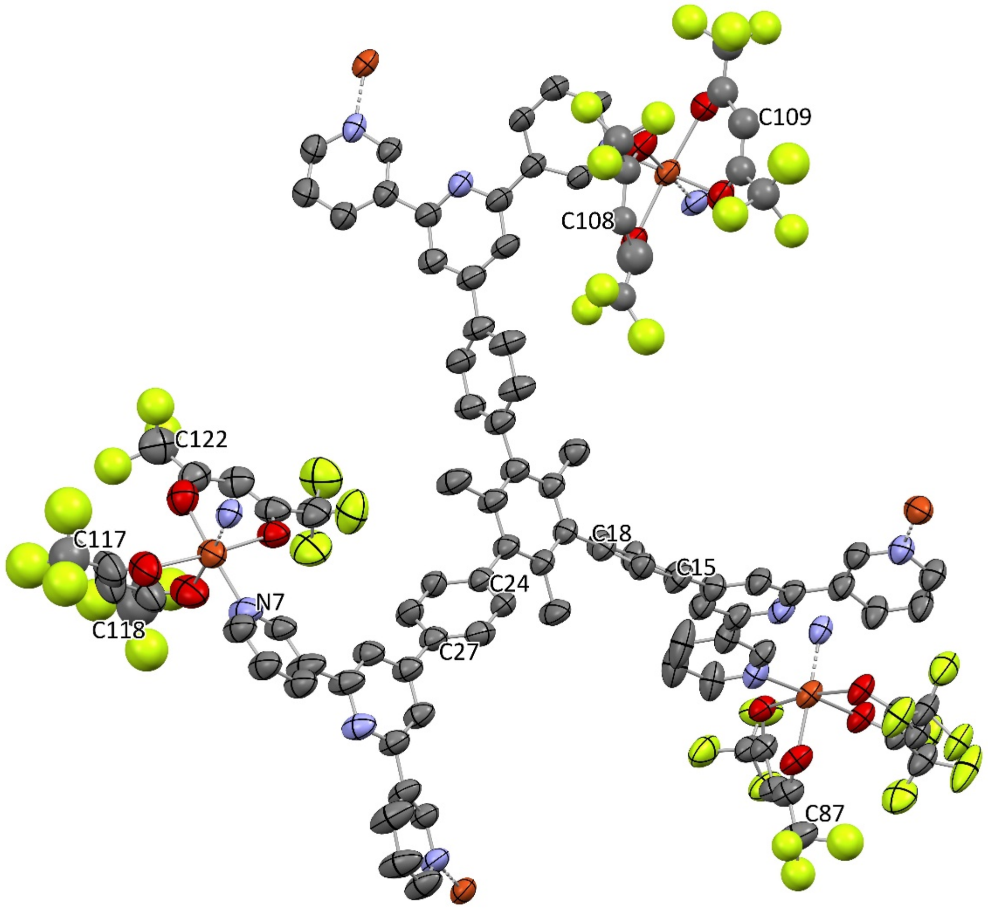

Figure S44. Molecular structure of the asymmetric unit in $[Cu_3(hfacac)_6(\mathbf{5})]_n{\cdot}2.8nC_7H_8{\cdot}0.4nCHCl_3$ with symmetry generated atoms. The backbone of the coordinated [hfacac]⁻ ligand containing C108 and C109, CF_3 groups having C87, C117, C118 and C122, the phenyl spacers containing atoms C15–C18 and C24–C27, the pyridine ring containing N7, and solvent molecules are disordered, and only major occupancies are shown. Disordered parts of the [Hfacac]⁻ ligands were refined isotropically with geometrical restraints. Ellipsoids are plotted at 40% probability. For clarity, H atoms and solvent are omitted.

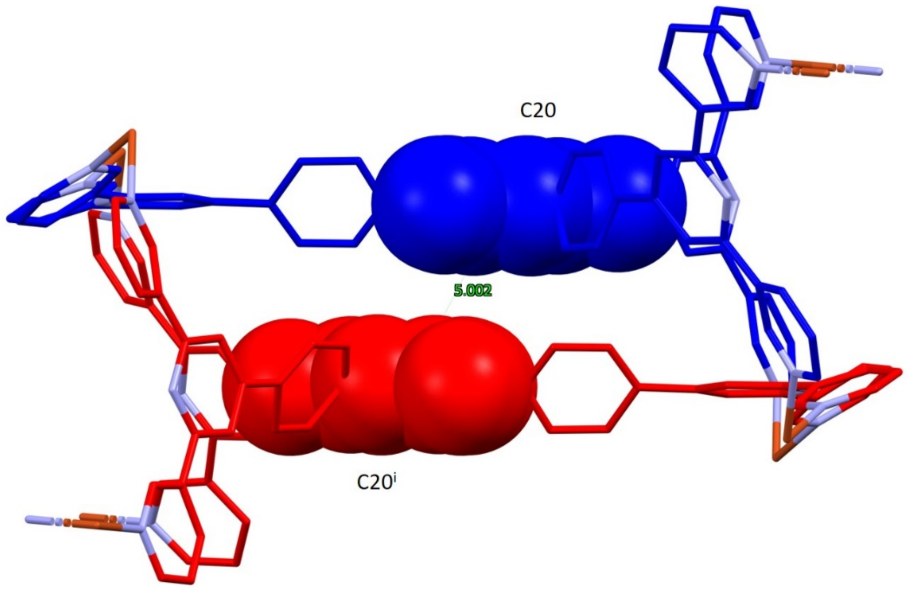

Figure S45. Staggered arrangement of the arene cores within the step. Centroid separations between for pairs of rings containing C20/C20^{i} is 5.00 Å (symmetry code i = 1−*x*, 1−*y*, 1−*z*); see text for discussion. For clarity, H atoms, hfacacs and solvent are omitted, and only major occupancies are shown.

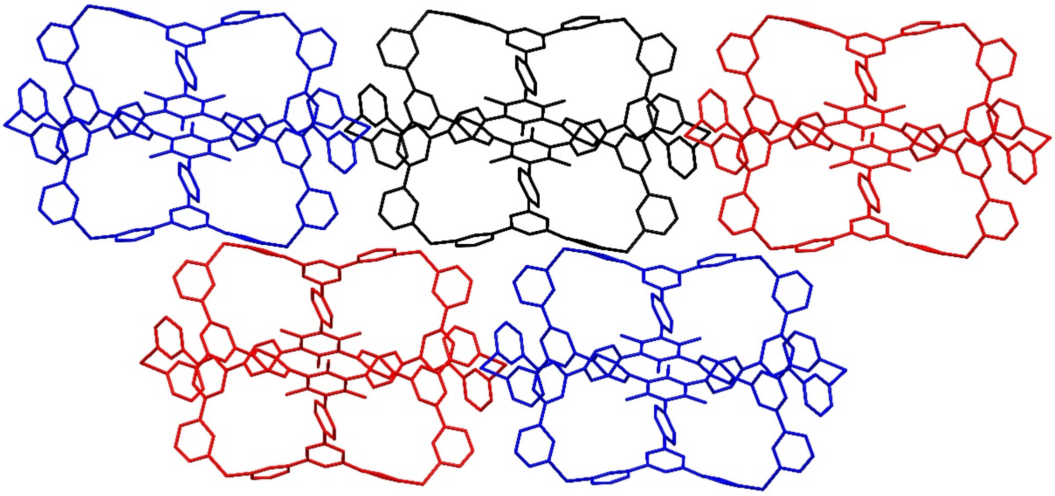

Figure S46. Packing and cross-section of five 1D ladders (alternatively coloured), viewed down the crystallographic *b*-axis along which the 1D-polymers propagate. For clarity, H atoms, [hfacac]⁻ ligands and solvent molecules are omitted, and only major occupancies of disordered atoms are shown.
